# The genome of *Geobacter bemidjiensis*, exemplar for the subsurface clade of *Geobacter *species that predominate in Fe(III)-reducing subsurface environments. 

**DOI:** 10.1186/1471-2164-11-490

**Published:** 2010-09-09

**Authors:** Muktak Aklujkar, Nelson D Young, Dawn Holmes, Milind Chavan, Carla Risso, Hajnalka E Kiss, Cliff S Han, Miriam L Land, Derek R Lovley

**Affiliations:** 1 University of Massachusetts Amherst, Amherst, MA 01003, USA; 2 Department of Energy, Joint Genome Institute, Walnut Creek, CA 94598, USA; 3 Oak Ridge National Laboratory, Oak Ridge, TN 37830, USA

## Abstract

**Background:**

*Geobacter *species in a phylogenetic cluster known as subsurface clade 1 are often the predominant microorganisms in subsurface environments in which Fe(III) reduction is the primary electron-accepting process. *Geobacter bemidjiensis*, a member of this clade, was isolated from hydrocarbon-contaminated subsurface sediments in Bemidji, Minnesota, and is closely related to *Geobacter *species found to be abundant at other subsurface sites. This study examines whether there are significant differences in the metabolism and physiology of *G. bemidjiensis *compared to non-subsurface *Geobacter *species.

**Results:**

Annotation of the genome sequence of *G. bemidjiensis *indicates several differences in metabolism compared to previously sequenced non-subsurface *Geobacteraceae*, which will be useful for *in silico *metabolic modeling of subsurface bioremediation processes involving *Geobacter *species. Pathways can now be predicted for the use of various carbon sources such as propionate by *G. bemidjiensis*. Additional metabolic capabilities such as carbon dioxide fixation and growth on glucose were predicted from the genome annotation. The presence of different dicarboxylic acid transporters and two oxaloacetate decarboxylases in *G. bemidjiensis *may explain its ability to grow by disproportionation of fumarate. Although benzoate is the only aromatic compound that *G. bemidjiensis *is known or predicted to utilize as an electron donor and carbon source, the genome suggests that this species may be able to detoxify other aromatic pollutants without degrading them. Furthermore, *G. bemidjiensis *is auxotrophic for 4-aminobenzoate, which makes it the first *Geobacter *species identified as having a vitamin requirement. Several features of the genome indicated that *G. bemidjiensis *has enhanced abilities to respire, detoxify and avoid oxygen.

**Conclusion:**

Overall, the genome sequence of *G. bemidjiensis *offers surprising insights into the metabolism and physiology of *Geobacteraceae *in subsurface environments, compared to non-subsurface *Geobacter *species, such as the ability to disproportionate fumarate, more efficient oxidation of propionate, enhanced responses to oxygen stress, and dependence on the environment for a vitamin requirement. Therefore, an understanding of the activity of *Geobacter *species in the subsurface is more likely to benefit from studies of subsurface isolates such as *G. bemidjiensis *than from the non-subsurface model species studied so far.

## Background

*Geobacter bemidjiensis *is a member of the *Geobacteraceae*, a family of Fe(III)-respiring *Deltaproteobacteria *that are of interest for their role in cycling of carbon and metals in aquatic sediments and subsurface environments as well as the bioremediation of organic- and metal-contaminated groundwater and the harvesting of electricity from complex organic matter [1,2]. It was isolated from subsurface sediments in Bemidji, Minnesota, near a site where aromatic hydrocarbons were being degraded naturally [3]. *G. bemidjiensis *is a member of the phylogenetic cluster designated subsurface clade 1, which predominates in a diversity of subsurface environments in which dissimilatory Fe(III) reduction is an important process [4]. Environmental proteomic studies have demonstrated that *Geobacter *species closely related to *G. bemidjiensis *were metabolically active during the *in situ *bioremediation of uranium-contaminated groundwater [5].

Preliminary studies have suggested that genome-scale metabolic modeling of *Geobacter *species [6,7] may aid in predicting the response of subsurface *Geobacter *species to subsurface bioremediation strategies [8,9]. However, it is not known whether the metabolic potential of subsurface *Geobacter *species is essentially the same as that of non-subsurface *Geobacter *species, or significantly different. Therefore, comparative analysis of the genome of a representative of the subsurface clade 1 *Geobacter *species with the curated genomes of two non-subsurface *Geobacter *species, *Geobacter sulfurreducens *and *Geobacter metallireducens *[10,11], was carried out to improve predictive modeling of the responses of *Geobacteraceae *to efforts to stimulate bioremediation of organic and metal contaminants in the subsurface.

## Results and Discussion

### Contents of the *G. bemidjiensis *genome

The automated annotation process identified 4040 protein-coding genes and 76 ribonucleic acid (RNA) genes in the genome of 4615150 bp. During manual curation, 56 genes were discarded, 40 genes were reannotated as pseudogenes, and another 79 protein-coding genes, 28 pseudogenes, and 778 non-protein-coding features were identified. Protein sequence alignments demonstrated that 27 pseudogenes were frameshifted within runs of five or more identical bases, where DNA replication is most error-prone, and seven of these are polymorphisms where the minor alleles contain no frameshift, indicating a subpopulation of cells that can produce functional proteins from these genes (Additional file 1: Table S1). Of the 4023 intact protein-coding genes in the *G. bemidjiensis *genome, 148 hypothetical proteins (3.6%) have no match in any other genome, including that of *Geobacter *sp. M21; a further 87 conserved hypothetical proteins were found only in these two closely related genomes.

### Metabolism of pyruvate

An appropriate place to begin to compare metabolism between the subsurface exemplar *G. bemidjiensis *and the representative non-subsurface species *G. sulfurreducens *and *G. metallireducens *is with the central metabolic reactions that interconvert pyruvate and acetyl-CoA. Like other *Geobacteraceae*, *G. bemidjiensis *possesses two sets of genes encoding pyruvate dehydrogenase complexes (Gbem_2257 and Gbem_2251-Gbem_2250; Gbem_0459-Gbem_0461), which irreversibly convert pyruvate to acetyl-CoA. The ability of *G. sulfurreducens *to reverse this reaction and derive biomass from acetyl-CoA has been attributed to a homodimeric pyruvate:ferredoxin/flavodoxin oxidoreductase [12], for which homologs exist in all *Geobacteraceae *including *G. bemidjiensis *(Gbem_0209). *G. bemidjiensis *has an additional pyruvate:ferredoxin oxidoreductase (Gbem_4034), more closely related to those of sulfate-reducing bacteria. In addition to gluconeogenesis and oxidative decarboxylation to acetyl-CoA, a third fate of pyruvate in *G. bemidjiensis *may be oxidative decarboxylation to acetate by a putative quinone-reducing pyruvate decarboxylase (Gbem_0287) that is 32% identical to the *E. coli *enzyme [13]. Thus, metabolism of pyruvate may be more complex in *G. bemidjiensis *than in non-subsurface *Geobacter *species.

### Metabolism of propionate

**Figure 1:**
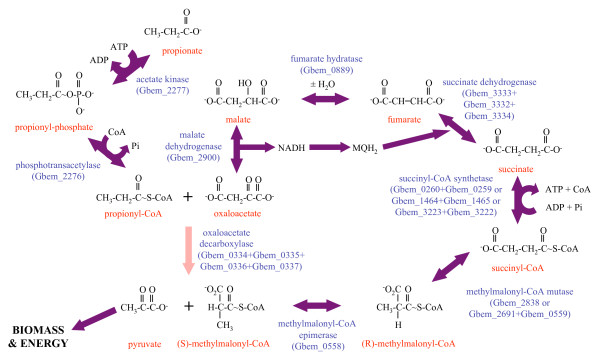
**Predicted pathway for conversion of propionate to pyruvate by *G. bemidjiensis***. Eight steps are catalyzed by enzymes that have orthologs in both *G. sulfurreducens *and *G. metallireducens *(purple arrows). The only enzyme unique to *G. bemidjiensis *(pink arrow) is oxaloacetate decarboxylase, which simultaneously functions as propionyl-CoA carboxylase to carry out an earlier step of the cyclical pathway.

*G. bemidjiensis *and *G. metallireducens *utilize propionate as an electron donor, whereas *G. sulfurreducens *cannot. Analysis of the genome suggests that *G. bemidjiensis *utilizes propionate by converting it to pyruvate in ten steps (Figure 1): (1-2) activation to propionyl-CoA by the same enzymes that activate acetate; (3) carboxylation to (S)-methylmalonyl-CoA by a biotin-dependent propionyl-CoA carboxyltransferase (Gbem_0335) that is 51% identical to the 12 S subunit of the *Propionibacterium freudenreichii *methylmalonyl-CoA:pyruvate carboxyltransferase enzyme complex [14]; (4-5) epimerization and rearrangement to succinyl-CoA; (6) hydrolysis to succinate; (7-9) oxidation to oxaloacetate; and (10) decarboxylation to pyruvate by an oxaloacetate decarboxylase (Gbem_0334) that is 60% identical to the 5 S subunit of the *P. freudenreichii *methylmalonyl-CoA:pyruvate carboxyltransferase complex [14], with concomitant transfer of the carboxyl group to propionyl-CoA (see step 3 above). The carboxyltransferase reaction, possibly involving a conserved hypothetical protein (Gbem_0336) and a biotin attachment domain protein (Gbem_0337) encoded within the same predicted operon as the two enzymes, avoids the energetic cost of 1 ATP associated with other biotin-dependent carboxylations of acyl-CoA substrates. The energy recovered from hydrolysis of succinyl-CoA to succinate may be used for the initial activation of propionate, either in the form of ATP or possibly through direct transfer of CoA by one of several uncharacterized acyl-CoA:carboxylate CoA transferases (Gbem_1430, Gbem_1439, Gbem_3573). This energy-efficient pathway contrasts with the proposed pathway in the non-subsurface species *G. metallireducens *[10,15], in which no energy is recovered from thioester hydrolysis of propionyl-CoA by 2-methylcitrate synthase. Accordingly, reliable predictions of the metabolic activity of *Geobacter *species in subsurface environments amended with propionate will depend on models based on *G. bemidjiensis *rather than *G. metallireducens*.

### Fatty acid metabolism

The *G. bemidjiensis *genome encodes many enzymes of acyl-CoA metabolism (Additional file 2: Table S2). This multiplicity of genes suggests that in addition to its known short-chain organic acid electron donors (butyrate, isobutyrate and valerate), *G. bemidjiensis *may also be able to utilize longer fatty acids as sources of carbon and electrons. Indeed, *G. bemidjiensis *and the other genomes of subsurface *Geobacter *species possess a very-long-chain fatty acyl-CoA dehydrogenase (*fadE *Gbem_2128) 50% identical to that of *Bacillus subtilis *[16], which is absent from *G. sulfurreducens *and *G. metallireducens*. Thus, the metabolism of fatty acids by subsurface *Geobacter *species may be better understood by examining *G. bemidjiensis *rather than non-subsurface relatives.

### Growth of *G. bemidjiensis *by disproportionation of fumarate

**Figure 2:**
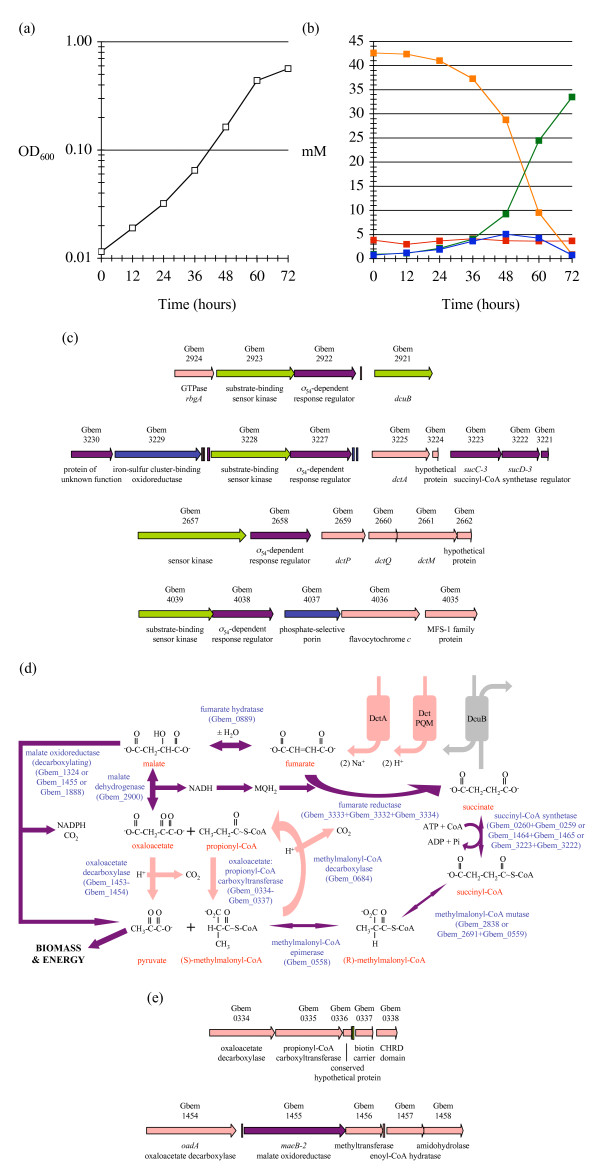
**Growth of *G. bemidjiensis *by disproportionation of fumarate**. **(a) **Growth of *G. bemidjiensis *with fumarate as sole electron donor and electron acceptor (white squares). The mean of quadruplicate measurements is shown; variation was negligible. **(b) **High-pressure liquid chromatography measurements showing consumption of fumarate (orange squares), excretion of succinate (green squares), transient excretion of malate (blue squares) and unaffected trace amounts of extracellular acetate (red squares) during growth of *G. bemidjiensis *with fumarate alone. **(c) **Organization of fumarate transporter genes. Genes unique to *G. bemidjiensis *(pink arrows) are found in proximity to genes that have orthologs in either *G. sulfurreducens *(green arrows) or *G. metallireducens *(blue arrows), or both (purple arrows), along with multicopy nucleotide sequence features (rectangles; same colour code). The fumarate transporters are encoded by *dcuB *(dicarboxylic acid exchanger), *dctA *(sodium-dependent), and *dctPQM *(proton-dependent). **(d) **Predicted pathways of fumarate disproportionation by conversion to pyruvate. Enzymes that have orthologs in both *G. sulfurreducens *and *G. metallireducens *(purple arrows) constitute the decarboxylating malate oxidoreductase pathway, which is inactive when the only means of fumarate transport is the fumarate/succinate exchanger DcuB, as in *G. sulfurreducens *(grey arrows). The unique fumarate transporters and enzymes of *G. bemidjiensis *(pink arrows) make possible two other pathways involving different oxaloacetate decarboxylases. **(e) **Organization of oxaloacetate decarboxylase genes; the colour code is the same as in (c).

*G. bemidjiensis*, like *G. sulfurreducens*, can utilize fumarate as an electron acceptor in combination with acetate and other electron donors [3]. Consistent with its previously described ability to utilize malate as an electron donor and carbon source, *G. bemidjiensis *also grows when provided with only fumarate (Figure 2a), as was previously reported for *Geobacter bremensis *[17]. Existing metabolic models of (non-subsurface) *Geobacter *species do not include this capability; therefore, we examined it more closely. Chromatographic analysis of culture filtrates indicates that 79% of the supply of fumarate serves as electron acceptor and is excreted as succinate, implying that another 13% of the fumarate is completely oxidized to carbon dioxide (Figure 2b). Biomass is inferred to derive from the remaining 8% of the fumarate supply. A small amount of malate is transiently excreted when consumption of fumarate is steepest (Figure 2b). Unlike *Desulfovibrio vulgaris *[18] and *Rhodoferax ferrireducens *[19], *G. bemidjiensis *does not excrete acetate, even at early stages (Figure 2b), indicating that complete oxidation of fumarate is energetically more favourable than partial oxidation to acetyl-CoA followed by substrate-level phosphorylation.

Either the sodium/fumarate symporter DctA (Gbem_3225) that is 61% identical to the *Salmonella typhimurium *transporter [20] or the proton/fumarate symporter complex DctPQM (Gbem_2659-Gbem_2661) with 40% to 63% sequence identity to characterized homologs in *Rhodobacter capsulatus *[21] may be a prerequisite for disproportionation of fumarate. Both transporters, which are absent from *G. sulfurreducens*, allow import of fumarate without the concomitant export of succinate required by the dicarboxylate exchange transporter DcuB (Gbem_2921, orthologous to GSU2751). Notably, the *dcuB *and *dctA *genes are each located 3' of a pair of genes encoding a periplasmic substrate-binding sensor histidine kinase and response regulator that are highly similar (Figure 2c); a third pair is located 5' of a phosphate-selective porin (Gbem_4037, orthologous to Gmet_1042). The three sensor kinases (Gbem_2923; Gbem_3228; Gbem_4039) are 47% to 56% identical and the three response regulators (Gbem_2922; Gbem_3227; Gbem_4038) are 65% to 69% identical, suggesting that *G. bemidjiensis *has developed parallel signalling pathways possibly linked to dicarboxylate transport.

In *D. vulgaris*, oxidation of fumarate proceeds through the decarboxylating malate oxidoreductase reaction (B. Giles and J. Wall, personal communication), but it would be surprising if this were the predominant pathway in *G. bemidjiensis *(Figure 2d). When *G. sulfurreducens *respires fumarate, the activity of its two malate oxidoreductases (NADP-dependent *maeB *GSU1700 and NAD-dependent *mleA *GSU2308) must be kept much lower than that of malate dehydrogenase, which converts malate to oxaloacetate, because the equal exchange of succinate for fumarate by DcuB requires that any malate that is decarboxylated to pyruvate must be replaced by carboxylation of pyruvate to oxaloacetate at the expense of one ATP, which is prohibitive [12]. *G. bemidjiensis *possesses three *maeB *genes, all closely related to GSU1700, and no *mleA *gene. It is possible that one or more of these isozymes are upregulated during disproportionation of fumarate. However, complete oxidation of fumarate through acetyl-CoA using the malate oxidoreductases rather than malate dehydrogenase would result in NADH and NADPH being produced in a ratio of 3:2 rather than 4:1, requiring rerouting of reducing equivalents to meet energy demand. A more reasonable hypothesis is that conversion of fumarate to pyruvate is accomplished through decarboxylation of oxaloacetate by two parallel pathways (Figure 2d; gene diagrams in Figure 2e).

One oxaloacetate decarboxylase complex transfers the carboxyl group to propionyl-CoA as detailed above, forming (S)-methylmalonyl-CoA. In contrast with this same enzyme complex's predicted cyclic involvement in oxidation of propionate to pyruvate (see above, Figure 1), its catalysis of this step in the fumarate oxidation pathway requires that (S)-methylmalonyl-CoA be recycled to propionyl-CoA by methylmalonyl-CoA decarboxylase (Gbem_0684) (Figure 2d). The other oxaloacetate decarboxylase (*oadA *Gbem_1454) is 60% identical to the catalytic subunit of the sodium-translocating oxaloacetate decarboxylase of *Klebsiella pneumoniae *[22]. No homologs of the other two subunits were found in *G. bemidjiensis*, indicating that decarboxylation of oxaloacetate is not coupled to a sodium pump.

The malate oxidoreductase and oxaloacetate decarboxylase pathways also account for the ability of *G. bemidjiensis *to grow with malate and succinate as sources of carbon and electrons. Thus, the genome annotation of *G. bemidjiensis *offers insight into its unique capability to metabolize dicarboxylic acids without excreting acetate, which could not be predicted correctly from studies of either the non-subsurface *Geobacter *genomes or non-*Geobacter *species.

### Possible carbon dioxide fixation via citrate lyase

**Figure 3:**
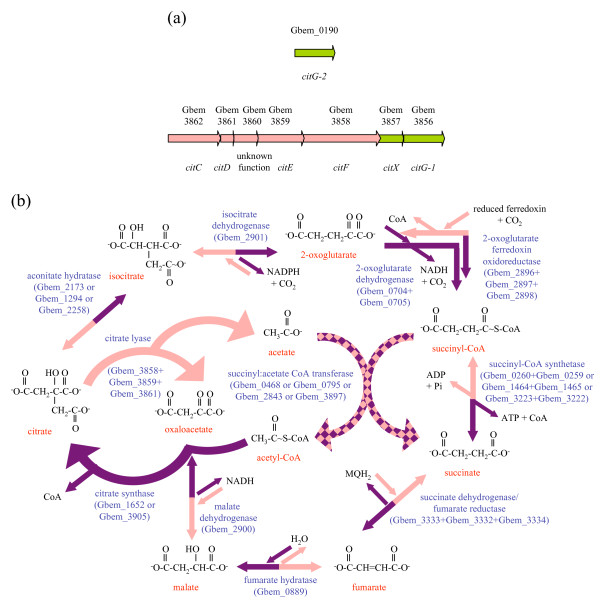
**Carbon dioxide fixation in *G. bemidjiensis***. **(a) **The citrate lyase gene cluster and separately located gene Gbem_0190; some components are unique to *G. bemidjiensis *(pink arrows) and others have orthologs in *G. sulfurreducens *(green arrows). The catalytic subunits are encoded by *citEF*, the acyl carrier protein by *citD*, the cofactor synthesis enzyme by *citG-1 *and *citG-2*, the cofactor-transferring enzyme by *citX*, and the priming enzyme by *citC*. **(b) **The oxidative and reductive TCA cycles. Most enzymes that catalyze reductive reactions unique to *G. bemidjiensis *(pink arrows) also catalyze the oxidative reactions common to *G. sulfurreducens *and *G. metallireducens *(purple arrows). The exceptions are citrate lyase (reductive direction only) and citrate synthase and 2-oxoglutarate dehydrogenase (oxidative direction only).

Addition of acetate to the subsurface selectively stimulates the growth of *Geobacteraceae*, which derive electrons by conversion of acetate to carbon dioxide through the TCA cycle [23]. The presence of a *citCDEFXG *gene cluster (Figure 3a) encoding the citrate lyase enzyme in *G. bemidjiensis *(Gbem_3862-Gbem_3856) suggests that this species might also operate the TCA cycle in reverse, fixing two molecules of carbon dioxide and generating acetate (Figure 3b). This gene cluster is not found in the genomes of the non-subsurface species *G. sulfurreducens *and *G. metallireducens*. Preliminary data indicate that *G. bemidjiensis *can grow on Fe(III) oxides plus hydrogen, without any carbon source other than carbon dioxide (D. Holmes and C. Risso, unpublished), although the involvement of citrate lyase remains to be established.

A reverse TCA cycle requires enzymes capable of carrying out two carboxylations: conversion of succinyl-CoA to 2-oxoglutarate and conversion of 2-oxoglutarate to isocitrate (Figure 3b). The first of these conversions has been inferred from a carbon flux analysis study of *G. metallireducens *[24] and may be attributed to 2-oxoglutarate:ferredoxin oxidoreductase (Gbem_2896-Gbem_2899). The second conversion may be catalyzed by isocitrate dehydrogenase (Gbem_2901), which is 71% identical to the *Chlorobium limicola *enzyme that is known to be reversible [25]. These genes form a cluster that includes malate dehydrogenase (Gbem_2900), suggesting that flux of oxaloacetate through the reverse TCA cycle may be coordinated.

The presence of homologous gene clusters in *Pelobacter propionicus *and *Desulfuromonas acetoxidans *suggests that citrate lyase was present in the common ancestor of the *Geobacteraceae*, and was lost by most species of the genus *Geobacter*. Interestingly, the 2'-(5''-triphosphoribosyl)-3'-dephospho-CoA synthase gene of the cluster, which is duplicated in *G. bemidjiensis *(*citG-1 *Gbem_3856, *citG-2 *Gbem_0190), is present in *G. sulfurreducens *(GSU0806), along with the gene for the enzyme that transfers 2'-(5''-triphosphoribosyl)-3'-dephospho-CoA to the acyl carrier protein of citrate lyase (*citX *Gbem_3857 = GSU0807), but the genes encoding structural components of citrate lyase are absent, suggesting that 2'-(5''-triphosphoribosyl)-3'-dephospho-CoA may have a second function unrelated to citrate lyase.

Both succinyl:acetate CoA transferase isozymes of *G. sulfurreducens *are doubly present in *G. bemidjiensis *(*ato-3 *Gbem_0795 is a duplicate of *ato-1 *Gbem_0468, and *ato-4 *Gbem_3897 is a duplicate of *ato-2 *Gbem_2843). One possible explanation for this is that the duplicates have distinct functions in the oxidative and reductive TCA cycles (Figure 3b). Another possibility is that each of the duplicate citrate synthases (Gbem_1652, Gbem_3905) utilizes the acetyl-CoA produced by a different pair of isozymes. Although these details remain to be worked out, the TCA cycle on the whole appears to be more complex in the subsurface isolate *G. bemidjiensis *than in the non-subsurface *Geobacter *species examined to date.

### Carbon monoxide dehydrogenases and associated hydrogenase

**Figure 4:**
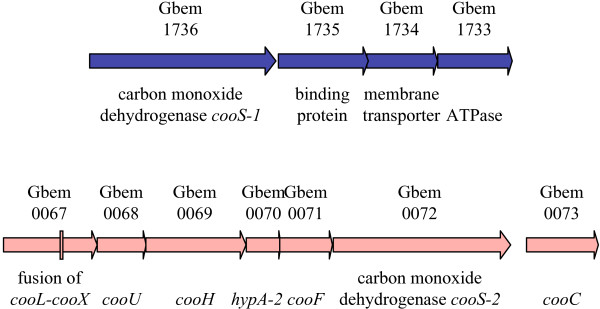
**The carbon monoxide dehydrogenase gene clusters**. One cluster has orthologs in *G. metallireducens *(blue arrows), and the other is unique to *G. bemidjiensis *(pink arrows) and contains an intragenic multicopy nucleotide sequence (pink rectangle). The carbon monoxide dehydrogenase-associated hydrogenase subunits are encoded by *cooLXUH*, the nickel insertion protein by *hypA-2*, an accessory protein by *cooC*, and an iron-sulfur cluster-binding oxidoreductase that transfers electrons from carbon monoxide dehydrogenase to the hydrogenase is encoded by *cooF*.

Like the *G. metallireducens *genome, that of *G. bemidjiensis *encodes a carbon monoxide dehydrogenase (*cooS-1 *Gbem_1736) alongside an ABC transporter complex (Gbem_1735-Gbem_1733) of unknown substrate specificity (Figure 4). The presence of this gene suggests that *G. bemidjiensis *and *G. metallireducens *may be capable of carbon monoxide cycling (fermentative production of carbon monoxide followed by re-oxidation) under some conditions, as has been reported for *D. vulgaris *[26]. In addition, *G. bemidjiensis *possesses a cluster of genes (*cooLUH*-*hypA*-*cooFSC *Gbem_0067-Gbem_0074) with closely related homologs in *Carboxydothermus hydrogenoformans *[27], encoding a carbon monoxide dehydrogenase and associated hydrogenase (Figure 4). Thus, *G. bemidjiensis *may couple oxidation of exogenous carbon monoxide to formation of hydrogen from intracellular protons to establish a proton gradient. The hydrogen and some of the carbon dioxide released by this reaction may be fixed subsequently by the reverse TCA cycle. Genome-based metabolic models of the non-subsurface *Geobacter *species lack this aspect of carbon metabolism of *G. bemidjiensis*.

### Glucose as electron donor

Several unique genes discovered in the *G. bemidjiensis *genome suggested that *G. bemidjiensis *should be able to utilize glucose and galactose as carbon sources. These genes encode a glucose/galactose transporter (*gluP *Gbem_3671) 55% identical to that of *Brucella abortus *[28], a putative glucose 6-kinase (Gbem_2002) 33% identical to that of *E. coli *[29], a galactose 1-kinase (Gbem_4019) 35% identical to that of *E. coli *[30], and a uridine 5'-diphosphate (UDP)-glucose:galactose-1-phosphate uridylyltransferase (Gbem_4017) 32% identical to that of *Thermotoga maritima *[31]. Most *Geobacteraceae*, which do not utilize glucose and galactose, possess only a putative glucose 6-kinase (Gbem_1326) similar to those of *Streptomyces lividans *and *Streptomyces coelicolor *[32,33], a UDP-glucose/galactose 4-epimerase (Gbem_3215) and a different putative galactose-1-phosphate uridylyltransferase. *G. bemidjiensis *was able to grow with glucose, but not galactose, as electron donor and carbon source, using Fe(III) oxides as the terminal electron acceptor (D. Holmes, unpublished). This discovery illustrates the need for subsurface metabolic models to be based on subsurface genomes such as that of *G. bemidjiensis*, rather than approximations based on genomes of non-subsurface species.

### Carbohydrate osmoprotectants and cell wall components

Like *G. sulfurreducens *and *G. metallireducens*, *G. bemidjiensis *is predicted to make trehalose from glucose storage polymers by the sequential action of maltooligosyltrehalose synthase (Gbem_0134) and maltooligosyltrehalose trehalohydrolase (Gbem_0132) [10]. *G. bemidjiensis *lacks homologs of the enzymes predicted to make trehalose from glucose-6-phosphate in *G. sulfurreducens *[10], but may be able to isomerize maltose to trehalose by means of a maltose-active trehalose synthase (Gbem_0136) that is 33% identical to that of *T. thermophilus *[34]. The presence of a fructose/mannose 6-kinase (*mak *Gbem_0370), 39% identical to that of *E. coli *[35] and a mannitol dehydrogenase (Gbem_0401) with 47% identity to that of *Apium graveolens *[36] suggests that *G. bemidjiensis *may synthesize and break down D-mannitol as an additional osmoprotectant.

The lipopolysaccharide of *G. sulfurreducens *contains no O-antigen [37]. In contrast, the genome of *G. bemidjiensis *reveals many pathways for the production of various sugars that may be components of the cell wall (Additional file 3: Table S3). Thus, not only central metabolism of carbon but many specialized branch pathways appear to differ between subsurface and non-subsurface *Geobacter *species.

### Biosynthesis of chorismate and folate in *G. bemidjiensis*

**Figure 5:**
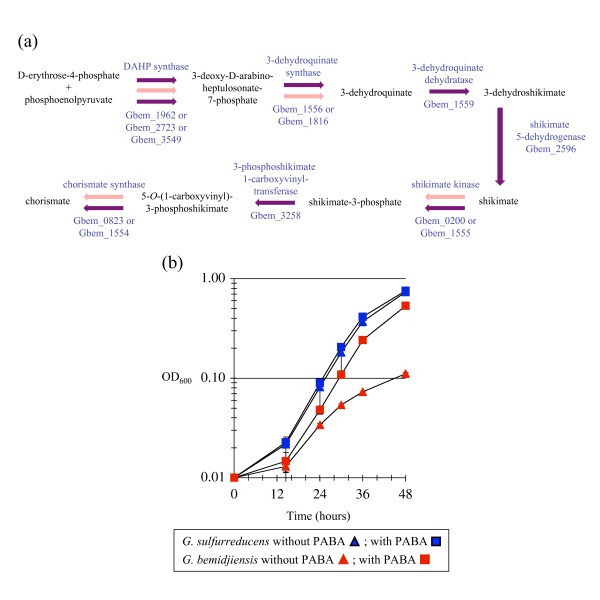
**Chorismate biosynthesis pathway and 4-aminobenzoate auxotrophy of *G. bemidjiensis***. **(a) **Multiplicity of enzymes of chorismate biosynthesis in *G. bemidjiensis*. Every step may be catalyzed by enzymes shared with *G. sulfurreducens *and *G. metallireducens *(purple arrows), but isozymes for some steps are unique to *G. bemidjiensis *(pink arrows). **(b) **Growth of *G. bemidjiensis *on NBAF medium on the first transfer without 4-aminobenzoate (PABA) supplementation (red triangles) compared to growth with PABA (red squares); the controls were *G. sulfurreducens *without PABA (blue triangles) and with PABA (blue squares).

Chorismate is the common precursor of folate, menaquinone, and the aromatic amino acids phenylalanine, tyrosine and tryptophan. *Geobacter *species have multiple isozymes for the first reaction of the chorismate biosynthesis pathway [10], which may respond to negative feedback from different end products as in *E. coli *[38]. The chorismate biosynthesis pathway in *G. bemidjiensis *and *Geobacter *sp. M21 is notable for the presence of isozymes to catalyze several subsequent reactions as well (Figure 5a), differing in this aspect from *G. sulfurreducens *and *G. metallireducens*. Scattered genes encode a 3-dehydroquinate synthase with homologs in *G. daltonii *and cyanobacteria, a shikimate kinase with homologs in *Marinobacter *species, methanogens and Clostridia, and a chorismate synthase with homologs in sulfate-reducers and fungi. There is also a cluster of three genes encoding the same functions, as in *G. sulfurreducens *and *G. metallireducens*. The presence of these isozymes at multiple steps suggests that regulation of chorismate biosynthesis may be more complex in subsurface *Geobacter *species.

The *G. bemidjiensis *genome encodes no homolog of the putative 4-aminodeoxychorismate synthase/lyase of *G. metallireducens *and *G. sulfurreducens *(Gmet_3010 = GSU0523), and an attempt to grow *G. bemidjiensis *without vitamin supplementation confirmed that it is auxotrophic solely for the 4-aminobenzoate (PABA) moiety of folate (Figure 5b). This is the first report of a *Geobacter *species with any vitamin requirement, and suggests that the metabolic activity of subsurface *Geobacter *species may be stimulated by adding PABA.

### Degradation of benzoate and other aromatic pollutants

**Figure 6:**
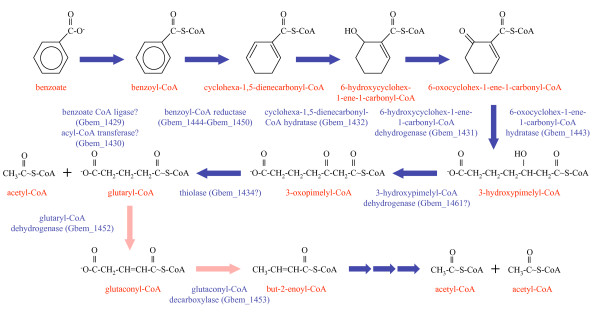
**Benzoate degradation pathway in *G. bemidjiensis***. Early enzymes have orthologs in *G. metallireducens *(blue arrows), but glutaryl-CoA dehydrogenase and glutaconyl-CoA decarboxylase are unique to *G. bemidjiensis *(pink arrows).

The ability to degrade benzoate is found in both subsurface and non-subsurface *Geobacter *species. *G. bemidjiensis *possesses orthologs of the *G. metallireducens *genes implicated in degradation of benzoate to 3-hydroxypimelyl-CoA (Figure 6) [39-42]. The benzoate--CoA ligase (*bamY *Gbem_1429) gene sequence contains an internal stop codon (TAG) at position 448, where those of other *Geobacter *species specify glutamate (GAG). However, the gene next to *bamY *(Gbem_1430) encodes an acyl-CoA hydrolase/transferase of unknown specificity. The possibility that benzoate can be activated to benzoyl-CoA by this enzyme, perhaps by transferring CoA from succinyl-CoA or acetyl-CoA, should be explored. Like *G. metallireducens*, *G. bemidjiensis *possesses *bamB-2 *and *bamC-2 *genes (Gbem_2620-Gbem_2619) paralogous to *bamB *and *bamC *of the putative benzoyl-CoA reductase, and a ferredoxin gene (Gbem_2621) closely related to the N-terminal domain of *bamB*.

Degradation of 3-hydroxypimelyl-CoA in *G. bemidjiensis *(Figure 6) is predicted to involve a non-decarboxylating glutaryl-CoA dehydrogenase (Gbem_1452) 44% identical to that of *Desulfococcus multivorans*, rather than a homolog of the decarboxylating glutaryl-CoA dehydrogenase of *G. metallireducens *[43]. Subsequent decarboxylation of glutaconyl-CoA may take place through the product of the adjacent gene (*gcdA *Gbem_1453), which is 52% identical to the catalytic subunit of sodium-translocating glutaconyl-CoA decarboxylase of *Acidaminococcus fermentans *[44]. No homologs of the other three subunits were found in the *G. bemidjiensis *genome, indicating that decarboxylation of glutaconyl-CoA is not coupled to a sodium pump. Although the GcdA protein of *A. fermentans *on its own is capable of decarboxylating glutaconyl-CoA with free biotin as a cofactor [45], it is notable that the oxaloacetate decarboxylase encoded by the gene adjacent to *gcdA *in *G. bemidjiensis *(*oadA *Gbem_1454) contains two biotin attachment domains, whereas its sodium pump-associated homologs contain only one. The possibility that the two decarboxylases cooperate as a complex deserves investigation.

Although the genome of *G. bemidjiensis *corroborates the observation that it cannot degrade as many aromatic compounds as *G. metallireducens *[39,42,46], it also suggests that *G. bemidjiensis *can detoxify some aromatic pollutants without degrading them. A homolog of the broad-specificity aldo-keto reductase YvgN of *B. subtilis *[47] is present (Gbem_3980, 45% sequence identity), suggesting that *G. bemidjiensis *may convert chloro- and nitro- derivatives of benzaldehyde to the corresponding benzol derivatives. Although YvgN was previously described as a methylglyoxal reductase [48], detoxification of methylglyoxal, a byproduct of carbohydrate and lipid metabolism, may be of minor importance in *G. bemidjiensis*, as a methylglyoxal synthase was not found in *G. bemidjiensis*, but only in *G. lovleyi *(Glov_0611).

The fact that *G. bemidjiensis *is auxotrophic for PABA (Figure 5b) indicates that it has grown accustomed to an environment in which PABA is readily available, possibly in the form of 4-azobenzoate. *G. bemidjiensis *may convert azoaromatic compounds to arylamines by means of an azoreductase (*azoR *Gbem_2529) 30% identical to that of *E. coli *[49], which is not present in *G. metallireducens*. Furthermore, an arylamine *N*-acetyltransferase (Gbem_0306) not found in other *Geobacteraceae *may act in detoxification of aromatic compounds by *G. bemidjiensis*, and a putative amidohydrolase in the benzoate degradation gene cluster (Gbem_1458 = Gmet_2056) may also be involved in metabolism of aromatic compounds. Further studies of subsurface *Geobacter *species such as *G. bemidjiensis *are necessary to characterize their abilities to transform aromatic compounds.

### Alkylmercury lyase

The presence of an alkylmercury lyase (Gbem_0319) 36% identical to that of *S. lividans *[50]) suggests that *G. bemidjiensis *possesses broad-spectrum resistance to mercury in various organic forms. A homolog of this enzyme was not found in any other *Geobacter *species, but genes that may encode mercuric reductases (e.g. Gbem_0457, Gbem_0640) exhibit vertical inheritance in the family, indicating that *Geobacteraceae *may generally have the ability to detoxify inorganic Hg(II) ions, whereas subsurface *Geobacter *species such as *G. bemidjiensis *may have acquired additional resistance to organomercuric compounds.

### Expansion of transport systems for phosphate and molybdate

**Figure 7:**
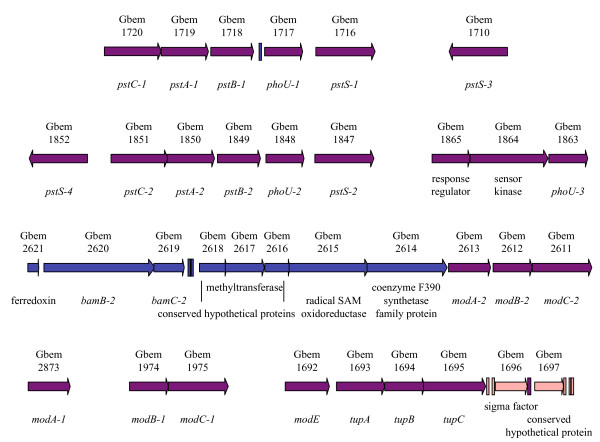
**Multiplicity of phosphate and molybdate transport system genes of *G. bemidjiensis***. Some genes are unique to *G. bemidjiensis *(pink arrows), some have orthologs in *G. metallireducens *(blue arrows), and some are shared with both *G. sulfurreducens *and *G. metallireducens *(purple arrows). Multicopy nucleotide sequences (rectangles) are found in some intergenic regions. The periplasmic phosphate-binding proteins are encoded by *pstS *genes, the transporter membrane proteins by *pstA *and *pstC *genes, ATP-binding proteins by *pstB *genes, and regulators by *phoU *genes. The periplasmic molybdate-binding proteins are encoded by *modA *genes, the transporter membrane proteins by *modB *genes, the ATP-binding proteins by *modC *genes, and a regulator by *modE*. The periplasmic tungstate-binding protein is encoded by *tupA*, the transporter membrane protein by *tupB*, and the ATP-binding protein by *tupC*. The *bamB-2 *gene encodes a paralog of benzoyl-CoA reductase, and the *bamC-2 *gene encodes a paralog of a putative electron transfer protein of benzoyl-CoA reductase.

The ATP-binding cassette (ABC) transport system for phosphate consists of a periplasmic phosphate-binding protein (PstS), two membrane proteins (PstC and PstA), an ATP-binding protein (PstB) and a regulatory protein (PhoU). Whereas these are encoded by single-copy genes in *G. sulfurreducens *and *G. metallireducens*, the *G. bemidjiensis *genome contains duplicate transporters, triplicate regulators, and quadruplicate phosphate-binding proteins (Figure 7). This expansion may confer an advantage in the subsurface environment, where *Geobacter *species experience phosphate limitation [51].

The ABC transport system for molybdate, consisting of a molybdate-binding protein (ModA), membrane protein (ModB) and ATP-binding protein (ModC), has also expanded in *G. bemidjiensis *(Figure 7): the *modB_1_C_1 _*genes are located apart from the *modA_1 _*gene, while the *modA_2_B_2_C_2 _*genes remain an intact operon. The regulatory gene *modE *is located on the 5' side of the tungstate transporter genes *tupABC *(which are phylogenetically distinct from those of *G. sulfurreducens *and *G. metallireducens*; data not shown) in contrast with its location on the 5' side of *modABC *in *G. sulfurreducens*. The possibility that these expansions and rearrangements are a response by subsurface *Geobacter *species to molybdate limitation deserves to be investigated.

### Oxygen respiration, oxygen detoxification, and possible anaerotaxis in *G. bemidjiensis*

**Figure 8:**
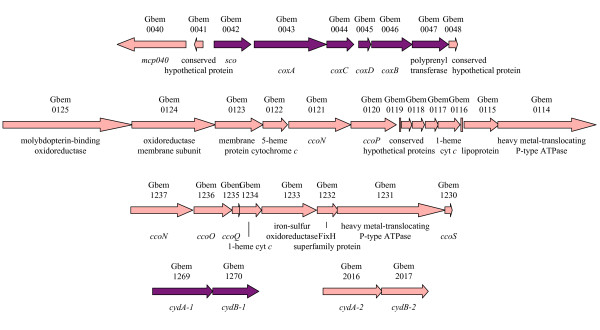
**Oxygen respiration genes of *G. bemidjiensis***. The cytochrome *caa_3 _*oxidase complex (*coxACDB *genes) and one cytochrome *bd *quinol oxidase complex (*cydAB-1 *genes) are encoded by genes with orthologs in *G. sulfurreducens *and *G. metallireducens *(purple arrows), while the hemerythrin domain-containing methyl-accepting chemotaxis sensory transducer (*mcp040*), the other pair of cytochrome *bd *quinol oxidase complex genes (*cydAB-2*), and the two operons encoding cytochrome *bb_3 _*oxidase components (*cco *genes) are unique to *G. bemidjiensis *(pink arrows).

Genome sequencing led to the surprising discovery that *G. sulfurreducens *and *G. metallireducens *are capable of oxygen respiration using a cytochrome *caa_3 _*oxidase complex, which is also found in *G. bemidjiensis *(Gbem_0042-Gbem_0047). Near this operon in *G. bemidjiensis *only (Figure 8) is a gene encoding a methyl-accepting chemotaxis protein (Gbem_0040) with a hemerythrin-like domain, possibly to sense oxygen, by analogy with the much larger DcrH protein of *D. vulgaris *[52]. In addition, *G. bemidjiensis *has two other operons encoding components of cytochrome *cbb_3 _*oxidases: (Gbem_1237-Gbem_1230; Gbem_0121-Gbem_0120). The *G. bemidjiensis *genome contains two pairs of genes encoding cytochrome *bd *quinol oxidases: one (Gbem_1269-Gbem_1270) is closely related to its counterparts in *G. sulfurreducens *and *G. metallireducens*, while the other (Gbem_2016-Gbem_2017) is not. Thus, *G. bemidjiensis *may have enhanced capabilities for oxygen respiration compared to *G. sulfurreducens *and *G. metallireducens*, due to oxygen stress in its subsurface niche, and may have the ability to respond to oxygen anaerotactically through chemotaxis-type signalling.

Eight hemerythrin family proteins (Gbem_1252, Gbem_2241, Gbem_2255, Gbem_2262, Gbem_2701, Gbem_2773, Gbem_3870, Gbem_4009) were predicted from the genome of *G. bemidjiensis*, suggesting that it may have expanded its ability to sequester molecular oxygen and deliver it to respiratory or detoxifying enzymes, in contrast to *G. metallireducens *with two hemerythrin homologs; *G. sulfurreducens *has six hemerythrin homologs. To detoxify reactive oxygen species, *G. bemidjiensis *possesses a desulfoferrodoxin (Gbem_3292) 60% identical to that of *Desulfoarculus baarsii *[53] and a rubredoxin:oxygen/nitric oxide oxidoreductase (Gbem_0186) 31% identical to that of *D. gigas *[54], in addition to the superoxide dismutase (Gbem_2204), peroxiredoxins (Gbem_0154, Gbem_0221, Gbem_1338, Gbem_2956, Gbem_4010) and two rubrerythrins (Gbem_2313, Gbem_3325) also present in *G. sulfurreducens *and *G. metallireducens*. Phylogenetic analysis (not shown) indicates that although the characterized cytochrome *c *peroxidase of *G. sulfurreducens *[55] has an excellent homolog in *G. bemidjiensis *(Gbem_0020), this is actually an ortholog of MacA, implicated in Fe(III) reduction [56]. As in *G. metallireducens*, there is no catalase in *G. bemidjiensis*, meaning that no oxygen is produced from detoxification of hydrogen peroxide; detoxification by rubrerythrins produces only water. All *Geobacteraceae *encode at least one iron-sulfur-oxygen hybrid cluster protein, thought to detoxify an unidentified reactive compound in response to nitric oxide stress [57], as well as hydrogen peroxide stress [58]; *G. bemidjiensis *and *Geobacter *sp. M21 alone have three hybrid cluster protein genes (Gbem_1033, Gbem_1168, Gbem_1239), evidently derived by expansion of a single ancestral gene. *G. bemidjiensis *also has a quinol-oxidizing nitric oxide reductase (*norZ *Gbem_3901) 40% identical to that of *Cupriavidus necator *[59], with a distant homolog in *G. metallireducens*. Overall, the genome annotation indicates that *G. bemidjiensis *has evolved to cope with many kinds of reactive oxygen species, a finding that should improve models of *Geobacter *metabolism in the subsurface.

### The outer surface: *c*-type cytochromes, pili, and flagella

**Figure 9:**
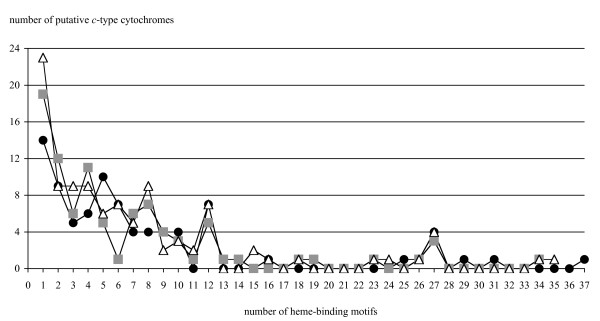
**Distribution of *c*-type cytochromes with different numbers of heme-binding motifs in three *Geobacter *genomes**. The pattern for *G. bemidjiensis *(black circles) shows some differences from *G. sulfurreducens *(white triangles) and *G. metallireducens *(grey squares).

An understanding of subsurface *Geobacter *metabolism and physiology must include the *c*-type cytochromes that mediate electron transfer to extracellular electron acceptors such as insoluble Fe(III) oxides. The number of genes encoding *c*-type cytochromes in the *G. bemidjiensis *genome is 84, fewer than *G. metallireducens *(90 genes) and *G. sulfurreducens *(104 genes), despite the *G. bemidjiensis *genome being the largest of the three. The distribution of genes with a given number of heme-binding motifs (Figure 9) shows that all three species encode multiple *c*-type cytochromes with 12 or 27 predicted hemes (due to fusion of four or nine modules of the cytochrome *c_7 _*family, respectively), and the most complex *c*-type cytochrome in each genome contains 34-37 hemes. However, *G. sulfurreducens *and *G. metallireducens *encode more monoheme *c*-type cytochromes than *G. bemidjiensis*, and while the mode number of hemes in multiheme *c*-type cytochromes of *G. bemidjiensis *is 5 hemes, the other two genomes have two modes with 4 and 8 hemes. Of the *c*-type cytochrome genes implicated in Fe(III) and U(VI) reduction in *G. sulfurreducens*, *G. bemidjiensis *possesses *macA *(Gbem_0020) [56,60,61], three members of the *c_7 _*family (*ppcB *Gbem_4049, *ppcD *Gbem_4043, and *ppcG *Gbem_3455) [62], five members of the *omcS *family (Gbem_1116, Gbem_1117, Gbem_1131, Gbem_2679, Gbem_2680) [63], one member of the *omcB *family (Gbem_3379) [64], and two *omcF*-related genes (Gbem_1585, Gbem_2183) [65]. There is no homolog of *omcE *[63]. Although *G. bemidjiensis *was reported not to grow with a graphite electrode as electron acceptor [3], it possesses a homolog of *omcZ *(Gbem_3056), which is required for *G. sulfurreducens *to grow by transferring electrons to an electrode [66]. Overall, 41 *c*-type cytochromes of *G. bemidjiensis *(49%) have full-length homologs in *G. sulfurreducens *and/or *G. metallireducens *(Additional file 4: Table S4). Larger *c*-type cytochromes (13 or more heme-binding motifs) of *G. bemidjiensis *are absent from *G. sulfurreducens *and *G. metallireducens *with one exception (Gbem_1124 = GSU2495; 26 heme-binding motifs). Of the *c*-type cytochromes of *G. bemidjiensis *that have one, two or five heme-binding motifs, approximately two-thirds in each category have no match in the other two genomes, whereas of those with three, four or six to twelve heme-binding motifs, at least half in each category are shared. Thus, extensive similarities as well as notable differences in the complement of *c*-type cytochromes exist between the subsurface species *G. bemidjiensis *and its non-subsurface relatives.

**Figure 10:**
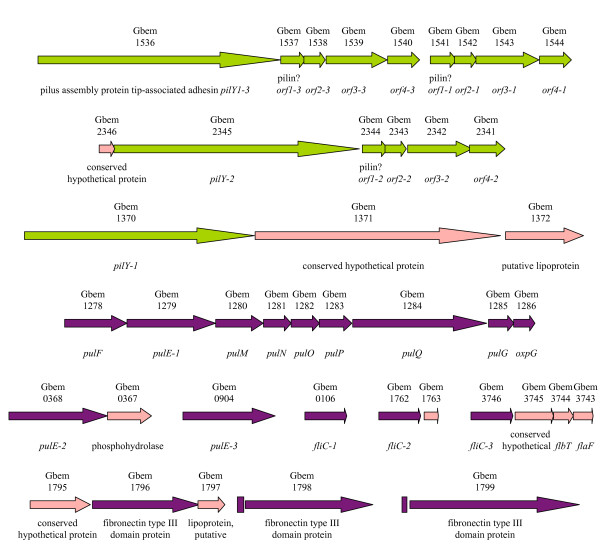
**Triplicated genes for biogenesis of pili and flagella in *G. bemidjiensis***. The pilus assembly protein tip-associated adhesin genes (*pilY1*) and associated genes (*orf1*, *orf2*, *orf3*, *orf4*) have single orthologs in *G. sulfurreducens *(green arrows); those of *G. metallireducens *are of different origin. The flagellin genes (*fliC*) and pilus biogenesis ATPase genes (*pulE*) have single orthologs in both *G. sulfurreducens *and *G. metallireducens *(purple arrows), while genes predicted to be in the same operons are often unique to *G. bemidjiensis *(pink arrows). GEMM riboswitches (purple rectangles) are found near the recently triplicated fibronectin type III domain protein genes.

Several genes of pilus biogenesis are present in triplicate in the *G. bemidjiensis *genome (Figure 10), all apparently expanded from ancestral single-copy genes found in *G. sulfurreducens *and other *Geobacteraceae *(but not *G. metallireducens*). The ancestral flagellin gene has also expanded into triplicates (Gbem_0106, Gbem_1762, Gbem_3746), as has an ancestral GEMM riboswitch-regulated gene encoding a fibronectin type III domain protein (Gbem_1796, Gbem_1798, Gbem_1799) that may be localized to the outer surface of the cell. Interestingly, phylogenetic analysis (not shown) indicates that *G. bemidjiensis *has also recently triplicated (as Gbem_1116, Gbem_2659 and Gbem_2680) an ancestral gene related to *omcS *of *G. sulfurreducens*, which encodes an outer surface *c*-type cytochrome that is important for reduction of insoluble Fe(III) [63]. Thus, many proteins of the outer surface, with possible roles in electron transfer to insoluble extracellular electron acceptors, are triply present in *G. bemidjiensis*, although the significance of this expansion to life in subsurface environments is unknown.

### Sigma factors and signalling proteins

The *G. bemidjiensis *genome was examined for features of gene regulation conserved between it and its non-subsurface relatives. Of the six sigma factors of RNA polymerase in *G. sulfurreducens*, *G. bemidjiensis *has orthologs of five: RpoD (Gbem_3694), RpoS (Gbem_2683), RpoN (Gbem_0869), RpoH (Gbem_0573), and FliA (Gbem_3764). No homolog of the putative stress response sigma factor RpoE was found. There are also two additional sigma factors (Gbem_1696 and Gbem_3169) unrelated to the unique sigma factor of *G. metallireducens*.

The *G. bemidjiensis *genome encodes 127 putative sensor histidine kinases containing HATPase_c domains (Additional file 5: Table S5), including 8 chemotaxis-type kinases (*cheA *genes), of which 47 genes (37%) have full-length homologs in *G. sulfurreducens *and/or *G. metallireducens*. There are 163 proteins with response receiver (REC) domains (Additional file 5: Table S5), including 19 that may belong to chemotaxis-type signalling pathways; of these, 82 genes (50%) have full-length homologs in *G. sulfurreducens *and/or *G. metallireducens*. Thus, *G. bemidjiensis *has a different and much larger repertoire of phosphorylation-dependent signalling proteins than either *G. sulfurreducens *or *G. metallireducens*. The *G. bemidjiensis *genome encodes 21 GGDEF domain proteins that may synthesize the intracellular messenger cyclic diguanylate (Additional file 5: Table S5), a similar number to *G. sulfurreducens *and *G. metallireducens*, but only 10 of these are conserved. These differences in the repertoire of predicted signalling proteins among subsurface and non-subsurface *Geobacter *species are remarkable, especially considering that some ancestral genes encoding signalling proteins appear to have undergone duplication or triplication in *G. bemidjiensis *(Additional file 5: Table S5).

### Non-protein-coding features of the *G. bemidjiensis *genome

Riboswitches that have been identified in the non-subsurface species *G. sulfurreducens *and *G. metallireducens *[10,67] were found in the subsurface *G. bemidjiensis *genome also. In addition, several families of multicopy nucleotide sequences were noted in *G. bemidjiensis *(Additional file 6: Table S6; Additional files 7, 8, 9, 10, 11, 12, 13, 14 and 15: Figures S1-S9), most of which have no counterparts in the *G. sulfurreducens *or *G. metallireducens *genomes. Some of these families are based on palindromic sequences, and others consist of direct repeats of 6 to 42 nucleotides that occupy intergenic regions throughout the genome. Multicopy sequences (other than rRNA and tRNA genes) are found in 12% of regions between protein-coding genes in *G. bemidjiensis*, and multiple sequences are present in 31% of such intergenic regions, indicating that insertion is not random. One nucleotide sequence family was found inserted into protein-coding genes (on both strands and in all reading frames, without causing frameshifts) as well as between genes, as previously observed for a different family in *G. metallireducens *[10]. The implications of so many more multicopy sequences being present in the genome of a subsurface *Geobacter *species than in those of its non-subsurface relatives remain to be elucidated.

## Conclusions

The complete genome sequence of *G. bemidjiensis *reveals many differences from the previously published genomes of non-subsurface *Geobacter *species. Enzymes that account for the metabolic versatility of *G. bemidjiensis *were identified, and further metabolic, physiological and genomic peculiarities were discovered, including a more efficient pathway for oxidation of propionate, a pathway of fumarate disproportionation without excretion of acetate, a reductive TCA cycle, utilization of glucose, a defective folate biosynthesis pathway, and enhanced abilities to respond to oxygen stress. This information is of utmost value for an understanding of the activity of *Geobacteraceae *in subsurface environments undergoing bioremediation accompanied by reduction of Fe(III).

## Methods

### Sequence analysis and annotation

The genome of *G. bemidjiensis *Bem(T) [3] was sequenced at the Joint Genome Institute (JGI) using a combination of 3 kb, 6 kb and 35 kb DNA libraries. Inserts were sequenced from both ends using the standard Sanger method. All three libraries provided 11-fold coverage of the genome. The Phred/Phrap/Consed software package http://www.phrap.com was used for sequence assembly and quality assessment [68-70]. After the shotgun stage, 65888 reads were assembled with parallel Phrap (High Performance Software, LLC). Possible mis-assemblies were corrected with Dupfinisher (Han, 2006) or transposon bombing of bridging clones (Epicentre Biotechnologies, Madison, WI). Gaps between contigs were closed by editing in Consed, by custom primer walks, or by PCR amplification (Roche Applied Science, Indianapolis, IN). A total of 2059 additional reactions were necessary to close gaps and to raise the quality of the finished sequence. The completed genome sequence of *G. bemidjiensis *Bem(T) contains 67990 reads, achieving an average of 11-fold sequence coverage per base with an error rate less than 1 in 100,000.

The protein-coding genes were predicted using Prodigal V1.0 [71]. A BLASTP search of the translations *vs*. Genbank's non-redundant database (NR of Nov. 2007) at 1e-05 evalue was conducted. Matches to the *Geobacter *genus were excluded and the alignment of the N-terminus of each gene model *vs*. the best NR match was used to pick a preferred gene model. The gene/protein set was searched using BLASTP, hmmer, RPS-BLAST and Interpro. BLASTP searches were done *vs*. Swiss-Prot/TrEMBL, NR, and KEGG databases with a cutoff evalue of 1e-05. Hmmer searches were done *vs*. Pfam and TIGRfam databases using the trusted cutoff. RPS-BLAST searches against PRIAM used its 1e-30 high-confidence cutoff and searches against COGS used a 1e-10 cutoff. Interpro was run using its default cutoffs. Automated product assignment was made using the following hierarchy of data sources: PRIAM, TIGRFam, Pfam, Interpro profiles, Swiss-Prot/TrEMBL, KEGG, and COG group. tRNAs were annotated using tRNAscan-SE (v1.23). rRNAs were annotated using RNAmmer v 1.1 [72]. The srpRNA was located using the SRPscan website. The *rnpB *and *ssrA *genes were located using the Rfam database and Infernal.

### Manual curation

The automated genome annotation of *G. bemidjiensis *and the manually curated genome annotations of *G. sulfurreducens *and *G. metallireducens *[10] were queried reciprocally with the protein BLAST algorithm [73] as implemented by OrthoMCL [74] using the default inflation parameter value (1.5), to identify mutual best hits as potential orthologs. The functional annotations of *G. bemidjiensis *genes were emended for consistency with their counterparts in *G. sulfurreducens *and *G. metallireducens*. The coordinates of numerous genes were adjusted according to the criteria of full-length alignment, plausible ribosome-binding sites, and minimal overlap between genes on opposite DNA strands. The annotations of *G. bemidjiensis *genes that were not matched to genes in *G. sulfurreducens *or *G. metallireducens *were checked by BLAST searches of NR and the Swiss-Prot database. Functional annotations in all three genomes were updated to match the experimental characterization of highly similar full-length homologs, with extensive reference to the EcoSal online textbook http://www.ecosal.org and the MetaCyc database [75]. Genes that had no protein-level homologs in NR were checked (together with flanking intergenic sequences) by translated nucleotide BLAST in all six reading frames, and by nucleotide BLAST to ensure that conserved protein-coding or non-protein-coding features had not been missed. All intergenic regions of 30 bp or larger were also checked, which led to the annotation of numerous conserved nucleotide sequences.

### Phylogenetic analysis

Phylogenetic analysis of selected proteins was performed. In each case, the protein sequence of interest was included, along with its relatives, as identified by BLAST [76], and the set of sequences was aligned by TCoffee [77]. ProtTest [78] was used to select a model of molecular evolution and MrBayes [79] was used to create a Bayesian estimation of the phylogeny.

### Growth experiments

To monitor the disproportionation of fumarate, *G. bemidjiensis *was cultured under strictly anaerobic conditions at 30°C in an atmosphere of N_2 _and CO_2 _(80%:20%), as previously described for *G. sulfurreducens *[80], in rubber-stoppered 156 ml bottles containing NBAF medium [81] from which sodium acetate was omitted. Samples of 1 ml were removed aseptically using anoxic syringes to monitor growth, then diluted 50-fold, passed through a 0.22 μm filter to remove cells, and stored at 4°C until high-pressure liquid chromatography analysis was performed as described previously [19]. The 4-aminobenzoate requirement of *G. bemidjiensis *was tested in rubber-stoppered 26 ml glass tubes containing 10 ml of NBAF medium [81] from which the vitamin solution and resazurin were omitted, with 4-aminobenzoate (Sigma Aldrich) added to individual tubes to a final concentration of 100 μg/L.

## List of Abbreviations used

ABC: ATP-binding cassette; ATP: adenosine 5'-triphosphate; CoA: coenzyme A; DNA: deoxyribonucleic acid; FAD: flavin adenine dinucleotide; GTP: guanosine 5'-triphosphate; NAD(H): nicotinamide adenine dinucleotide (reduced); NADP(H): nicotinamide adenine dinucleotide 2'-phosphate (reduced); PABA: 4-aminobenzoate; rRNA: ribosomal RNA; RNA: ribonucleic acid; TCA: tricarboxylic acid; tRNA: transfer RNA; UDP: uridine 5'-diphosphate.

## Authors' contributions

CH supervised the genome sequencing, HK performed genome sequence finishing, and ML oversaw the automated annotation process. MA performed manual curation of the genome annotation (assisted by MC and NY) and wrote the manuscript. NY did the phylogenetic analyses. DH, CR and MA conducted physiological experiments. DL conceived of the study and offered guidance with the writing. All authors read, assisted with editing, and approved the final manuscript.

## Supplementary Material

Additional file 1 **Table S1**. Genes of *G. bemidjiensis *in which frameshifts occur in homopolymeric regions. Click here for file

Additional file 2 **Table S2**. Enzymes of acyl-CoA metabolism in *G. bemidjiensis*. Click here for file

Additional file 3 **Table S3**. Selected sugar interconversion genes of *G. bemidjiensis*. Click here for file

Additional file 4 **Table S4**. Predicted *c*-type cytochromes of *G. bemidjiensis*. Click here for file

Additional file 5 **Table S5**. Signalling proteins of *G. bemidjiensis*. Click here for file

Additional file 6 **Table S6**. Multicopy nucleotide sequences in *G. bemidjiensis*. Click here for file

Additional file 7 **Figure S1**. Multicopy nucleotide sequences of the *G. bemidjiensis *genome: base coordinates and alignments. (See also Table S6.). Click here for file

Additional file 8 **Figure S2**. Multicopy nucleotide sequences of the *G. bemidjiensis *genome: base coordinates and alignments. (See also Table S6.). Click here for file

Additional file 9 **Figure S3**. Multicopy nucleotide sequences of the *G. bemidjiensis *genome: base coordinates and alignments. (See also Table S6.). Click here for file

Additional file 10 **Figure S4**. Multicopy nucleotide sequences of the *G. bemidjiensis *genome: base coordinates and alignments. (See also Table S6.). Click here for file

Additional file 11 **Figure S5**. Multicopy nucleotide sequences of the *G. bemidjiensis *genome: base coordinates and alignments. (See also Table S6.). Click here for file

Additional file 12 **Figure S6**. Multicopy nucleotide sequences of the *G. bemidjiensis *genome: base coordinates and alignments. (See also Table S6.). Click here for file

Additional file 13 **Figure S7**. Multicopy nucleotide sequences of the *G. bemidjiensis *genome: base coordinates and alignments. (See also Table S6.). Click here for file

Additional file 14 **Figure S8**. Multicopy nucleotide sequences of the *G. bemidjiensis *genome: base coordinates and alignments. (See also Table S6.). Click here for file

Additional file 15 **Figure S9**. Multicopy nucleotide sequences of the *G. bemidjiensis *genome: base coordinates and alignments. (See also Table S6.). Click here for file
