# Supplementary material for: The genome of Geobacter bemidjiensis, exemplar for the subsurface clade of Geobacter species that predominate in Fe(III)-reducing subsurface environments
Source: BMC Genomics. 2010 Sep 9;11:490. doi: 10.1186/1471-2164-11-490 (PMC2996986; doi:10.1186/1471-2164-11-490)
Supplement: Additional file 1 — Table S1. Genes of G. bemidjiensis in which frameshifts occur in homopolymeric regions. [file 1471-2164-11-490-S1.PDF]

Table S1. Genes of *G. bemidjiensis* in which frameshifts occur in homopolymeric regions.

Gene	Annotation	Frameshift coordinates	Frameshifted and unframeshifted sequences (number of reads)
Gbem_0007	conserved hypothetical protein	9836 to 9843	CCCCCCCC (10) to CCCCCCCCC (5)
Gbem_0093	radical SAM domain iron-sulfur cluster-binding oxidoreductase	117347 to 117353	GGGGGGG (5) to GGGGGG (4)
Gbem_0298	TPR domain protein	364592 to 364598	GGGGGGG (6) to GGGGGGGG (4)
Gbem_0370	*mak*; D-fructose 6-kinase	449242 to 449246	GGGGG (10) to GGGGGG (0)
Gbem_0469	sensor histidine kinase response regulator (GAF, HisKA, HATPase_c, REC)	565796 to 565802	CCCCCCC (13) to CCCCCC (0)
Gbem_0559	methylmalonyl-CoA mutase, isobutyryl-CoA mutase-like catalytic subunit	673326 to 673333	GGGGGGGG (4) to GGGGGGG (0)
Gbem_0706	*lpdA-1*; 2-oxoglutarate dehydrogenase complex, E3 protein, dihydrolipoamide dehydrogenase	837161 to 837169	GGGGGGGGG (11) to GGGGGGGG (0)
Gbem_0875	*ptsI*; phosphoenolpyruvate-protein phosphotransferase	1029608 to 1029613	GGGGGG (7) to GGGGGGG (6)
Gbem_1051	conserved hypothetical protein	1224257 to 1224263	GGGGGGG (10) to GGGGGGGG (0)
Gbem_1088	cytochrome b/b_6 complex, iron-sulfur subunit	1263849 to 1263855	CCCCCCC (12) to CCCCCCCC (0)
Gbem_1120	conserved hypothetical protein	1294876 to 1294883	GGGGGGGG (16) to GGGGGGGGG (1)
Gbem_1145	oxalate/formate antiporter, putative	1322945 to 1322952	CCCCCCCC (12) to CCCCCCC (0)
Gbem_1157	cytochrome *c*, 12 heme-binding sites	1345533 to 1345538	GGGGGG (16) to GGGGGGG (1)
Gbem_1370	*pilY1-1*; Tfp pilus assembly protein tip-associated adhesin PilY1-like protein	1578791 to 1578798	GGGGGGG (12) to GGGGGG (0)
Gbem_1782	conserved hypothetical protein	2057644 to 2057652	GGGGGGGGG (11) to GGGGGGGG (0)
Gbem_1922	ATS1 domain repeat protein	2224595 to 2224602	GGGGGGGG (6) to GGGGGGG (0)

Gbem_2386	sensor histidine kinase (HATPase_c)	2766454 to 2766459	CCCCCC (8) to CCCCCCC (2)
Gbem_2429	*recJ*; single-stranded-DNA-specific exonuclease RecJ	2817844 to 2817850	CCCCCCC (6) to CCCCCC (0)
Gbem_2543	conserved hypothetical protein	2947105 to 2947112	GGGGGGGG (19) to GGGGGGGGG (0)
Gbem_2614	coenzyme F390 synthetase superfamily protein	3032323 to 3032329	CCCCCCC (13) to CCCCCC (3)
Gbem_3201	*era*; GTP-binding protein Era	3692199 to 3692204	CCCCCC (7) to CCCCCCC (0)
Gbem_3233	*fusA-2*; translation elongation factor G	3724008 to 3724016	CCCCCCCCC (9) to CCCCCCCCCC (0)
Gbem_3247	type I restriction/modification system N-6 DNA methylase	3739275 to 3739281	AAAAAAA (11) to AAAAAAAA (4)
Gbem_3295	sensor histidine kinase (HAMP, HisKA, HATPase_c)	3788413 to 3788419	CCCCCCC (13) to CCCCCCCC (0)
Gbem_3405	sensor histidine kinase (GAF, HisKA, HATPase_c)	3921208 to 3921214	CCCCCCC (7) to CCCCCCCC (0)
Gbem_3530	cardiolipin synthetase	4043251 to 4043259	CCCCCCCCC (10) to CCCCCCC (0)
Gbem_4015	glycoside hydrolase, family 2	4559577 to 4559584	CCCCCCCCC (10) to CCCCCCCC (0)
