# Supplementary material for: The genome of Geobacter bemidjiensis, exemplar for the subsurface clade of Geobacter species that predominate in Fe(III)-reducing subsurface environments
Source: BMC Genomics. 2010 Sep 9;11:490. doi: 10.1186/1471-2164-11-490 (PMC2996986; doi:10.1186/1471-2164-11-490)
Supplement: Additional file 2 — Table S2. Enzymes of acyl-CoA metabolism in G. bemidjiensis. [file 1471-2164-11-490-S2.PDF]

Table S2. Enzymes of acyl-CoA metabolism in *G. bemidjiensis*.

Gene	Name	Functional annotation
Gbem_2276	*pta*	phosphotransacetylase
Gbem_3897	*ato-4*	succinyl:acetate CoA transferase
Gbem_0468	*ato-1*	succinyl:acetate CoA transferase
Gbem_0795	*ato-3*	succinyl:acetate CoA transferase
Gbem_2843	*ato-2*	succinyl:acetate CoA transferase
Gbem_1430		acyl-CoA:carboxylate CoA transferase, putative
Gbem_1439		acyl-CoA:carboxylate CoA transferase, putative
Gbem_3573		acyl-CoA:carboxylate CoA transferase, putative
Gbem_1418		butyryl:4-hydroxybutyrate CoA transferase
Gbem_2825-Gbem_2824		coenzyme A transferase
Gbem_2827		coenzyme A transferase, family III
Gbem_0542		AMP-forming acyl-CoA synthetase
Gbem_1082		AMP-forming acyl-CoA synthetase
Gbem_1650		AMP-forming acyl-CoA synthetase
Gbem_2125		AMP-forming acyl-CoA synthetase
Gbem_2020		acyl-CoA synthetase
Gbem_3738		acyl-CoA synthetase
Gbem_0260-Gbem_0259	*sucCD-1*	succinyl-CoA synthetase
Gbem_1464-Gbem_1465	*sucCD-2*	succinyl-CoA synthetase
Gbem_3223-Gbem_3222	*sucCD-3*	succinyl-CoA synthetase
Gbem_1429	*bamY*	benzoate--CoA ligase
Gbem_0543		short-chain acyl-CoA dehydrogenase
Gbem_2827		short-chain acyl-CoA dehydrogenase
Gbem_2832		short-chain acyl-CoA dehydrogenase
Gbem_3575		short-chain acyl-CoA dehydrogenase
Gbem_3576		short-chain acyl-CoA dehydrogenase
Gbem_2128	*fadE*	very-long-chain acyl-CoA dehydrogenase
Gbem_1333-Gbem_1334	*etfBA-5*	electron transfer flavoprotein
Gbem_1357-Gbem_1358	*etfBA-1*	electron transfer flavoprotein
Gbem_1359		iron-sulfur cluster-binding oxidoreductase lipoprotein
Gbem_1463		iron-sulfur cluster-binding oxidoreductase
Gbem_1466-Gbem_1467	*etfBA-2*	electron transfer flavoprotein
Gbem_1468		iron-sulfur cluster-binding oxidoreductase
Gbem_1469-Gbem_1470	*bamOP/ etfBA-4*	electron transfer flavoprotein
Gbem_1500-Gbem_1501	*etfBA-3*	electron transfer flavoprotein
Gbem_1502		iron-sulfur cluster-binding oxidoreductase
Gbem_2085-Gbem_2086	*etfBA-6*	electron transfer flavoprotein
Gbem_2087		iron-sulfur cluster-binding oxidoreductase
Gbem_2830-Gbem_2829	*etfBA-7*	electron transfer flavoprotein

Gbem_2831		iron-sulfur cluster-binding oxidoreductase lipoprotein
Gbem_3229		iron-sulfur cluster-binding oxidoreductase lipoprotein
Gbem_1444-Gbem_1451	*$bamB_1C_1$ DFGHI*	putative benzoyl-CoA reductase and electron transfer proteins
Gbem_2621-Gbem_2619	*$bamB_2C_2$*	putative benzoyl-CoA reductase and electron transfer proteins
Gbem_1452		glutaryl-CoA dehydrogenase, non-decarboxylating
Gbem_1431	*bamQ*	6-hydroxycyclohex-1-ene-1-carbonyl-CoA dehydrogenase
Gbem_1443	*bamA*	6-oxocyclohex-1-ene-1-carbonyl-CoA hydratase
Gbem_1432	*bamR*	cyclohexa-1,5-dienecarbonyl-CoA hydratase
Gbem_0393-Gbem_0392		(R)-2-hydroxyglutaryl-CoA dehydratase D component-related protein and activator
Gbem_2039-Gbem_2040	*yjiLM*	(R)-2-hydroxyglutaryl-CoA dehydratase D component-related protein and activator
Gbem_1416		enoyl-CoA hydratase/isomerase
Gbem_1457		enoyl-CoA hydratase/isomerase
Gbem_1462		enoyl-CoA hydratase/isomerase
Gbem_2833		enoyl-CoA hydratase/isomerase
Gbem_1413		hydroxyacyl/enoyl-CoA dehydratase/reductase, putative
Gbem_2126		enoyl-CoA hydratase and 3-hydroxyacyl-CoA dehydrogenase, NAD-binding
Gbem_1417		3-hydroxyacyl-CoA dehydrogenase, NAD-binding
Gbem_1461		3-hydroxyacyl-CoA dehydrogenase
Gbem_2834		3-hydroxyacyl-CoA dehydrogenase
Gbem_1415		thiolase
Gbem_1434		thiolase
Gbem_1460	*bamN*	thiolase
Gbem_2127		thiolase
Gbem_2835		thiolase
Gbem_3574		thiolase
Gbem_1442		acyl-CoA thioesterase superfamily protein
Gbem_2019		thioesterase superfamily protein
Gbem_2542		predicted thioesterase PaaI
Gbem_2846		thioesterase superfamily protein
Gbem_3071		long-chain acyl-CoA thioesterase, BFIT_BACH family
Gbem_3599		thioesterase superfamily protein
Gbem_3648		thioesterase family protein
Gbem_3898		thioesterase superfamily protein

Gbem_3982		thioesterase, 4HBT family
Gbem_0334-Gbem_0337		biotin-dependent oxaloacetate-dependent propionyl-CoA carboxyltransferase
Gbem_0562-Gbem_0561		biotin-dependent acyl-CoA carboxyltransferase
Gbem_1561-Gbem_1562 +Gbem_3091 +Gbem_3349		biotin-dependent acetyl-CoA carboxylase
Gbem_0558	*mce*	methylmalonyl-CoA epimerase
Gbem_0559 +Gbem_2691	*bhbA*	methylmalonyl-CoA mutase
Gbem_2838		methylmalonyl-CoA mutase
Gbem_0684		methylmalonyl-CoA decarboxylase
Gbem_1453	*gcdA*	glutaconyl-CoA decarboxylase
