# Supplementary material for: The genome of Geobacter bemidjiensis, exemplar for the subsurface clade of Geobacter species that predominate in Fe(III)-reducing subsurface environments
Source: BMC Genomics. 2010 Sep 9;11:490. doi: 10.1186/1471-2164-11-490 (PMC2996986; doi:10.1186/1471-2164-11-490)
Supplement: Additional file 3 — Table S3. Selected sugar interconversion genes of G. bemidjiensis. [file 1471-2164-11-490-S3.PDF]

Table S3. Selected sugar interconversion genes of *G. bemidjiensis*.

Gene	Functional annotation	Homologs or isozymes in *G. sulfurreducens*, *G. metallireducens*	References to characterized homologs
	**Glycogen pathway**		
Gbem_1326<br>Gbem_2002	glucose 6-kinase	GSU1702<br>Gmet_1639	[1-3]
Gbem_1567<br>Gbem_3964	phosphoglucomutase	GSU2013<br>GSU3321<br>Gmet_0135<br>Gmet_0990	
Gbem_0906	glucose-1-phosphate adenylyltransferase	Gmet_2768	[4]
	**4-amino-4-deoxy-L-arabinopyranose pathway**		
Gbem_1498	glucose-1-phosphate uridylyltransferase (*galU*)	GSU0859<br>Gmet_1159	[5]
Gbem_2287	UDP-glucose 6-dehydrogenase (*ugd*)	GSU1816<br>Gmet_1430	[6]
Gbem_2983	UDP-glucuronate decarboxylase (*arnA*)	Gmet_0883	[7]
Gbem_2982	UDP-L-arabinopyranose aminotransferase (*arnB*)	Gmet_0886	[8]
Gbem_2980	UDP-L-arabinosamine formyltransferase (*arnG*)	Gmet_0884	[7]
Gbem_2981	UDP:undecaprenyl phosphate *N*-formyl-L-arabinosamine transferase (*arnC*)	Gmet_0885	[9]
Gbem_2979	undecaprenylphospho-*N*-formyl-L-arabinosamine deformylase, putative (*arnD*)	GSU2557<br>Gmet_0882	
Gbem_2984	undecaprenyl:lipid A L-arabinosamine transferase, putative (*arnT*)	Gmet_0887	
	**Galacturonate branch**		
Gbem_0861	UDP-glucuronate 4-epimerase (*uge*)	GSU2241<br>Gmet_2330	[10]
	**Galactofuranose pathway**		
Gbem_4019	galactose 1-kinase (*galK*)	none	[11]
Gbem_4017	UDP-glucose:galactose-1-phosphate uridylyltransferase (*galT*)	GSU3256<br>Gmet_3176	[12]
Gbem_4014	UDP-galactopyranose mutase (*glf*)	none	[13]
	***N*-acetylglucosamine pathway**		
Gbem_0090	glucosamine-6-phosphate synthase (*glmS*)	GSU0270<br>Gmet_0104<br>Gmet_1487	[14]
Gbem_2635	phosphoglucosamine mutase (*glmM*)	GSU1805<br>Gmet_1886	[15]
Gbem_0091	glucosamine-1-phosphate *N*-acetyltransferase	GSU0271	[14]

	and *N*-acetylglucosamine-1-phosphate uridylyltransferase (*glmU*)	Gmet_0103	
Gbem_3707	UDP-*N*-acetylglucosamine 1-carboxyvinyltransferase (*murA*)	GSU3102<br>Gmet_0382	[16]
Gbem_0493<br>Gbem_3180	UDP-*N*-acetylenolpyruvoylglucosamine reductase (*murB-1*, *murB-2*)	GSU3067<br>Gmet_0414	[17]
***N*-acetylgalactosaminuronate branch**			
Gbem_3215	UDP-glucose/UDP-*N*-acetylglucosamine 4-epimerase (*galE*)	GSU2240<br>Gmet_2329<br>Gmet_1486	[18]
Gbem_1754<br>Gbem_1805	UDP-*N*-acetylgalactosamine 6-dehydrogenase (*capL-1*, *capL-2*)	GSU1942<br>Gmet_1994	[19]
***N*-acetylmannosamine branch**			
Gbem_2378<br>Gbem_1623	UDP-*N*-acetylglucosamine 2-epimerase (*wecB-1*, *wecB-2*)	GSU2243<br>GSU2245<br>Gmet_1489<br>Gmet_1504	[20]
**L-rhamnose pathway**			
Gbem_2464	glucose-1-phosphate thymidylyltransferase (*rfbA*)	GSU2083<br>Gmet_0924	[21]
Gbem_3346	dTDP-glucose 4,6-dehydratase (*rfbB*)		[21]
Gbem_2463	dTDP-4-dehydro-6-deoxyglucose 3,5-epimerase, putative (*rfbC*)	GSU2082<br>Gmet_0925	
Gbem_3345	dTDP-4-dehydro-L-rhamnose reductase (*rfbD*)		[21]
***N*-acetyl-3-amino-3,6-dideoxygalactose branch**			
Gbem_1609	dTDP-3-amino-3,6-dideoxy-D-galactose *N*-acetyltransferase and dTDP-6-deoxy-D-hex-4-ulose isomerase (*fdtC, fdtA*)	Gmet_1324	[22]
Gbem_1610	dTDP-6-deoxy-D-xylo-hex-3-ulose 3-aminotransferase (*fdtB*)	Gmet_1325	[22]
**Tyvelose pathway**			
Gbem_0855	glucose-1-phosphate cytidylyltransferase (*ddhA*)	none	[23]
Gbem_0856	CDP-glucose 4,6-dehydratase (*ddhB*)	none	[23]
Gbem_0857	CDP-4-dehydro-6-deoxyglucose reductase, dehydratase subunit (*ddhC*)	none	[23]
Gbem_0858	CDP-paratose synthase (*prt*)	none	[23, 24]
Gbem_0859	CDP-paratose 2-epimerase (*tyv*)	none	[23, 24]
**D-rhamnose pathway**			
Gbem_3344	mannose-6-phosphate isomerase	GSU2364<br>Gmet_2471	[25]
Gbem_1134	phosphomannomutase (*manB*)	GSU2013<br>GSU3321<br>Gmet_0135<br>Gmet_0990	[26]
Gbem_3343	mannose-1-phosphate guanylyltransferase	GSU1202	[26]

		Gmet_1104	
Gbem_1136	GDP-mannose 4,6-dehydratase and GDP-6-deoxy-D-*lyxo*-4-hexulose reductase (*gmd*)	GSU0626<br>Gmet_1311<br>Gmet_1488	[27]
**L-fucose branch**			
Gbem_0865 and Gbem_1135	GDP-L-fucose synthase (*fcl-1* and *fcl-2*)	GSU0627<br>Gmet_1312	[28]
**Perosamine branch**			
Gbem_2577	GDP-perosamine synthase	none	[29]

1. Angell S, Schwarz E, Bibb MJ: **The glucose kinase gene of *Streptomyces coelicolor* A3(2): its nucleotide sequence, transcriptional analysis and role in glucose repression**. *Mol Microbiol* 1992, **6**(19):2833-2844.
2. Mahr K, van Wezel GP, Svensson C, Krengel U, Bibb MJ, Titgemeyer F: **Glucose kinase of *Streptomyces coelicolor* A3(2): large-scale purification and biochemical analysis**. *Antonie Van Leeuwenhoek* 2000, **78**(3-4):253-261.
3. Meyer D, Schneider-Fresenius C, Horlacher R, Peist R, Boos W: **Molecular characterization of glucokinase from *Escherichia coli* K-12**. *J Bacteriol* 1997, **179**(4):1298-1306.
4. Baecker PA, Furlong CE, Preiss J: **Biosynthesis of bacterial glycogen. Primary structure of *Escherichia coli* ADP-glucose synthetase as deduced from the nucleotide sequence of the *glgC* gene**. *J Biol Chem* 1983, **258**(8):5084-5088.
5. Varon D, Boylan SA, Okamoto K, Price CW: ***Bacillus subtilis gtaB* encodes UDP-glucose pyrophosphorylase and is controlled by stationary-phase transcription factor sigma B**. *J Bacteriol* 1993, **175**(13):3964-3971.
6. Pagni M, Lazarevic V, Soldo B, Karamata D: **Assay for UDP-glucose 6-dehydrogenase in phosphate-starved cells: gene *tuaD* of *Bacillus subtilis* 168 encodes the UDP-glucose 6-dehydrogenase involved in teichuronic acid synthesis**. *Microbiology* 1999, **145 (Pt 5)**:1049-1053.
7. Breazeale SD, Ribeiro AA, Raetz CR: **Oxidative decarboxylation of UDP-glucuronic acid in extracts of polymyxin-resistant *Escherichia coli*. Origin of lipid A species modified with 4-amino-4-deoxy-L-arabinose**. *J Biol Chem* 2002, **277**(4):2886-2896.
8. Breazeale SD, Ribeiro AA, Raetz CR: **Origin of lipid A species modified with 4-amino-4-deoxy-L-arabinose in polymyxin-resistant mutants of *Escherichia coli*. An aminotransferase (ArnB) that generates UDP-4-deoxy-L-arabinose**. *J Biol Chem* 2003, **278**(27):24731-24739.
9. Breazeale SD, Ribeiro AA, McClerren AL, Raetz CR: **A formyltransferase required for polymyxin resistance in *Escherichia coli* and the modification of lipid A with 4-amino-4-deoxy-L-arabinose. Identification and function of UDP-4-deoxy-4-formamido-L-arabinose**. *J Biol Chem* 2005, **280**(14):14154-14167.

10. Regué M, Hita B, Pique N, Izquierdo L, Merino S, Fresno S, Benedi VJ, Tomás JM: **A gene, *uge*, is essential for *Klebsiella pneumoniae* virulence**. *Infect Immun* 2004, **72**(1):54-61.
11. Debouck C, Riccio A, Schumperli D, McKenney K, Jeffers J, Hughes C, Rosenberg M, Heusterspreute M, Brunel F, Davison J: **Structure of the galactokinase gene of *Escherichia coli*, the last (?) gene of the *gal* operon**. *Nucleic Acids Res* 1985, **13**(6):1841-1853.
12. Liebl W, Wagner B, Schellhase J: **Properties of an alpha-galactosidase, and structure of its gene *galA*, within an alpha-and beta-galactoside utilization gene cluster of the hyperthermophilic bacterium *Thermotoga maritima***. *Syst Appl Microbiol* 1998, **21**(1):1-11.
13. Nassau PM, Martin SL, Brown RE, Weston A, Monsey D, McNeil MR, Duncan K: **Galactofuranose biosynthesis in *Escherichia coli* K-12: identification and cloning of UDP-galactopyranose mutase**. *J Bacteriol* 1996, **178**(4):1047-1052.
14. Walker JE, Gay NJ, Saraste M, Eberle AN: **DNA sequence around the *Escherichia coli unc* operon. Completion of the sequence of a 17 kilobase segment containing *asnA*, *oriC*, *unc*, *glmS* and *phoS***. *Biochem J* 1984, **224**(3):799-815.
15. Dallas WS, Dev IK, Ray PH: **The dihydropteroate synthase gene, *folP*, is near the leucine tRNA gene, *leuU*, on the *Escherichia coli* chromosome**. *J Bacteriol* 1993, **175**(23):7743-7744.
16. Marquardt JL, Siegele DA, Kolter R, Walsh CT: **Cloning and sequencing of *Escherichia coli murZ* and purification of its product, a UDP-*N*-acetylglucosamine enolpyruvyl transferase**. *J Bacteriol* 1992, **174**(17):5748-5752.
17. Benson TE, Harris MS, Choi GH, Cialdella JI, Herberg JT, Martin JP, Jr., Baldwin ET: **A structural variation for MurB: X-ray crystal structure of *Staphylococcus aureus* UDP-*N*-acetylenolpyruvylglucosamine reductase (MurB)**. *Biochemistry* 2001, **40**(8):2340-2350.
18. Soldo B, Scotti C, Karamata D, Lazarevic V: **The *Bacillus subtilis* Gne (GneA, GalE) protein can catalyse UDP-glucose as well as UDP-*N*-acetylglucosamine 4-epimerisation**. *Gene* 2003, **319**:65-69.
19. Zhao X, Creuzenet C, Belanger M, Egbosimba E, Li J, Lam JS: **WbpO, a UDP-*N*-acetyl-D-galactosamine dehydrogenase from *Pseudomonas aeruginosa* serotype O6**. *J Biol Chem* 2000, **275**(43):33252-33259.
20. Namboori SC, Graham DE: **Acetamido sugar biosynthesis in the Euryarchaea**. *J Bacteriol* 2008, **190**(8):2987-2996.
21. Stevenson G, Neal B, Liu D, Hobbs M, Packer NH, Batley M, Redmond JW, Lindquist L, Reeves P: **Structure of the O antigen of *Escherichia coli* K-12 and the sequence of its *rfb* gene cluster**. *J Bacteriol* 1994, **176**(13):4144-4156.
22. Pfoestl A, Hofinger A, Kosma P, Messner P: **Biosynthesis of dTDP-3-acetamido-3,6-dideoxy-alpha-D-galactose in *Aneurinibacillus thermoaerophilus* L420-91T**. *J Biol Chem* 2003, **278**(29):26410-26417.
23. Jiang XM, Neal B, Santiago F, Lee SJ, Romana LK, Reeves PR: **Structure and sequence of the *rfb* (O antigen) gene cluster of *Salmonella* serovar *typhimurium* (strain LT2)**. *Mol Microbiol* 1991, **5**(3):695-713.

24. Verma N, Reeves P: **Identification and sequence of *rfbS* and *rfbE*, which determine antigenic specificity of group A and group D salmonellae**. *J Bacteriol* 1989, **171**(10):5694-5701.
25. Koeplin R, Arnold W, Hoette B, Simon R, Wang G, Puehler A: **Genetics of xanthan production in *Xanthomonas campestris*: the *xanA* and *xanB* genes are involved in UDP-glucose and GDP-mannose biosynthesis.** *J Bacteriol* 1992, **174**:191-199.
26. Aoyama K, Haase AM, Reeves PR: **Evidence for effect of random genetic drift on G+C content after lateral transfer of fucose pathway genes to *Escherichia coli* K-12**. *Mol Biol Evol* 1994, **11**(6):829-838.
27. King JD, Kocincova D, Westman EL, Lam JS: **Lipopolysaccharide biosynthesis in *Pseudomonas aeruginosa***. *Innate Immun* 2009.
28. Bonin CP, Reiter WD: **A bifunctional epimerase-reductase acts downstream of the MUR1 gene product and completes the *de novo* synthesis of GDP-L-fucose in *Arabidopsis***. *Plant J* 2000, **21**(5):445-454.
29. Cook PD, Holden HM: **GDP-perosamine synthase: structural analysis and production of a novel trideoxysugar**. *Biochemistry* 2008, **47**(9):2833-2840.
