# Supplementary material for: The genome of Geobacter bemidjiensis, exemplar for the subsurface clade of Geobacter species that predominate in Fe(III)-reducing subsurface environments
Source: BMC Genomics. 2010 Sep 9;11:490. doi: 10.1186/1471-2164-11-490 (PMC2996986; doi:10.1186/1471-2164-11-490)
Supplement: Additional file 4 — Table S4. Predicted c-type cytochromes of G. bemidjiensis. [file 1471-2164-11-490-S4.PDF]

Table S4. Predicted *c*-type cytochromes of *G. bemidjiensis*.

**Gene**	**Name**	**Heme-binding motifs**	**Homologs in *G. sulfurreducens* or *G. metallireducens***
Gbem_0046	*coxB*	1	GSU0222, Gmet_0252
Gbem_0116		1	no match
Gbem_0972		1	no match
Gbem_1211		1	no match
Gbem_1234		1	no match
Gbem_4098		1	no match
Gbem_2731		1	GSU1284, Gmet_2432
Gbem_2883		1	no match
Gbem_3352		1	no match
Gbem_3353		1	no match
Gbem_2356		1	no match
Gbem_1585		1	GSU2743, GSU2432 (OmcF), Gmet_0155
Gbem_2183		1	GSU2743, GSU2432 (OmcF), Gmet_0155
Gbem_3336		1	GSU3334
Gbem_1236		2	no match
Gbem_0818		2	no match
Gbem_0663		2	GSU0591, Gmet_2931
Gbem_3452		2	no match
Gbem_0020	*macA*	2	GSU0466, Gmet_3091
Gbem_0391		2	GSU2927, Gmet_0543
Gbem_1249		2	no match
Gbem_0120	*ccoP*	2	no match
Gbem_2955		2	no match
Gbem_0716		3	no match
Gbem_3455	*ppcG*	3	GSU0364, GSU0365, GSU0612, GSU1024, GSU1760, Gmet_0335, Gmet_1846, Gmet_2902, Gmet_3165, Gmet_3166
Gbem_3958		3	GSU0105
Gbem_4043	*ppcD*	3	GSU0364, GSU0365, GSU0612, GSU1024, GSU1760, Gmet_0335, Gmet_1846, Gmet_2902, Gmet_3165, Gmet_3166
Gbem_4049	*ppcB*	3	GSU0364, GSU0365, GSU0612, GSU1024, GSU1760, Gmet_0335, Gmet_1846, Gmet_2902, Gmet_3165, Gmet_3166

Gbem_2070	*nrfA-1*	4	GSU3154, Gmet_0294, Gmet_0296
Gbem_2929	*nrfA-2*	4	GSU3154, Gmet_0294, Gmet_0296
Gbem_3194		4	GSU0068, Gmet_0325, Gmet_3518
Gbem_3597		4	no match
Gbem_4036		4	no match
Gbem_4022		4	GSU2930, Gmet_0541
Gbem_1151		5	no match
Gbem_0666		5	no match
Gbem_1086		5	GSU1648, Gmet_1924
Gbem_2674		5	no match
Gbem_3059		5	no match
Gbem_4029		5	GSU2937, Gmet_0534
Gbem_1099		5	no match
Gbem_1100		5	no match
Gbem_0122		5	no match
Gbem_3373		5	GSU2725
Gbem_1116		6	GSU2504, GSU2503, GSU0701, GSU2501
Gbem_1117		6	GSU2504, GSU2503, GSU0701, GSU2501
Gbem_1131		6	GSU2504, GSU2503, GSU0701, GSU2501
Gbem_2679		6	GSU2504, GSU2503, GSU0701, GSU2501
Gbem_2680		6	GSU2504, GSU2503, GSU0701, GSU2501
Gbem_3374		6	no match
Gbem_1881		6	no match
Gbem_0676		7	GSU0594, Gmet_2928
Gbem_2884		7	no match
Gbem_2946		7	GSU0357
Gbem_3199		7	GSU3259, Gmet_3174
Gbem_3056	*omcZ*	8	GSU2076, GSU1334, Gmet_0930
Gbem_0758		8	no match
Gbem_3380		8	GSU2738, GSU2732, Gmet_0912, Gmet_0909
Gbem_3667		8	Gmet_0142
Gbem_0095		9	GSU0274, Gmet_0100
Gbem_1102		9	no match
Gbem_1155		9	no match
Gbem_2006		9	GSU3137, Gmet_0170
Gbem_4026		10	GSU2934, Gmet_0537
Gbem_3371		10	no match
Gbem_3118	*omcI*	10	GSU1228, Gmet_1744
Gbem_3354	*omcB*	10	GSU2737, Gmet_0913

Gbem_0584	*omcX*	12	GSU0670, Gmet_2839
Gbem_0679	*omcQ*	12	GSU0592, Gmet_2930
Gbem_2427		12	GSU2645, Gmet_0825
Gbem_1157		12	no match
Gbem_2323		12	GSU1996, Gmet_2048
Gbem_3379		12	no match
Gbem_4027		12	GSU2935, Gmet_0536
Gbem_1125		16	no match
Gbem_2869		25	no match
Gbem_1124		26	GSU2495
Gbem_1153		27	no match
Gbem_3470		27	no match
Gbem_3057		27	no match
Gbem_2888		27	no match
Gbem_1152		29	no match
Gbem_3052		31	no match
Gbem_1154		37	no match
