# Supplementary material for: The genome of Geobacter bemidjiensis, exemplar for the subsurface clade of Geobacter species that predominate in Fe(III)-reducing subsurface environments
Source: BMC Genomics. 2010 Sep 9;11:490. doi: 10.1186/1471-2164-11-490 (PMC2996986; doi:10.1186/1471-2164-11-490)
Supplement: Additional file 5 — Table S5. Signalling proteins of G. bemidjiensis. [file 1471-2164-11-490-S5.PDF]

Table S5. Signalling proteins of *G. bemidjiensis*.

**Gene**	**Domain architecture**	**Homologs in *G. sulfurreducens* or *G. metallireducens***
Gbem_0009	HAMP, PAS, HisKA, HATPase_c	GSU0007, Gmet_0009
Gbem_0010	REC, PAS, HisKA, HATPase_c	GSU0008, Gmet_0010
Gbem_0011	HisKA, HATPase_c	GSU009, Gmet_0011
Gbem_0012	REC, PAS_4, GAF, HisKA, HATPase_c	GSU0010
Gbem_0018	HisKA, HATPase_c	no match
Gbem_0052	PAS, GAF, HisKA, HATPase_c, REC	no match
Gbem_0065	REC, HisKA, HATPase_c, REC	no match
Gbem_0075	REC, PilZ	no match
Gbem_0080	REC, HDc	GSU1891
Gbem_0081	HAMP, PAS, PAS, GGDEF	no match
Gbem_0113	HAMP, GGDEF, EAL	no match
Gbem_0147	REC	GSU0104, Gmet_3414
Gbem_0149	REC	GSU3261, Gmet_3218
Gbem_0201	incomplete GGDEF	GSU1937, Gmet_1989
Gbem_0243	REC, transregC	GSU0451, Gmet_3383
Gbem_0270	GGDEF	GSU0952
Gbem_0272	Cache, HAMP, PAS, PAC, PAS, PAC, HisKA, HATPase_c, REC	no match
Gbem_0282	HAMP, HisKA, HATPase_c	GSU2916, Gmet_0554
Gbem_0283	REC, sigma54 interaction, HTH8	GSU2915, Gmet_0555
Gbem_0286	REC	GSU1090, Gmet_0762
Gbem_0314	HisKA, HATPase_c	no match
Gbem_0375 (*cheY-12*)	REC	no match
Gbem_0378	REC	no match
Gbem_0379	PAS, PAC, HisKA, HATPase_c, REC	no match
Gbem_0429	Cache, PAS, PAS, HisKA, HATPase_c, REC	no match
Gbem_0450	HisKA, HATPase_c	no match
Gbem_0451	REC, HTH LuxR	GSU0682
Gbem_0456	PAS, PAS, PAS, GGDEF, EAL	Gmet_3085
Gbem_0469	GAF, HisKA, HATPase_c, REC	no match
Gbem_0476	REC, PAS, GGDEF	GSU1656, Gmet_1917
Gbem_0498	GAF, HisKA, HATPase_c	no match
Gbem_0517	HAMP, PAS, HisKA, HATPase_c, REC	no match
Gbem_0522	REC	no match
Gbem_0535	PAS, HisKA, HATPase_c	no match

Gbem_0536	PAS, HisKA, HATPase_c, REC	no match
Gbem_0622 (*cheA-3*)	HPT, H-kinase_dim, HATPase_c, CheA_reg	GSU3199
Gbem_0623 (*cheY-7*)	REC	GSU3198, Gmet_3217
Gbem_0639	REC, HDc	GSU2094
Gbem_0649	GGDEF, EAL	Gmet_0782
Gbem_0655	HAMP, HisKA, HATPase_c	GSU0599, Gmet_2923
Gbem_0669	REC	GSU0596, Gmet_2926
Gbem_0671	REC, sigma54 interaction, HTH8	GSU0598, Gmet_2924
Gbem_0672	GAF, PAS, HisKA, HATPase_c, REC	GSU0842
Gbem_0673	REC, sigma54 interaction	GSU0841
Gbem_0675	REC	no match
Gbem_0681	REC, TPR	no match
Gbem_0710[1]	REC, PilZ	GSU0877, Gmet_3220
Gbem_0712 (*cheV*)	CheW, REC	GSU0879, Gmet_2739
Gbem_0731	REC, PP2C_SIG	no match
Gbem_0738	PATAN, REC, FRGAF	GSU2224
Gbem_0739 (*cheY-6*)	REC	GSU2223, Gmet_2312
Gbem_0740 (*cheA-2*)	HPT, H-kinase_dim, HATPase_c, CheA_reg	GSU2222, Gmet_2311
Gbem_0743 (*cheY-10*)	REC	GSU2219, Gmet_2308
Gbem_0745 (*cheY-9*)	REC	GSU2217
Gbem_0748 (*cheB-3*)	REC, CheB_methylest	GSU2214, Gmet_2304
Gbem_0750 (*cheY-5*)	REC	GSU2212, Gmet_2302
Gbem_0793	PAS, HisKA, HATPase_c, REC	no match
Gbem_0799	REC	no match
Gbem_0800	HisKA, HATPase_c, REC	no match
Gbem_0801	PAS, PAS, PAS, PAS, HisKA, HATPase_c, REC	no match
Gbem_0811	HisKA, HATPase_c, REC, Hpt	no match
Gbem_0873 (*ptsA*)	PTS_IIA_man	GSU1883, Gmet_1287
Gbem_0874 (*ptsH*)	PTS-Hpr_like	GSU1882, Gmet_1288
Gbem_0875 (*ptsI*)	PTS_I_fam	GSU1881, Gmet_1289
Gbem_0885	REC, HDc	GSU2094
Gbem_0900 (*ntrY*; for nitrogen)	HAMP, PAS, HisKA, HATPase_c	GSU0812, Gmet_0777
Gbem_0905	PAS, HisKA, HATPase_c, REC	no match
Gbem_0974	REC	no match
Gbem_0975	REC, PAS, PP2C_SIG	no match
Gbem_1017	SSF, PAS, HisKA, HATPase_c, REC	no match
Gbem_1018	REC, HATPase_c	no match
Gbem_1038 (*cheY-8*)	REC	Gmet_2827
Gbem_1039	PAS, GAF, HisKA, HATPase_c,	no match

	REC	
Gbem_1040	PAS, HisKA, HATPase_c	no match
Gbem_1046 (*cheA-5*)	HPT, H-kinase_dim, HATPase_c, CheA_reg	Gmet_1080
Gbem_1056 (*cpaE-1*; for Flp pilus)	REC, FlhG	no match
Gbem_1094	HisKA, HATPase_c	GSU1319, Gmet_2454
Gbem_1141	PAS, PAS, HisKA, HATPase_c	no match
Gbem_1171	HisKA, HATPase_c	no match
Gbem_1172[2] (*kdpE-1*; for osmosensitive potassium channel)	REC, transregC	GSU2484, Gmet_2437
Gbem_1179	HAMP, HATPase_c, REC	no match
Gbem_1202	GAF, GGDEF	GSU1870, Gmet_1298
Gbem_1227	Cache, HAMP, PAS, PAC, PAS, HisKA, HATPase_c, REC, HPT	no match
Gbem_1228	REC, GGDEF	no match
Gbem_1296	REC, HTH8	no match
Gbem_1332	REC, GGDEF	GSU3376, Gmet_0069
Gbem_1342	REC, transregC	GSU1879, Gmet_1291
Gbem_1343	HisKA, HATPase_c	GSU1878, Gmet_1292
Gbem_1383	Chase, PAS, HisKA, HATPase_c, REC	no match
Gbem_1425	HisKA, HATPase_c	Gmet_2146
Gbem_1426	REC, sigma54 interaction, HTH8	Gmet_2145
Gbem_1486	HAMP, HisKA, HATPase_c, REC	no match
Gbem_1505	HAMP, HisKA, HATPase_c, REC, Hpt	no match
Gbem_1506	REC, PAS, GGDEF, EAL	GSU1927
Gbem_1522	REC, sigma54 interaction	GSU0963, Gmet_0702
Gbem_1523	HAMP, HisKA, HATPase_c	GSU0962, Gmet_0701
Gbem_1530	REC	no match
Gbem_1533	HisKA, HATPase_c	GSU2042, Gmet_0960
Gbem_1534	REC, sigma54 interaction, HTH8	GSU2041, Gmet_0961
Gbem_1593 (*cheY-3*)	REC	GSU1289, Gmet_2428
Gbem_1594[3] (*cheA-1*)	HPT, H-kinase_dim, HATPase_c, CheA_reg	GSU1290, Gmet_2427
Gbem_1597 (*cheB-2*)	REC, CheB_methylest	GSU1145, Gmet_2418
Gbem_1642	REC, HisKA, HATPase_c	Gmet_2712
Gbem_1660	REC	GSU1117, Gmet_2679
Gbem_1662	REC, HisKA, HATPase_c	GSU1119, Gmet_2677
Gbem_1663	REC	GSU1120
Gbem_1701	PAS, PAC, HisKA, HATPase_c, REC	no match
Gbem_1708	REC, GAF, GGDEF, EAL	no match

Gbem_1806	GAF, HisKA, HATPase_c	GSU1941, Gmet_1993
Gbem_1807	REC, sigma54 interaction, HTH8	GSU1940, Gmet_1992
Gbem_1834 (*cpaE-2*; for Flp pilus)	REC, FlhG	no match
Gbem_1846[4]	REC, transregC	GSU1102, Gmet_2693
Gbem_1864	HAMP, PAS, HisKA, HATPase_c	no match
Gbem_1865[4]	REC, transregC	GSU1102, Gmet_2693
Gbem_1866	PAS, HisKA, HATPase_c	no match
Gbem_1919	PAS, HisKA, HATPase_c	no match
Gbem_1952	REC, HisKA, HATPase_c, REC	no match
Gbem_1953	PAS, PAC, HisKA, HATPase_c	no match
Gbem_1954	REC	no match
Gbem_1987	GAF, HisKA, HATPase_c	Gmet_1054
Gbem_1988	REC, sigma54 interaction, HTH8	Gmet_1055
Gbem_2023[5]	REC, sigma54 interaction, HTH8	GSU1250, Gmet_1082
Gbem_2024	REC, PAS, HisKA, HATPase_c	no match
Gbem_2025	REC, HisKA, HATPase_c	no match
Gbem_2026 (*cheB-8*)	REC, CheB_methylest	Gmet_2711
Gbem_2027 (*cheA-7*)	HPT, HATPase_c, CheA_reg, REC	no match
Gbem_2048	GAF, GGDEF	GSU0895, Gmet_2721
Gbem_2049	GAF, HisKA, HATPase_c	GSU3148
Gbem_2068	PAS, GGDEF	no match
Gbem_2069	PAS, PAS, GAF, PAS, PAC, HisKA, HATPase_c, REC	no match
Gbem_2115	HisKA, HATPase_c	no match
Gbem_2116	REC	no match
Gbem_2119	REC	no match
Gbem_2120	REC, HisKA, HATPase_c	no match
Gbem_2121	PAS, PAS, PAC, PAS, PAS, PAS, HATPase_c	no match
Gbem_2122	REC, PAS, PAS, PAS, GGDEF, EAL	no match
Gbem_2129	HisKA, HATPase_c	no match
Gbem_2131	PAS, PAC, PAS, PAS, PAS, HisKA, HATPase_c	no match
Gbem_2137	Cache, PAS, PAS, PAS, PAS, HisKA, HATPase_c, REC	no match
Gbem_2153	PAS, PAS, PAC, PAS, HisKA, HATPase_c	no match
Gbem_2155	PAS, HisKA, HATPase_c, REC	no match
Gbem_2160	REC, sigma54 interaction, HTH8	GSU0470
Gbem_2161	HisKA, HATPase_c	GSU0471
Gbem_2226	PAS, PAS, HisKA, HATPase_c, REC	no match
Gbem_2239[6]	REC, sigma54 interaction, HTH8	GSU0776, Gmet_1058

Gbem_2240[7]	HAMP, HisKA, HATPase_c	GSU0775, Gmet_1057
Gbem_2244	REC	no match
Gbem_2249	REC	no match
Gbem_2253	REC, HTH LuxR	GSU1293
Gbem_2254	HisKA_3, HATPase_c	no match
Gbem_2256	REC	no match
Gbem_2269[6]	REC, sigma54 interaction, HTH8	GSU0776, Gmet_1058
Gbem_2270[7]	HAMP, HisKA, HATPase_c	GSU0775, Gmet_1057
Gbem_2281[8]	GspIIEN, REC	GSU1220, Gmet_1737
Gbem_2335	HAMP, HisKA, HATPase_c	GSU1630, Gmet_1945
Gbem_2379 (*cheR-9*)	REC, CheR, TPR	no match
Gbem_2382 (*cheA-8*)	HPT, H-kinase_dim, HATPase_c, CheA_reg, REC	no match
Gbem_2383 (*cheB-9*)	REC, CheB_methylest	no match
Gbem_2384 (*cheY-13*)	REC	no match
Gbem_2386	HATPase_c	no match
Gbem_2392[5]	REC, sigma54 interaction, HTH8	GSU1250, Gmet_1082
Gbem_2394	REC, PAS, PAC, PAS, PAC, PP2C	GSU0700, Gmet_2818
Gbem_2406	REC, PAS, PAS. HisKA, HATPase_c, REC	GSU1655, Gmet_1918
Gbem_2407	REC, HDc	GSU1654, Gmet_1919
Gbem_2408	REC, sigma54 interaction, HTH8	GSU1653
Gbem_2416	HisKA, HATPase_c, REC	no match
Gbem_2419	PAC, HisKA, HATPase_c, REC	no match
Gbem_2420[8]	GspIIEN, REC	GSU1220, Gmet_1737
Gbem_2455	HisKA, HATPase_c	no match
Gbem_2494	REC, PAS, HisKA, HATPase_c, REC	no match
Gbem_2495	HAMP, HisKA, HATPase_c, REC, REC, REC	no match
Gbem_2516	GAF, PTS_I_fam	GSU1165, Gmet_2404
Gbem_2521	REC, sigma54 interaction, HTH8	GSU1129, Gmet_0812
Gbem_2534	REC, HATPase_c	no match
Gbem_2535	REC	no match
Gbem_2536	CHASE, PAS, PAS, HisKA, HATPase_c	no match
Gbem_2537	PAS, PAC, HisKA, HATPase_c	no match
Gbem_2591 (*pilR*; for geopilus biogenesis)	REC, sigma54 interaction, HTH8	GSU1495, Gmet_1397
Gbem_2592 (*pilS*; predicted for geopilus)	PAS, HisKA, HATPase_c	GSU1494, Gmet_1396
Gbem_2604[2] (*kdpE-2*; for osmosensitive potassium channel)	REC, transregC	GSU2484, Gmet_2437
Gbem_2605 (*kdpD*; for	HisKA, HATPase_c	GSU2483, Gmet_2436

osmosensitive potassium channel)		
Gbem_2657	HAMP, HisKA-HATPase_c	GSU2492
Gbem_2658	REC, sigma54 interaction, HTH8	GSU2524, Gmet_0555
Gbem_2665	HTH_XRE, REC	no match
Gbem_2669	PBPb, PAS, Hist_Kin_Sens, HisKA, HATPase_c, REC	no match
Gbem_2672[9]	REC, transregC	GSU2946, Gmet_0528
Gbem_2673	HAMP, HisKA, HATPase_c	GSU0452, Gmet_3382
Gbem_2715	REC, GGDEF	no match
Gbem_2716	HAMP, HisKA, HATPase_c	no match
Gbem_2803[1]	REC, PilZ	GSU0877, Gmet_3220
Gbem_2805	REC, sigma54 interaction, HTH8	GSU1003, Gmet_2563
Gbem_2806	PAS, HisKA, HATPase_c	GSU1004, Gmet_2562
Gbem_2810	REC	no match
Gbem_2821	GGDEF, EAL	no match
Gbem_2845	PAS, PAS, HisKA, HATPase_c	no match
Gbem_2865	PAS, PAS, HisKA, HATPase_c, REC	no match
Gbem_2922[10]	REC, sigma54 interaction, HTH8	GSU2753
Gbem_2923[11]	PBPb, HisKA, HATPase_c	GSU2755
Gbem_2931	GGDEF	no match
Gbem_2935	REC	no match
Gbem_2939	PBPb, PAS, Hist_Kin_Sens, HisKA, HATPase_c, REC	no match
Gbem_2940[3] (*cheA-9*)	HPT, H-kinase_dim, HATPase_c, CheA_reg	GSU1290, Gmet_2427
Gbem_2947	HisKA, HATPase_c, REC	no match
Gbem_2950	PAS, HisKA, HATPase_c, REC	no match
Gbem_2951	HAMP, PAS, HisKA, HATPase_c	GSU1101, Gmet_2694
Gbem_2952[4]	REC, transregC	GSU1102, Gmet_2693
Gbem_2962	CHASE, PAS, HisKA, HATPase_c, REC	no match
Gbem_3051	PAS, GAF, HisKA, HATPase_c, REC	no match
Gbem_3097	REC, GGDEF	GSU1658, Gmet_1914
Gbem_3127[8]	GspIIEN, REC	GSU1220, Gmet_1737
Gbem_3141	HisKA, HATPase_c	no match
Gbem_3142[5]	REC, sigma54 interaction, HTH8	GSU1250, Gmet_1082
Gbem_3143	PAS, HATPase_c	no match
Gbem_3181	HAMP, HisKA, HATPase_c, REC, Hpt	GSU1928
Gmet_3198	HTH_MerR_trunc, PTS_IIA_fru	
Gbem_3227[10]	REC, sigma54 interaction	GSU2753
Gbem_3228[11]	PBPb, HisKA, HATPase_c	GSU2755

Gbem_3231	PAS, HisKA, HATPase_c, REC	no match
Gbem_3274	REC, HisKA, HATPase_c	no match
Gbem_3275	PAS, HisKA, HATPase_c, REC	no match
Gbem_3279 (*nasR*)	REC, ANTAR	GSU2822, Gmet_0660
Gbem_3286 (*atoC*)	REC, sigma54 interaction, HTH8	GSU0811, Gmet_0776
Gbem_3295	HAMP, HisKA, HATPase_c	GSU3119
Gbem_3296	REC, transregC	GSU3118
Gbem_3327	PAS, HisKA, HATPase_c	no match
Gbem_3328	PAS, GGDEF	no match
Gbem_3366	HATPase_c	GSU2189, Gmet_2287
Gbem_3403	REC	GSU3253, Gmet_3179
Gbem_3405	GAF, HisKA, HATPase_c	GSU3252, Gmet_3180
Gbem_3413	REC, sigma54 interaction, HTH8	GSU1320, Gmet_2453
Gbem_3464	HAMP, HisKA, HATPase_c	GSU2947, Gmet_0527
Gbem_3465 (*cusR*)	REC, trans_reg_C	no match
Gbem_3531	GGDEF	no match
Gbem_3615[9] (*czcR*)	REC, transregC	GSU2946, Gmet_0528
Gbem_3616	HAMP, HisKA, HATPase_c	GSU2947, Gmet_0527
Gbem_3617	PAS, PAS, PAS, HisKA, HATPase_c, REC	no match
Gbem_3629	PAS, GAF, HisKA, HATPase_c	GSU0144, Gmet_0197
Gbem_3634	REC, HisKA, HATPase_c	GSU0149, Gmet_0202
Gbem_3818	HAMP, HisKA, HATPase_c	GSU0373, Gmet_3157
Gbem_3819	REC, sigma54 interaction, HTH8	GSU0372, Gmet_3158
Gbem_3845 (*cheY-1*)	REC	GSU0403, Gmet_3119
Gbem_3846	HisKA, HATPase_c	no match
Gbem_3878	PAS, PAS, HisKA, HATPase_c, REC	no match
Gbem_3940	REC, sigma54 interaction	GSU0298-GSU0300, Gmet_3263
Gbem_3942 (*cheA-4*)	HPT, H-kinase_dim, HATPase_c, CheA_reg	GSU0296, Gmet_3266
Gbem_3945 (*cheB-1*)	REC, CheB_methylest	GSU0293, Gmet_3269
Gbem_3991	GAF, HisKA, HATPase_c	no match
Gbem_4002	GAF, HisKA, HATPase_c	no match
Gbem_4038[10]	REC, sigma54 interaction, HTH8	GSU2753
Gbem_4039[11]	PBPb, HisKA, HATPase_c	GSU2755
Gbem_4041	PAS, PAC, HATPase_c	no match

[1]Duplicates.
[2]Duplicates.
[3]Duplicates.
[4]Triplicates.
[5]Triplicates.
[6]Duplicates.

[7]Duplicates.
[8]Triplicates.
[9]Duplicates.
[10]Triplicates.
[11]Triplicates.
