# Supplementary material for: The genome of Geobacter bemidjiensis, exemplar for the subsurface clade of Geobacter species that predominate in Fe(III)-reducing subsurface environments
Source: BMC Genomics. 2010 Sep 9;11:490. doi: 10.1186/1471-2164-11-490 (PMC2996986; doi:10.1186/1471-2164-11-490)
Supplement: Additional file 6 — Table S6. Multicopy nucleotide sequences in G. bemidjiensis. [file 1471-2164-11-490-S6.PDF]

Table S6. Multicopy nucleotide sequences in *G. bemidjiensis*.

**Locus tag**	**Nearest genes**	**Annotations of nearest genes**
**Palindromic sequences** - consensus RRAAARGGGGACAGGCTACTTTTSAWWTSAAAAGTAGCCTGTCCCCYTTTY (Figure S1)		
Gbem_R1001	3’ of Gbem_0018<br>5’ of Gbem_0019	sensor histidine kinase (HisKA, HATPase_c); URI domain endonuclease, putative
Gbem_R1002<br>Gbem_R1003	3’ of Gbem_0040<br>5’ of Gbem_0039	methyl-accepting chemotaxis sensory transducer with hemerythrin-like domain; ferrochelatase
Gbem_R1004	3’ of Gbem_0051<br>5’ of Gbem_0052	conserved hypothetical protein; sensor histidine kinase response receiver (PAS, GAF, HisKA, HATPase_c, REC)
Gbem_R1005<br>Gbem_R1006	3’ of Gbem_0085<br>3’ of Gbem_0086	aspartate/tyrosine/aromatic aminotransferase; alpha/beta hydrolase superfamily protein
Gbem_R1007	3’ of Gbem_0136<br>3’ of Gbem_0137	maltose-active trehalose synthase; ribonuclease BN family protein
Gbem_R1008	3’ of Gbem_0258<br>3’ of Gbem_0259	iron-sulfur cluster-binding oxidoreductase; succinyl-CoA synthetase, alpha subunit
Gbem_R1009<br>Gbem_R1010	3’ of Gbem_0289<br>3’ of Gbem_0290	FAD-dependent oxidase; glycoside hydrolase, family 15, DUF547-containing
Gbem_R1011	3’ of Gbem_0301<br>3’ of Gbem_0302	NADPH-dependent FMN reductase domain protein; [acyl-]glycerolphosphate acyltransferase and acyl-(acyl carrier protein) ligase, major facilitator superfamily domain-containing
Gbem_R1012	3’ of Gbem_0399<br>5’ of Gbem_0400	lipoate-protein ligase A; lipoate synthase
Gbem_R1013	3’ of Gbem_0428<br>5’ of Gbem_0427	membrane protein, MarC family; conserved hypothetical protein
Gbem_R1014	3’ of Gbem_0462<br>5’ of Gbem_0463	rhomboid-related membrane protein; uracil phosphoribosyltransferase
Gbem_R1015	3’ of Gbem_0610<br>3’ of Gbem_0611	transaldolase; membrane protein DedA
Gbem_R1016	5’ of Gbem_0713<br>5’ of Gbem_0714	molybdopterin-binding iron-sulfur cluster-binding oxidoreductase MopB-3; chromate transport protein ChrA, N-terminal domain, putative
Gbem_R1017<br>Gbem_R1018	3’ of Gbem_1135<br>5’ of Gbem_1134	GDP-L-fucose synthase; phosphomannomutase
Gbem_R1019<br>Gbem_R1020	3’ of Gbem_1360<br>3’ of Gbem_1361	radical SAM domain iron-sulfur cluster-binding oxidoreductase; conserved hypothetical protein
Gbem_R1021<br>Gbem_R1022	3’ of Gbem_1433<br>5’ of Gbem_1434	3-oxoacyl-(acyl carrier protein) reductase; thiolase
Gbem_R1023	3’ of Gbem_1696<br>5’ of Gbem_1697	RNA polymerase sigma factor; conserved hypothetical protein
Gbem_R1024	3’ of Gbem_1942<br>5’ of Gbem_1943	lipoprotein, putative; branched-chain amino acid ABC transporter, membrane protein

Gbem_R1025<br>Gbem_R1026	3' of Gbem_2072<br>5' of Gbem_2073	conserved hypothetical protein; homocitrate synthase
Gbem_R1027	3' of Gbem_2085<br>5' of Gbem_2086	electron transfer flavoprotein, beta subunit; electron transfer flavoprotein, alpha subunit
Gbem_R1028	3' of Gbem_2303<br>5' of Gbem_2302	dienelactone hydrolase family protein; DNA repair exonuclease SbcCD, D subunit, putative
Gbem_R1029<br>Gbem_R1030	3' of Gbem_2413<br>5' of Gbem_2412	anthranilate synthase protein I; sigma-54-dependent sensor transcriptional regulator (PAS, sigma54 interaction, HTH8)
Gbem_R1031	3' of Gbem_2703<br>5' of Gbem_2702	conserved hypothetical protein; SAM-dependent methyltransferase-like protein
Gbem_R1032	3' of Gbem_3052<br>5' of Gbem_3051	cytochrome *c*, 23-31 heme-binding sites; sensor histidine kinase response regulator (PAS, GAF, HisKA, HATPase_c, REC)
Gbem_R1033	3' of Gbem_3090<br>3' of Gbem_3091	protein of unknown function DUF445; acetyl-CoA carboxylase, carboxyl transferase, alpha subunit
Gbem_R1034<br>Gbem_R1035	3' of Gbem_3167<br>3' of Gbem_3168	glyoxylase-related zinc-dependent hydrolase; alpha/beta hydrolase superfamily protein
Gbem_R1036	3' of Gbem_3199<br>5' of Gbem_3198	cytochrome *c*, 7 heme-binding sites; helix-turn-helix transcriptional regulator, MerR family, PTSIIA domain-containing
Gbem_R1037	3' of Gbem_3202<br>5' of Gbem_3201	radical SAM domain iron-sulfur cluster-binding oxidoreductase; GTP-binding protein Era
Gbem_R1038	3' of Gbem_3229<br>5' of Gbem_3228	iron-sulfur cluster-binding oxidoreductase lipoprotein; periplasmic substrate-binding histidine kinase (PBPb, HisKA, HATPase_c)
Gbem_R1039<br>Gbem_R1040	3' of Gbem_3292<br>3' of Gbem_3293	desulfoferrodoxin; ATP-dependent RNA helicase RhlE
Gbem_R1041	3' of Gbem_3325<br>3' of Gbem_3326	rubrerythrin; conserved hypothetical protein
Gbem_R1042	3' of Gbem_3336<br>5' of Gbem_3337	lipoprotein cytochrome *c*, 1 heme-binding site; nucleoside triphosphate pyrophosphohydrolase MazG
Gbem_R1043	3' of Gbem_3356<br>3' of Gbem_3357<br>(overlapping)	conserved hypothetical protein; glycosyl transferase, putative
Gbem_R1044	3' of Gbem_3647<br>5' of Gbem_3648	glutamate-ammonia ligase adenylyltransferase; thioesterase family protein
Gbem_R1045	3' of Gbem_3654<br>5' of Gbem_3653	methyl-accepting chemotaxis sensory transducer; endonuclease/exonuclease/phosphatase family protein
**Palindromic sequences** - consensus RGGGGACTGGCTCCGCMAGGTGCCTGTCCCCCTT (Figure S1)		
Gbem_R1101	within Gbem_0067	hydrogenase subunit, iron-sulfur cluster-binding domain-containing
Gbem_R1102	3' of Gbem_0261	conserved hypothetical protein; succinyl-CoA

	5' of Gbem_0260	synthetase, beta subunit
Gbem_R1103	3' of Gbem_0276<br>5' of Gbem_0277	conserved hypothetical protein; 5-methyltetrahydrofolate--homocysteine methyltransferase
Gbem_R1104	3' of Gbem_0300<br>5' of Gbem_0301	conserved hypothetical protein, putative heme-binding site; NADPH-dependent FMN reductase domain protein
Gbem_R1105	3' of Gbem_0400<br>3' of Gbem_0401	lipoate synthase; mannitol dehydrogenase
Gbem_R1106	3' of Gbem_0631<br>5' of Gbem_0632	conserved hypothetical protein; conserved hypothetical protein
Gbem_R1107	3' of Gbem_0721<br>3' of Gbem_0722	protein of unknown function DUF833; lipoprotein, putative
Gbem_R1108	3' of Gbem_0765<br>5' of Gbem_0766	guanosine monophosphate synthase; HAD superfamily hydrolase
Gbem_R1109<br>Gbem_R1110	5' of Gbem_0790<br>5' of Gbem_0792	glycoside hydrolase, putative; ABC transporter, ATP-binding/membrane protein
Gbem_R1111	3' of Gbem_0908<br>5' of Gbem_0909	3-isopropylmalate/3-methylmalate dehydrogenase; aspartate-semialdehyde dehydrogenase
Gbem_R1112	3' of Gbem_1010<br>5' of Gbem_1011	putative transport protein, Tim44-like domain; conserved hypothetical protein
Gbem_R1113	3' of Gbem_1017<br>5' of Gbem_1018	sodium solute symporter and sensor histidine kinase response regulator (SSF, PAS, HisKA, HATPase_c, REC); response receiver histidine kinase (REC, HATPase_c)
Gbem_R1114	3' of Gbem_1194<br>3' of Gbem_1195	conserved hypothetical protein; major royal jelly-related protein
Gbem_R1115	3' of Gbem_1225<br>3' of Gbem_1226	membrane protein, LysE superfamily; iron-sulfur cluster-binding oxidoreductase
Gbem_R1116	3' of Gbem_1264<br>5' of Gbem_1265	aspartate kinase; citramalate synthase
Gbem_R1117	3' of Gbem_1291<br>5' of Gbem_1292	conserved hypothetical protein; succinic semialdehyde dehydrogenase
Gbem_R1118	3' of Gbem_1420<br>5' of Gbem_1421	indolepyruvate ferredoxin oxidoreductase, beta subunit; ACT domain protein
Gbem_R1119	3' of Gbem_1426<br>3' of Gbem_1427	sigma-54-dependent transcriptional response regulator (REC, sigma54 interaction, HTH8); conserved hypothetical protein
Gbem_R1120	3' of Gbem_1437<br>5' of Gbem_1438	formate dehydrogenase accessory protein FdhD, putative; sigma-54-dependent sensor transcriptional regulator (PAS, sigma54 interaction, HTH8)
Gbem_R1121	3' of Gbem_1448<br>5' of Gbem_1449	putative benzoyl-CoA reductase electron transfer protein, selenocysteine-containing; putative benzoyl-CoA reductase electron transfer protein, NADH dehydrogenase 24 kDa subunit-like
Gbem_R1122	3' of Gbem_1456	SAM-dependent methyltransferase, type 11; enoyl-

	5’ of Gbem_1457	CoA hydratase/isomerase
Gbem_R1123	3’ of Gbem_1465<br>5’ of Gbem_1466	succinyl-CoA synthetase, alpha subunit; electron transfer flavoprotein, beta subunit
Gbem_R1124	3’ of Gbem_1569<br>5’ of Gbem_1570	nitrogen fixation iron-sulfur cluster assembly protein NifS; ABC transporter, ATP-binding protein
Gbem_R1125	3’ of Gbem_1641<br>5’ of Gbem_1642	conserved hypothetical protein; response receiver histidine kinase (REC, HisKA, HATPase_c)
Gbem_R1126	3’ of Gbem_1650<br>5’ of Gbem_1651	AMP-forming acyl-CoA synthetase; Xaa-Pro dipeptidase
Gbem_R1127	3’ of Gbem_1710<br>5’ of Gbem_1711	phosphate ABC transporter, periplasmic phosphate-binding protein; YjgF/Yer057c/UK114 family protein
Gbem_R1128	within Gbem_1712	conserved hypothetical protein
Gbem_R1129	5’ of Gbem_1736<br>5’ of Gbem_1737	carbon monoxide dehydrogenase, catalytic subunit; sulfate ABC transporter, membrane protein CysU
Gbem_R1130	3’ of Gbem_1958<br>5’ of Gbem_1959	tryptophan synthase, homodimeric beta subunit; hypothetical protein
Gbem_R1131	5’ of Gbem_4103<br>5’ of Gbem_2037	4-oxalocrotonate tautomerase superfamily protein; putative hydrolase
Gbem_R1132	3’ of Gbem_2093<br>5’ of Gbem_2092	putative porin; conserved hypothetical protein
Gbem_R1133	3’ of Gbem_2102<br>5’ of Gbem_2101	3-oxoacyl-(acyl carrier protein) synthase III; haloalkane dehalogenase, putative
Gbem_R1134<br>Gbem_R1135<br>Gbem_R1136<br>Gbem_R1137	within Gbem_2105	polyketide synthase lipoprotein
Gbem_R1138	3’ of Gbem_2135<br>3’ of Gbem_2136	inner membrane protein of unknown function UPF0005; conserved hypothetical protein
Gbem_R1139<br>Gbem_R1140<br>Gbem_R1141	3’ of Gbem_2173<br>5’ of Gbem_2171	protein of unknown function DUF156; aconitate hydratase 1
Gbem_R1142	within Gbem_2225	cobalt ABC transporter, membrane protein CbiM
Gbem_R1143	5’ of Gbem_2225<br>5’ of Gbem_2226	cobalt ABC transporter, membrane protein CbiM; sensor histidine kinase response regulator (PAS, PAS, HisKA, HATPase_c, REC)
Gbem_R1144	3’ of Gbem_2233<br>3’ of Gbem_2234	exopolyphosphatase; chemotaxis protein CheW
Gbem_R1145	3’ of Gbem_2308<br>5’ of Gbem_2307	3-hydroxyisobutyrate dehydrogenase family protein; cell division ATP-dependent zinc protease lipoprotein FtsH
Gbem_R1146	3’ of Gbem_2330<br>5’ of Gbem_2329	phosphoribosylformylglycinamidine synthase, PurQ domain; glutamine phosphoribosylpyrophosphate amidotransferase

Gbem_R1147	3' of Gbem_2369<br>5' of Gbem_2368	efflux pump, RND family, membrane fusion lipoprotein; efflux pump, RND family, inner membrane protein
Gbem_R1148<br>Gbem_R1149	3' of Gbem_2707<br>5' of Gbem_2706	pyridoxal phosphate-dependent enzyme, class III; conserved hypothetical protein
Gbem_R1150	3' of Gbem_2836<br>5' of Gbem_2835	aminopeptidase, putative; thiolase
Gbem_R1151	3' of Gbem_2840<br>5' of Gbem_2839	sodium/solute symporter family protein; conserved hypothetical protein
Gbem_R1152	within Gbem_2885	membrane protein, putative
Gbem_R1153<br>Gbem_R1154<br>Gbem_R1155	3' of Gbem_2925<br>5' of Gbem_2926	ferritin-like domain protein; conserved thioredoxin domain protein
Gbem_R1156	3' of Gbem_2945<br>5' of Gbem_2944	methyl-accepting chemotaxis sensory transducer; methyl-accepting chemotaxis sensory transducer
Gbem_R1157	5' of Gbem_2952<br>5' of Gbem_2954	winged-helix transcriptional response regulator (REC, transregC); conserved membrane protein
Gbem_R1158<br>Gbem_R1159	3' of Gbem_2955<br>5' of Gbem_2956	lipoprotein cytochrome *c*, 2 heme-binding sites; peroxiredoxin, 1-Cys subfamily, selenocysteine-containing
Gbem_R1160<br>Gbem_R1161	3' of Gbem_3229<br>5' of Gbem_3228	iron-sulfur cluster-binding oxidoreductase lipoprotein; periplasmic substrate-binding histidine kinase (PBPb, HisKA, HATPase_c)
Gbem_R1162	3' of Gbem_3267<br>3' of Gbem_3268	conserved hypothetical protein; tRNA (5-carboxymethylaminomethyl-2-thio-U34)-thioltransferase
Gbem_R1163	3' of Gbem_3346<br>5' of Gbem_3345	dTDP-glucose 4,6-dehydratase; dTDP-4-dehydrorhamnose reductase
Gbem_R1164	3' of Gbem_3545<br>5' of Gbem_3544	cobalt-precorrin-8X methylmutase; cobalt-precorrin-5 *C*1-methyltransferase
Gbem_R1165	3' of Gbem_3610<br>5' of Gbem_3609	conserved hypothetical protein; glycerate 2-kinase
Gbem_R1166	3' of Gbem_3611<br>5' of Gbem_3612	methyl-accepting chemotaxis sensory transducer; ABC transporter, periplasmic substrate-binding protein
Gbem_R1167	3' of Gbem_3674<br>5' of Gbem_3673	magnesium chelatase, ChlI subunit, C-terminal fragment; conserved hypothetical protein
Gbem_R1168	3' of Gbem_3796<br>5' of Gbem_3797	L-seryl-tRNA(Sec) selenium transferase; 2-C-methyl-D-erythritol-4-phosphate cytidylyltransferase
Gbem_R1169	3' of Gbem_3880<br>5' of Gbem_3879	HAMP domain-containing methyl-accepting chemotaxis sensory transducer; SAM-dependent methyltransferase, type 12
Gbem_R1170	5' of Gbem_3991<br>5' of Gbem_3992	sensor histidine kinase (GAF, HisKA, HATPase_c); conserved hypothetical protein
Gbem_R1171	3' of Gbem_4007	multicopper oxidase; transcriptional regulator, TetR

	5’ of Gbem_4006	family
Gbem_R1172	3’ of Gbem_4054<br>5’ of Gbem_4053	dihydrodipicolinate synthase; dihydrodipicolinate reductase
**Palindromic sequences** - consensus KYYCCCCCTCCCCTTGACGGGAGGGGGYYRGGGGGTGGGKGAAGSYGCCA-NCWNYK(N_{0-6})MRNWGNTGGCRSCTTCMCCCACCCCCYRRCCCCCTCCCGTCA-AGGGGAGGGGGRRM (Figure S2)		
Gbem_R2001	3’ of Gbem_0066<br>5’ of Gbem_0067	cyclic nucleotide-binding helix-turn-helix transcriptional activator, Crp family; hydrogenase subunit, iron-sulfur cluster-binding domain-containing
Gbem_R2002	3’ of Gbem_0079<br>5’ of Gbem_0080	biosynthetic peptidoglycan transglycosylase; response receiver-modulated metal-dependent phosphohydrolase (REC, HDc)
Gbem_R2003	3’ of Gbem_0265<br>5’ of Gbem_0266	DNA polymerase IV (family X) domain and PHP phosphoesterase domain protein PRK08609; protein of unknown function DUF55
Gbem_R2004	3’ of Gbem_0303<br>5’ of Gbem_0302	conserved hypothetical protein; [acyl-]glycerolphosphate acyltransferase and acyl-(acyl carrier protein) ligase, major facilitator superfamily domain-containing
Gbem_R2005	3’ of Gbem_0340<br>5’ of Gbem_0339	DNA recombination protein, RmuC family; transposase of ISGbem_A
Gbem_R2006	3’ of Gbem_0345<br>5’ of Gbem_0344	exodeoxyribonuclease V, gamma subunit; exodeoxyribonuclease V, beta subunit
Gbem_R2007	3’ of Gbem_0428<br>5’ of Gbem_0427	membrane protein, MarC family; conserved hypothetical protein
Gbem_R2008	3’ of Gbem_0433<br>5’ of Gbem_0432	transposase of ISGbem_A; cyclic nucleotide-binding sigma-54-dependent transcriptional regulator (CAP_ED, sigma54 interaction)
Gbem_R2009	3’ of Gbem_0438<br>5’ of Gbem_0437	putative protein kinase (ABC1); lipoprotein release ABC transporter, ATP-binding protein
Gbem_R2010	3’ of Gbem_0488<br>5’ of Gbem_0489	phospho-*N*-acetylmuramoyl-pentapeptide transferase; UDP-*N*-acetylmuramoylalanine--D-glutamate ligase
Gbem_R2011	3’ of Gbem_0493<br>5’ of Gbem_0494	UDP-*N*-acetylenolpyruvoylglucosamine reductase; D-alanine--D-alanine ligase
Gbem_R2012	3’ of Gbem_0637<br>5’ of Gbem_0638	conserved hypothetical protein; conserved hypothetical protein
Gbem_R2013	3’ of Gbem_0647<br>5’ of Gbem_0648	extracellular solute-binding protein, family 1; extracellular solute-binding protein, family 1
Gbem_R2014	3’ of Gbem_0649<br>3’ of Gbem_0650	diguanylate cyclase/phosphoesterase (GGDEF, EAL); fumarylacetoacetate hydrolase family protein
Gbem_R2015	3’ of Gbem_0754<br>5’ of Gbem_0755	nucleotidyltransferase; DNA polymerase III, delta subunit
Gbem_R2016	3’ of Gbem_0763	glutamyl aminopeptidase M42; inosine-5'-

	5' of Gbem_0764	monophosphate dehydrogenase
Gbem_R2017	3' of Gbem_1167<br>5' of Gbem_1168	methyl-accepting chemotaxis sensory transducer; iron-sulfur-oxygen hybrid cluster protein (prismane)
Gbem_R2018	3' of Gbem_1230<br>5' of Gbem_1229	cytochrome oxidase, *cbb*$_3$-type, maturation lipoprotein, CcoS family; membrane protein, DsbD superfamily
Gbem_R2019	3' of Gbem_1347<br>3' of Gbem_1348	transposase of IS*Gbem_A*; pyranopterin monophosphate cyclase
Gbem_R2020	3' of Gbem_1350<br>5' of Gbem_1349	molybdopterin biosynthesis sulfur carrier protein sulfurylase; molybdopterin-molybdenum ligase
Gbem_R2021	3' of Gbem_1415<br>5' of Gbem_1416	thiolase; enoyl-CoA hydratase/isomerase
Gbem_R2022	3' of Gbem_1451<br>5' of Gbem_1452	iron-sulfur cluster-binding protein, putative; glutaryl-CoA dehydrogenase, non-decarboxylating
Gbem_R2023	3' of Gbem_1467<br>5' of Gbem_1468	electron transfer flavoprotein, alpha subunit; iron-sulfur cluster-binding oxidoreductase
Gbem_R2024	3' of Gbem_1710<br>5' of Gbem_1711	phosphate ABC transporter, periplasmic phosphate-binding protein; YjgF/Yer057c/UK114 family protein
Gbem_R2025	3' of Gbem_1724<br>5' of Gbem_1725	conserved hypothetical protein; *S*-adenosylmethionine synthetase
Gbem_R2026	3' of Gbem_1880<br>3' of Gbem_1881	aspartate/aromatic aminotransferase; lipoprotein cytochrome *c*, 6 heme-binding sites
Gbem_R2027	3' of Gbem_1882<br>5' of Gbem_1881	signal peptidase I; lipoprotein cytochrome *c*, 6 heme-binding sites
Gbem_R2028	3' of Gbem_1935<br>5' of Gbem_1936	membrane-bound zinc-dependent protease HtpX; membrane protein, TerC family
Gbem_R2029	3' of Gbem_1936<br>5' of Gbem_1937	membrane protein, TerC family; conserved hypothetical protein
Gbem_R2030	3' of Gbem_1956<br>5' of Gbem_1957	anthranilate phosphoribosyltransferase; indole-3-glycerol phosphate synthase
Gbem_R2031	3' of Gbem_1978<br>5' of Gbem_1977	dissimilatory sulfite reductase (desulfoviridin) subunit, putative; two-layered alpha/beta sandwich domain protein
Gbem_R2032	3' of Gbem_2029<br>5' of Gbem_2028	chemotaxis protein CheW; methyl-accepting chemotaxis sensory transducer
Gbem_R2033	3' of Gbem_2051<br>5' of Gbem_2050	lysine--8-amino-7-oxononanoate aminotransferase; magnesium-dependent deoxyribonuclease, TatD family, and radical SAM domain iron-sulfur oxidoreductase
Gbem_R2034	5' of Gbem_2082<br>5' of Gbem_2083	ADP-ribosyl-(dinitrogenase reductase) activating glycohydrolase; radical SAM domain iron-sulfur cluster-binding oxidoreductase
Gbem_R2035	3' of Gbem_2104<br>5' of Gbem_2103	polyketide synthase; 3-oxoacyl-(acyl carrier protein) synthase III

Gbem_R2036	3’ of Gbem_2163<br>5’ of Gbem_2162	conserved hypothetical protein; germane superfamily lipoprotein, putative
Gbem_R2037	3’ of Gbem_2165<br>5’ of Gbem_2164	metal ion efflux pump, RND family, membrane fusion protein; metal ion efflux pump, RND family, inner membrane protein
Gbem_R2038	3’ of Gbem_2178<br>3’ of Gbem_2179	pyridoxamine 5'-phosphate oxidase-related FMN-binding protein; conserved hypothetical protein
Gbem_R2039	3’ of Gbem_2411<br>5’ of Gbem_2410	proline dehydrogenase/Δ1-pyrroline-5-carboxylate dehydrogenase; fructose-1,6-bisphosphatase
Gbem_R2040	3’ of Gbem_2777<br>3’ of Gbem_2778	Glu/Leu/Phe/Val dehydrogenase superfamily protein; tRNA (*N*7-methyl-G46)-methyltransferase
Gbem_R2041	3’ of Gbem_2795<br>5’ of Gbem_2796	aspartate ammonia-lyase; thioredoxin/NifU-like domain protein
Gbem_R2042	3’ of Gbem_2911<br>3’ of Gbem_2912	iron-sulfur cluster-binding DUF162/cysteine-rich domain protein; conserved hypothetical protein
Gbem_R2043	3’ of Gbem_3192<br>5’ of Gbem_3191	Ech-hydrogenase-related complex NuoH-like integral membrane subunit; transposase of IS*Gbem_A*
Gbem_R2044	3’ of Gbem_3221<br>5’ of Gbem_3220	CopG-like DNA-binding protein; conserved hypothetical protein
Gbem_R2045	3’ of Gbem_3302<br>5’ of Gbem_3301	5,10-methylenetetrahydrofolate reductase; hypothetical protein
Gbem_R2046	3’ of Gbem_3544<br>5’ of Gbem_3543	cobalt-precorrin-5 *C*1-methyltransferase; cobalt-precorrin-6B *C*5,15-methyltransferase
Gbem_R2047	3’ of Gbem_3640<br>5’ of Gbem_3641	conserved hypothetical protein; argininosuccinate lyase
Gbem_R2048	3’ of Gbem_3645<br>5’ of Gbem_3646	aspartyl/glutamyl-tRNA(Asn/Gln) amidotransferase, B subunit; *S*-methyl-5-thio-alpha-D-ribose-1-phosphate isomerase
Gbem_R2049	3’ of Gbem_3769<br>5’ of Gbem_3768	radical SAM domain iron-sulfur cluster-binding oxidoreductase, TIGR01212 family; FRG domain protein
Gbem_R2050	3’ of Gbem_3773<br>5’ of Gbem_3772	flavodoxin 2 family protein; protein of unknown function DUF1121
Gbem_R2051	3’ of Gbem_3852<br>5’ of Gbem_3851	efflux pump, RND family, membrane fusion lipoprotein; efflux pump, RND family, inner membrane protein
Gbem_R2052	5’ of Gbem_3991<br>5’ of Gbem_3992	sensor histidine kinase (GAF, HisKA, HATPase_c); conserved hypothetical protein
**Palindromic sequences** - consensus [TGGCRSCTTCMCCCACCCCCYRR]CCCCCTCCCGTCAAGGGGAGGGGGRRM (Figure S2)		
Gbem_R2101	3’ of Gbem_0120<br>5’ of Gbem_0119	cytochrome cbb_3 oxidase, diheme subunit; conserved hypothetical protein
Gbem_R2102	3’ of Gbem_0270	diguanylate cyclase (GGDEF); nitrite/sulfite

	5’ of Gbem_0271	reductase domain protein
Gbem_R2103	3’ of Gbem_0309<br>5’ of Gbem_0310	outer membrane protein, putative; conserved hypothetical protein
Gbem_R2104	3’ of Gbem_0400<br>3’ of Gbem_0401	lipoate synthase; mannitol dehydrogenase
Gbem_R2105	3’ of Gbem_0408<br>5’ of Gbem_0409	hydroxymethylbilane synthase; uroporphyrinogen III *C*2,*C*7-methyltransferase and uroporphyrinogen III synthase
Gbem_R2106	3’ of Gbem_0446<br>5’ of Gbem_0447	TPR domain protein; conserved hypothetical protein
Gbem_R2107	3’ of Gbem_0573<br>3’ of Gbem_0574	RNA polymerase sigma-32 factor RpoH; acyl-phosphate:glycerol-3-phosphate acyltransferase
Gbem_R2108	3’ of Gbem_0627<br>5’ of Gbem_0626	aspartate/tyrosine/aromatic aminotransferase; chemotaxis protein methyltransferase CheR
Gbem_R2109	3’ of Gbem_0708<br>5’ of Gbem_0709	ATP-dependent protease, putative; sphingosine/diacylglycerol kinase-related protein
Gbem_R2110	3’ of Gbem_0785<br>5’ of Gbem_0786	metal-dependent hydrolase lipoprotein, beta-lactamase superfamily; SAM-dependent methyltransferase, type 11
Gbem_R2111	3’ of Gbem_0790<br>5’ of Gbem_0789	glycoside hydrolase, putative; lipoprotein, putative
Gbem_R2112	5’ of Gbem_1254<br>5’ of Gbem_1255	ParA family protein; exodeoxyribonuclease VII, large subunit
Gbem_R2113	3’ of Gbem_1411<br>5’ of Gbem_1412	sigma-54-dependent sensor transcriptional regulator (PAS4, GAF, sigma54 interaction, HTH-8); ATPase, BadF/BadG/BcrA/BcrD family, putative enzyme activator
Gbem_R2114	3’ of Gbem_1424<br>5’ of Gbem_1425	sodium/solute symporter family protein; sensor histidine kinase (HisKA, HATPase_c)
Gbem_R2115	3’ of Gbem_1426<br>3’ of Gbem_1427	sigma-54-dependent transcriptional response regulator (REC, sigma54 interaction, HTH8); conserved hypothetical protein
Gbem_R2116	3’ of Gbem_1429<br>5’ of Gbem_1428	benzoate CoA ligase; conserved hypothetical protein
Gbem_R2117	3’ of Gbem_1430<br>5’ of Gbem_1431	acetyl-CoA hydrolase/transferase; 6-hydroxycyclohex-1-ene-1-carbonyl-CoA dehydrogenase
Gbem_R2118	3’ of Gbem_1438<br>5’ of Gbem_1439	sigma-54-dependent sensor transcriptional regulator (PAS, sigma54 interaction, HTH8); acyl-CoA:carboxylate CoA transferase, putative
Gbem_R2119	3’ of Gbem_1439<br>3’ of Gbem_1440	acyl-CoA:carboxylate CoA transferase, putative; transcriptional regulator, Rrf2 family
Gbem_R2120	3’ of Gbem_1570<br>3’ of Gbem_1571	ABC transporter, ATP-binding protein; molybdopterin-guanine dinucleotide biosynthesis protein MobA
Gbem_R2121	3’ of Gbem_1707	aldehyde dehydrogenase family protein; response

	3’ of Gbem_1708	receiver sensor diguanylate cyclase/phosphoesterase (REC, GAF, GGDEF, EAL)
Gbem_R2122	3’ of Gbem_1818<br>5’ of Gbem_1819	metal-dependent hydrolase; PKD domain protein
Gbem_R2123	3’ of Gbem_1884<br>5’ of Gbem_1883	methyl-accepting chemotaxis sensory transducer; membrane GTPase LepA
Gbem_R2124	3’ of Gbem_1888<br>5’ of Gbem_1887	malate oxidoreductase, NADP-dependent (phosphate acetyltransferase-like domain fusion); carbonic anhydrase, beta-family, clade D
Gbem_R2125	3’ of Gbem_1898<br>5’ of Gbem_1899	carbamoyl-phosphate synthase, small subunit; PflX-related radical SAM domain iron-sulfur cluster-binding oxidoreductase
Gbem_R2126	3’ of Gbem_1988<br>5’ of Gbem_1989	sigma-54-dependent transcriptional response regulator (REC, sigma54 interaction, HTH8); glycosyl transferase, family 9 (heptosyltransferase)
Gbem_R2127	3’ of Gbem_2001<br>5’ of Gbem_2002	lipoprotein of unknown function DUF330; glucose 6-kinase
Gbem_R2128	3’ of Gbem_2022<br>3’ of Gbem_2023	malate:quinone oxidoreductase superfamily protein of unknown function PRK11728; sigma-54-dependent transcriptional response regulator (REC, sigma54 interaction, HTH8)
Gbem_R2129	3’ of Gbem_2028<br>5’ of Gbem_2027	methyl-accepting chemotaxis sensory transducer; chemotaxis sensor histidine kinase CheA and response receiver
Gbem_R2130	3’ of Gbem_2191<br>5’ of Gbem_2190	ACT domain protein; branched-chain amino acid ABC transporter, periplasmic amino acid-binding protein, putative
Gbem_R2131	3’ of Gbem_2303<br>5’ of Gbem_2302	dienelactone hydrolase family protein; DNA repair exonuclease SbcCD, D subunit, putative
Gbem_R2132	3’ of Gbem_2356<br>3’ of Gbem_2358	cytochrome *c*, selenocysteine-containing, 1 heme-binding motif; conserved hypothetical protein
Gbem_R2133	3’ of Gbem_2482<br>3’ of Gbem_2483	major facilitator superfamily MFS_1 protein; carbohydrate kinase, PfkB family
Gbem_R2134	3’ of Gbem_2486<br>5’ of Gbem_2485	conserved hypothetical protein; sodium-anion symporter, putative
Gbem_R2135	3’ of Gbem_2490<br>5’ of Gbem_2491	conserved hypothetical protein; conserved hypothetical protein
Gbem_R2136	3’ of Gbem_2630<br>5’ of Gbem_2631	multidrug resistance efflux pump, RND family, membrane fusion protein EmrA; multidrug resistance efflux pump, RND family, inner membrane protein EmrB
Gbem_R2137	5’ of Gbem_2656<br>5’ of Gbem_2657	UbiD family decarboxylase; sensor histidine kinase (HAMP, HisKA-HATPase_c)
Gbem_R2138	3’ of Gbem_2776<br>5’ of Gbem_2775	tRNA pseudouridine 13 synthase; 4-diphosphocytidyl-2-*C*-methyl-D-erythritol kinase
Gbem_R2139	3’ of Gbem_2802	conserved hypothetical protein; response regulator

	5’ of Gbem_2803	(REC, PilZ)
Gbem_R2140	3’ of Gbem_2832<br>5’ of Gbem_2831	short-chain acyl-CoA dehydrogenase; iron-sulfur cluster-binding oxidoreductase lipoprotein
Gbem_R2141	3’ of Gbem_2869<br>5’ of Gbem_2870	cytochrome *c*, 22-25 heme-binding sites; hypothetical protein
Gbem_R2142	5’ of Gbem_2885<br>5’ of Gbem_2886	membrane protein, putative; protein of unknown function UPF0066
Gbem_R2143	3’ of Gbem_2894<br>3’ of Gbem_2895	tRNA nucleotidyltransferase, putative; metal-dependent phosphohydrolase, HD superfamily
Gbem_R2144	5’ of Gbem_2919<br>5’ of Gbem_2920	transcriptional regulator PuuR (HTH_XRE, cupin); 5,10-methylenetetrahydrofolate dehydrogenase/methenyltetrahydrofolate cyclohydrolase
Gbem_R2145	3’ of Gbem_2922<br>5’ of Gbem_2921	sigma-54-dependent transcriptional response regulator (REC, sigma54 interaction, HTH8); anaerobic C4-dicarboxylate antiporter, Dcu family
Gbem_R2146	3’ of Gbem_2944<br>5’ of Gbem_2943	methyl-accepting chemotaxis sensory transducer; CheR-related putative SAM-binding domain protein
Gbem_R2147	3’ of Gbem_2947<br>5’ of Gbem_2946	sensor histidine kinase response regulator (HisKA, HATPase_c, REC); cytochrome *c*, 7 heme-binding sites
Gbem_R2148	5’ of Gbem_2952<br>5’ of Gbem_2954	winged-helix transcriptional response regulator (REC, transregC); conserved membrane protein
Gbem_R2149	3’ of Gbem_3196<br>3’ of Gbem_3197	oxidoreductase, membrane subunit; NAD-dependent dehydrogenase lipoprotein subunit
Gbem_R2150	3’ of Gbem_3276<br>5’ of Gbem_3275	ABC transporter, ATP-binding protein; sensor histidine kinase response regulator (PAS, HisKA, HATPase_c, REC)
Gbem_R2151	3’ of Gbem_3348<br>5’ of Gbem_3347	folylpolyglutamate synthetase; lipopolysaccharide biogenesis outer membrane protein LptD, putative
Gbem_R2152	3’ of Gbem_3503<br>5’ of Gbem_3502	phospholipase D superfamily protein; membrane carboxypeptidase (penicillin-binding protein, 1A family) MrcB (transglycosylase and transpeptidase)
Gbem_R2153	3’ of Gbem_3536<br>3’ of Gbem_3537	PilZ domain protein; L-threonine-*0*-3-phosphate decarboxylase
Gbem_R2154	3’ of Gbem_3542<br>5’ of Gbem_3541	cobalt-sirohydrochlorin *C*20-methyltransferase; cobalt-precorrin-4 *C*11-methyltransferase
Gbem_R2155	3’ of Gbem_3607<br>5’ of Gbem_3608	flavodoxin-related protein; conserved hypothetical protein
Gbem_R2156	3’ of Gbem_3608<br>3’ of Gbem_3609	conserved hypothetical protein; glycerate 2-kinase
Gbem_R2157	within Gbem_3616	sensor histidine kinase (HAMP, HisKA, HATPase_c)
Gbem_R2158	3’ of Gbem_3636<br>5’ of Gbem_3637	acetylornithine aminotransferase; ornithine carbamoyltransferase

Gbem_R2159	3’ of Gbem_3956<br>5’ of Gbem_3955	ParB-like nuclease domain protein, possible transcriptional regulator; ATP synthase F0, B' subunit
Gbem_R2160	5’ of Gbem_3958<br>5’ of Gbem_3959	cytochrome *c*, 3 heme-binding sites; sensor protein (HAMP, PAS), putative heme-binding site
Gbem_R2161	3’ of Gbem_3987<br>5’ of Gbem_3988	conserved hypothetical protein; peptidoglycan-binding ErfK/YbiS/YcfS/YnhG family lipoprotein
Gbem_R2162	3’ of Gbem_4058<br>5’ of Gbem_4057	tRNA (5-carboxymethylaminomethyl-2-thio-U34) modification GTPase; tRNA (5-carboxymethylaminomethyl-2-thio-U34)-formylglycinetransferase/reductase
**Cyclic diguanylate-responsive riboswitches** (conserved portion of GEMM sequences) (Figure S3)		
Gbem_R3001	3’ of Gbem_0308<br>5’ of Gbem_0309	membrane protein; outer membrane protein, putative
Gbem_R3002	3’ of Gbem_0538<br>5’ of Gbem_0537	conserved hypothetical protein; hypothetical protein
Gbem_R3003	5’ of Gbem_1080<br>5’ of Gbem_1081	conserved hypothetical protein; asparagine synthetase
Gbem_R3004	3’ of Gbem_1179<br>5’ of Gbem_1180	sensor histidine kinase response regulator (HAMP, HATPase_c, REC); conserved hypothetical protein
Gbem_R3005	3’ of Gbem_1797<br>5’ of Gbem_1798	lipoprotein, putative; fibronectin type III domain protein
Gbem_R3006	3’ of Gbem_1798<br>5’ of Gbem_1799	fibronectin type III domain protein; fibronectin type III domain protein
Gbem_R3007<br>Gbem_R3008	3’ of Gbem_1882<br>5’ of Gbem_1881	signal peptidase I; lipoprotein cytochrome *c*, 6 heme-binding sites
Gbem_R3009	5’ of Gbem_2146<br>5’ of Gbem_2147	protein of unknown function DUF500; oxidoreductase, short-chain dehydrogenase/reductase family
Gbem_R3010	3’ of Gbem_2256<br>5’ of Gbem_2255	response receiver (REC); hemerythrin family protein
Gbem_R3011	5’ of Gbem_2770<br>5’ of Gbem_2771	conserved hypothetical protein; conserved hypothetical protein
Gbem_R3012	3’ of Gbem_4121<br>5’ of Gbem_2966	hypothetical protein; lipoprotein, putative
Gbem_R3013<br>Gbem_R3014	5’ of Gbem_3987<br>5’ of Gbem_3988	conserved hypothetical protein; peptidoglycan-binding ErfK/YbiS/YcfS/YnhG family lipoprotein
**Palindromic sequences** - consensus CCCTCACCCCS[CCTTCGGS]SMCCCTCTCCCASMGGGNGAGGGRR (Figure S4)		
Gbem_R4001	3’ of Gbem_0015<br>5’ of Gbem_0016	radical SAM domain iron-sulfur cluster-binding oxidoreductase; uroporphyrinogen decarboxylase
Gbem_R4002	3’ of Gbem_0028<br>5’ of Gbem_0027	heterodisulfide reductase, iron-sulfur cluster-binding subunit, putative; heterodisulfide reductase, iron-sulfur cluster-binding subunit, putative

Gbem_R4003	3' of Gbem_0065<br>5' of Gbem_0064	response receiver histidine kinase response regulator (REC, HisKA, HATPase_c, REC); efflux pump, RND family, membrane fusion lipoprotein
Gbem_R4004	3' of Gbem_0077<br>5' of Gbem_0078	lipoprotein, putative; 4-hydroxythreonine-4-phosphate dehydrogenase
Gbem_R4005	3' of Gbem_0116<br>5' of Gbem_0115	cytochrome *c*, 1 heme-binding site; lipoprotein, putative
Gbem_R4006	3' of Gbem_0181<br>5' of Gbem_0180	zinc-dependent oxidoreductase; GspIIEN domain protein
Gbem_R4007	3' of Gbem_0305<br>5' of Gbem_0306	OmpA family outer membrane protein; arylamine *N*-acetyltransferase
Gbem_R4008<br>Gbem_R4009	3' of Gbem_0331<br>5' of Gbem_0330	conserved hypothetical protein; GAF sensor sigma-54-dependent transcriptional regulator (GAF, GAF, sigma54 interaction)
Gbem_R4010	3' of Gbem_0336<br>5' of Gbem_0337	conserved hypothetical protein; biotin/lipoyl attachment domain-containing protein
Gbem_R4011	3' of Gbem_0344<br>5' of Gbem_0343	exodeoxyribonuclease V, beta subunit; exodeoxyribonuclease V, alpha subunit
Gbem_R4012	3' of Gbem_0592<br>5' of Gbem_0591	type II secretion system pseudopilin GspG; type II secretion system protein H
Gbem_R4013	3' of Gbem_0673<br>5' of Gbem_0674	sigma-54-dependent transcriptional response regulator (REC, sigma54 interaction); conserved hypothetical protein
Gbem_R4014<br>Gbem_R4015	3' of Gbem_0755<br>3' of Gbem_0756	DNA polymerase III, delta subunit; ribosomal protein S20
Gbem_R4016	3' of Gbem_0969<br>5' of Gbem_0970	calcineurin-like phosphoesterase; tyrosyl-tRNA synthetase
Gbem_R4017	3' of Gbem_1041<br>5' of Gbem_1040	chemotaxis protein-glutamate methylesterase; sensor histidine kinase (PAS, HisKA, HATPase_c)
Gbem_R4018<br>Gbem_R4019	3' of Gbem_1248<br>5' of Gbem_4081	heme-copper oxidase subunit I superfamily protein; hypothetical protein
Gbem_R4020<br>Gbem_R4021<br>Gbem_R4022	3' of Gbem_1358<br>5' of Gbem_1359	electron transfer flavoprotein, alpha subunit; iron-sulfur cluster-binding oxidoreductase lipoprotein
Gbem_R4023	3' of Gbem_1422<br>5' of Gbem_1423	2-dehydropantoate 2-reductase; protein of unknown function DUF485
Gbem_R4024	3' of Gbem_1430<br>5' of Gbem_1431	acetyl-CoA hydrolase/transferase; 6-hydroxycyclohex-1-ene-1-carbonyl-CoA dehydrogenase
Gbem_R4025	3' of Gbem_1450<br>5' of Gbem_1451	putative benzoyl-CoA reductase electron transfer protein, NADH dehydrogenase 51 kDa subunit-like; iron-sulfur cluster-binding protein, putative
Gbem_R4026<br>Gbem_R4027	3' of Gbem_1452<br>5' of Gbem_1453	glutaryl-CoA dehydrogenase, non-decarboxylating; glutaconyl-CoA decarboxylase
Gbem_R4028<br>Gbem_R4029	3' of Gbem_1462<br>5' of Gbem_1463	enoyl-CoA hydratase/isomerase; iron-sulfur cluster-binding oxidoreductase

Gbem_R4030<br>Gbem_R4031	3’ of Gbem_1472<br>5’ of Gbem_1473	conserved hypothetical protein; conserved hypothetical protein
Gbem_R4032	3’ of Gbem_1718<br>5’ of Gbem_1717	phosphate ABC transporter, ATP-binding protein; phosphate transport system regulatory protein PhoU
Gbem_R4033<br>Gbem_R4034	3’ of Gbem_1732<br>3’ of Gbem_1733	acetyltransferase, GNAT family; ABC transporter, ATP-binding protein
Gbem_R4035<br>Gbem_R4036	3’ of Gbem_2041<br>5’ of Gbem_2042	transglutaminase-like cysteine protease; conserved hypothetical protein
Gbem_R4037	3’ of Gbem_2157<br>5’ of Gbem_2156	lipoprotein, putative; deoxycytidine triphosphate deaminase
Gbem_R4038<br>Gbem_R4039	3’ of Gbem_2169<br>5’ of Gbem_2168	conserved hypothetical protein; ABC transporter, membrane protein
Gbem_R4040	3’ of Gbem_2206<br>5’ of Gbem_2205	lipoprotein, putative; conserved hypothetical protein
Gbem_R4041	3’ of Gbem_2265<br>5’ of Gbem_2264	formate dehydrogenase, *b*-type cytochrome subunit, putative; formate dehydrogenase accessory and molybdopterin-guanine dinucleotide biosynthesis FdhD-MobA fusion protein
Gbem_R4042	3’ of Gbem_2376<br>5’ of Gbem_2375	peptidase, M23 family; conserved hypothetical protein
Gbem_R4043	3’ of Gbem_2498<br>5’ of Gbem_2497	protein of unknown function DUF1653; chemotaxis protein methyltransferase CheR
Gbem_R4044	3’ of Gbem_2555<br>5’ of Gbem_2556	conserved hypothetical protein; conserved hypothetical protein
Gbem_R4045<br>Gbem_R4046	3’ of Gbem_2619<br>5’ of Gbem_2618	iron-sulfur cluster-binding oxidoreductase, putative benzoyl-CoA reductase electron transfer protein; conserved hypothetical protein
Gbem_R4047	3’ of Gbem_2668<br>5’ of Gbem_2667	helicase, putative; lipoprotein of unknown function DUF2459
Gbem_R4048	3’ of Gbem_2739<br>3’ of Gbem_2740	cold shock DNA/RNA-binding protein; conserved hypothetical protein
Gbem_R4049	5’ of Gbem_2757<br>5’ of Gbem_2758	helix-turn-helix transcriptional regulator, IlvY family; ketol-acid reductoisomerase
Gbem_R4050	3’ of Gbem_2776<br>5’ of Gbem_2775	tRNA pseudouridine 13 synthase; 4-diphosphocytidyl-2-*C*-methyl-D-erythritol kinase
Gbem_R4051	3’ of Gbem_2823<br>5’ of Gbem_2822	conserved hypothetical protein; conserved hypothetical protein
Gbem_R4052	3’ of Gbem_2890<br>5’ of Gbem_2889	orotidine 5'-phosphate decarboxylase; 23S rRNA (2'-*O*-methyl-G2251)-methyltransferase
Gbem_R4053	3’ of Gbem_3015<br>5’ of Gbem_3014	peroxiredoxin-like 2 family protein, selenocysteine-containing; 6-phosphofructokinase, ATP-dependent
Gbem_R4054<br>Gbem_R4055	3’ of Gbem_3227<br>5’ of Gbem_3225	sigma-54-dependent transcriptional response regulator (REC, sigma54 interaction); sodium:C4-dicarboxylate symporter, DctA
Gbem_R4056	3’ of Gbem_3303	ABC transporter, ATP-binding protein; radical SAM

Gbem_R4057	3' of Gbem_3304	domain iron-sulfur cluster-binding oxidoreductase with cobalamin binding-like domain
Gbem_R4058	3' of Gbem_3308<br>5' of Gbem_3307	NDP-hexose 2,3-dehydratase; aminotransferase, AHBA_syn family
Gbem_R4059	5' of Gbem_3382<br>5' of Gbem_3383	major facilitator superfamily MFS_1 protein; Delta-1-pyrroline-5-carboxylate reductase
Gbem_R4060<br>Gbem_R4061<br>Gbem_R4062	3' of Gbem_3538<br>5' of Gbem_3537	adenosylcobinamide-phosphate synthase; L-threonine-*0*-3-phosphate decarboxylase
Gbem_R4063<br>Gbem_R4064<br>Gbem_R4065	3' of Gbem_3540<br>5' of Gbem_3539	cobalt-precorrin-5A hydrolase; cobalt-precorrin-3B *C*17-methyltransferase/adenosylcobyric acid synthase
Gbem_R4066	3' of Gbem_3632<br>5' of Gbem_3633	recombination regulator RecX; alanyl-tRNA synthetase
Gbem_R4067	3' of Gbem_3702<br>5' of Gbem_3701	imidazoleglycerol-phosphate synthase, glutamine amidotransferase subunit; phosphoribosylformimino-5-aminoimidazole carboxamide ribotide isomerase
Gbem_R4068	3' of Gbem_3703<br>5' of Gbem_3702	imidazoleglycerol-phosphate dehydratase; imidazoleglycerol-phosphate synthase, glutamine amidotransferase subunit
Gbem_R4069<br>Gbem_R4070<br>Gbem_R4071	3' of Gbem_3799<br>5' of Gbem_3800	glutaminyl-tRNA synthetase; cysteinyl-tRNA synthetase
Gbem_R4072<br>Gbem_R4073	3' of Gbem_3809<br>5' of Gbem_3808	4-amino-5-hydroxymethyl-2-methylpyrimidine synthetase; cobalt ABC transporter, membrane protein CbiM, N-terminal domain
Gbem_R4074	3' of Gbem_3994<br>3' of Gbem_3995	CheR-related putative SAM-binding domain protein; 3-oxoacyl-(acyl carrier protein) synthase III
Gbem_R4075	3' of Gbem_4007<br>5' of Gbem_4006	multicopper oxidase; transcriptional regulator, TetR family
Gbem_R4076	3' of Gbem_4025<br>5' of Gbem_4024	Rieske [2Fe-2S] domain protein; putative cytochrome *b*
**Conserved sequences** - consensus AAGTCCCCCTTTGCGAAGGGGGATTTAGGGGGATTTGC (Figure S5)		
Gbem_R5001	3' of Gbem_1453<br>5' of Gbem_1454	glutaconyl-CoA decarboxylase; oxaloacetate decarboxylase
Gbem_R5002<br>Gbem_R5003	3' of Gbem_1465<br>5' of Gbem_1466	succinyl-CoA synthetase, alpha subunit; electron transfer flavoprotein, beta subunit
Gbem_R5004	3' of Gbem_2071<br>5' of Gbem_2072	*O*-methyltransferase, family 2; conserved hypothetical protein
Gbem_R5005	3' of Gbem_3549<br>3' of Gbem_3550	3-deoxy-D-arabino-heptulosonate 7-phosphate (DAHP) synthase; type I restriction enzyme M protein
Gbem_R5006	3' of Gbem_4054<br>5' of Gbem_4053	dihydrodipicolinate synthase; dihydrodipicolinate reductase

**Conserved sequences** - consensus GCTTGGGAACCCAMTGCGKCGCAAAGCTCCAGCTTTGCATYC (Figure S5)		
Gbem_R5007	3' of Gbem_0156<br>3' of Gbem_4063	conserved hypothetical protein; conserved hypothetical protein
Gbem_R5009	3' of Gbem_1648<br>3' of Gbem_1649	lactate dehydrogenase-related protein; conserved hypothetical protein
Gbem_R5011	3' of Gbem_1824<br>5' of Gbem_1825	conserved hypothetical protein; DUF81 domain putative membrane protein
Gbem_R5013	3' of Gbem_2219<br>3' of Gbem_2220	cystathionine beta-lyase; conserved hypothetical protein
Gbem_R5015	5' of Gbem_2952<br>5' of Gbem_2954	winged-helix transcriptional response regulator (REC, transregC); conserved membrane protein
**Conserved sequences** - consensus GAAGCTGGAGCTTCGGNAGGCCTTGCGTTCCCAAGGTGGACCTTGGGAACGAGAT (Figure S5)		
Gbem_R5008	3' of Gbem_0156<br>3' of Gbem_4063	conserved hypothetical protein; conserved hypothetical protein
Gbem_R5010	3' of Gbem_1648<br>3' of Gbem_1649	lactate dehydrogenase-related protein; conserved hypothetical protein
Gbem_R5012	3' of Gbem_1824<br>5' of Gbem_1825	conserved hypothetical protein; DUF81 domain putative membrane protein
Gbem_R5014	3' of Gbem_2219<br>3' of Gbem_2220	cystathionine beta-lyase; conserved hypothetical protein
Gbem_R5016	5' of Gbem_2952<br>5' of Gbem_2954	winged-helix transcriptional response regulator (REC, transregC); conserved membrane protein
Gbem_R5017	3' of Gbem_0276<br>5' of Gbem_0277	conserved hypothetical protein; 5-methyltetrahydrofolate--homocysteine methyltransferase
Gbem_R5018	5' of Gbem_0303<br>5' of Gbem_0304	conserved hypothetical protein; sigma-54-dependent transcriptional regulator (sigma54 interaction)
**Conserved sequences** - consensus CGGGGACAYAATACCNATAATTCTGCAACACATGAGGNGCAGAAGAATYCAGTTCTCTYCTTCMCASATTRTTWTYSAGRMRAWGTWAAAGCCGCTACCGNCWARGTRGCGGCTTTTRTCNKRGTGAGCGAAGGGGGGTC (Figure S5)		
Gbem_R5019	5' of Gbem_0422<br>5' of Gbem_0423	conserved hypothetical protein; conserved domain protein
Gbem_R5020	3' of Gbem_2012<br>3' of Gbem_2013	NERD domain C4-type zinc finger protein; conserved hypothetical protein
Gbem_R5021	5' of Gbem_2881<br>5' of Gbem_4118	membrane protein, putative; ATP-binding protein
Gbem_R5022	3' of Gbem_2967<br>5' of Gbem_4121	hypothetical protein; hypothetical protein
Gbem_R5023	3' of Gbem_4122<br>5' of Gbem_2967	hypothetical protein; conserved hypothetical protein
Gbem_R5024	5' of Gbem_3975	single-strand binding protein; hypothetical protein

	3’ of Gbem_4143	
Gbem_R5025	3’ of Gbem_3976<br>3’ of Gbem_3977	conserved hypothetical protein; lipoprotein, putative
**Conserved sequences** - consensus<br>CGGGTGTGGCGGGGTCGGACCAAGGYARSATGYTGTAATGACACGAYAAWR<br>MCAGAMAAAGTCCATGTATTTTACCCTTAAAAGCATGTTTTCACCGGCAAAAA<br>GGGGCCAATAAAGCA (Figure S5)		
Gbem_R5026<br>Gbem_R5027<br>Gbem_R5028	3’ of Gbem_1913<br>5’ of Gbem_1914	hypothetical protein; putative transposase, DUF1568 domain-containing
Gbem_R5029<br>Gbem_R5030	3’ of Gbem_2968<br>5’ of Gbem_4123	conserved hypothetical protein; conserved hypothetical protein
**Conserved sequences** - consensus<br>CCATTCAGCACTTGACCTTTGACATNGATGATAGATCTCTATATATTGCGATTG<br>TTTAAGCTGGGTCAAATGAAATTTGCATATGAGTAGCGTNTGCATATTCCATTC<br>AGGGGGGGAGGGGGGATGGACAGTTTTCTGTCCATCCCCCCAATTGGCACAA<br>AATCCGGTCGTTACAGTGTGTCATATCAGTGGGTTACGCCAATNTGNTCGGNA<br>AAANATTTTAATGTTGNACGGCGACAAGGGGGGATGACGGCAATTTCCCTCTA<br>ATTACATAGTTA (Figure S5)		
Gbem_R5031	5’ of Gbem_0422<br>5’ of Gbem_0423	conserved hypothetical protein; conserved hypothetical protein
Gbem_R5032	3’ of Gbem_1242<br>5’ of Gbem_4078	large conductance mechanosensitive channel protein; lipoprotein, putative
Gbem_R5033	5’ of Gbem_4148<br>5’ of Gbem_1908	integrase domain protein; hypothetical protein
**Conserved sequences** - consensus<br>TTWCAACAGACTGTTTCTTTSGYGYSASYRGTGWTAATKTRWRA[C]$_{0-1}$TCAGCTAGCTAGYYAAGAGAWATCTCGRRMYAAGGAGGTAMACSRYYWTAG<br>AGGAAACYCCACCGATYAGGATTCTCAAAAMTTGAMRGYWGTAAYRGTCCG<br>GWSCKCAYAGGYWCCGGCATCCACCCTTGMAAAGGGTWCYCRATG[N]$_{0-2}$AGA[N]$_{0-11}$GAGGCCCGCCGRAWYCGGCGGGCCTTTCAATTT (Figure S5)		
Gbem_R5034<br>Gbem_R5035	3’ of Gbem_3499<br>5’ of Gbem_3498	lipoprotein, putative; phage integrase family protein
**Conserved sequences** - consensus<br>GGTTGACANANCGNCAGACANTAANTATATTTCACAAAGTTAAAGGGGAGTA<br>GNTATCGGCCGGAGAAATGGCCGACCCNGTGTTCGTCAAGACGGTNGNAANA<br>CCCGGACCCGGGGCKAKGATACCTCTNAKCAGCAAGACCTTTATCCARGCGN<br>NGACGTGCCGTGGGTAAAGGTCTTTTTA (Figure S5)		
Gbem_R5036	3’ of Gbem_1934<br>5’ of Gbem_1935	branched-chain amino acid ABC transporter, periplasmic amino acid-binding lipoprotein, putative; membrane-bound zinc-dependent protease HtpX
Gbem_R5037	5’ of Gbem_2936<br>5’ of Gbem_2937	tellurite resistance protein TehB-related putative methyltransferase; transcriptional regulator, CopG family
Gbem_R5038	3’ of Gbem_3279<br>5’ of Gbem_3280	response regulator (nitrate?) with putative antiterminator output domain (REC, ANTAR);

		membrane-bound zinc-dependent protease HtpX
**Conserved sequences** - consensus TCTCGCTATAGTGGCGAGAGGTTGAGGAGTATCGGCGCTCACTTCGGGTTTGR AGATTCTGCAGTGGTGAAGGCAAGCACGAGACTAGAAACAACAGCGAAACAA CCGTCCACGAAAAGGTCAAAGAGRTTCTGCAATCCYCTCGGTTGTCCAGGTCC WGACCWGACCCCTCAGCCCCYCT (Figure S5)		
Gbem_R5039<br>Gbem_R5040	3’ of Gbem_1481<br>3’ of Gbem_1482	conserved hypothetical protein; methyl-accepting chemotaxis sensory transducer
**Conserved sequences** - consensus GTTCCTCGTTASSATGAGGCGTTTGCWCAAACASAGGCTACTGCCCRRAAACG TCGAAAGACGCCAAYGGGTAGACCAGGKCTTGCCGGMTTAAGGCTTGACCYA AGGTAGCTGGTYCYYCSKGACCTACGTTGYGCACTGCYGAAAACCTACGAGW GGGGAA (Figure S5)		
Gbem_R5041	3’ of Gbem_0432<br>5’ of Gbem_0431	cyclic nucleotide-binding sigma-54-dependent transcriptional regulator (CAP_ED, sigma54 interaction); ABC transporter, membrane protein
Gbem_R5042	3’ of Gbem_3241<br>5’ of Gbem_3240	conserved hypothetical protein; putative cation transporter, CBS domain pair-containing
**Conserved sequences** - consensus AAAAAGCAAATGTWCCAAYAAGCCCCATGGACGCCGACCGCCCGAWRAAMY WGGKCGGGCGGGTCATGG (Figure S5)		
Gbem_R5045	3’ of Gbem_0420<br>5’ of Gbem_0419	conserved hypothetical protein; hypothetical protein
Gbem_R5046	3’ of Gbem_1911<br>5’ of Gbem_4097	conserved hypothetical protein; hypothetical protein
**Conserved sequences** - consensus AAAACNGTTCCACTTTTCCCAAAACCGGTTCAGCTTTTTCTAATCTGGTACAG TTTTG (Figure S5)		
Gbem_R5047<br>Gbem_R5048<br>Gbem_R5049	3’ of Gbem_2142<br>5’ of Gbem_2143	conserved hypothetical protein; lipoprotein, putative
**Conserved sequences** - consensus CCTCCASCTGTAAGYCTTCTGTAAGCCSCYCTCCATTAYCATCCCCGCAATAAG GGGC (Figure S5)		
Gbem_R5050	3’ of Gbem_3523<br>5’ of Gbem_3524	transcriptional activator; lipoprotein, putative
Gbem_R5051	3’ of Gbem_3524<br>5’ of Gbem_3525	lipoprotein, putative; hypothetical protein
**Conserved sequences** - consensus TTCAGTTCCMTTATCCTTTACTCTTGACGACGCCGACTGGTTTAGGCTAT (Figure S5)		
Gbem_R5052	3’ of Gbem_4121<br>5’ of Gbem_2966	hypothetical protein; lipoprotein, putative
Gbem_R5053	3’ of Gbem_4123<br>5’ of Gbem_4122	conserved hypothetical protein; conserved hypothetical protein

**Conserved sequences** - consensus AACAAAGGCACTTGGCTGATTAGCTAAGTGCCTTTTTG (Figure S5)		
Gbem_R5054	3' of Gbem_2866<br>3' of Gbem_2887	protein of unknown function UPF0066; hypothetical protein
Gbem_R5055	3' of Gbem_R0062<br>5' of Gbem_2888	tRNA-Met; cytochrome *c*, 26-27 heme-binding sites
**Conserved sequences** - consensus AAAATTARCTTGATCGSAAGATCTCTTTAATTTAAATT (Figure S5)		
Gbem_R5056	3' of Gbem_4087<br>5' of Gbem_1484	hypothetical protein; conserved hypothetical protein
Gbem_R5057	3' of Gbem_1489<br>5' of Gbem_1488	membrane protein of unknown function DUF1003; conserved hypothetical protein
**Conserved sequences** - consensus GTAGCTCCTTTWGCGCCAGGTCCYACGACCTGGCGCC (Figure S5)		
Gbem_R5058	3' of Gbem_1239<br>5' of Gbem_1240	iron-sulfur-oxygen hybrid cluster protein (prismane); OmpA family outer membrane protein (OprF, OmpA)
Gbem_R5059	3' of Gbem_1242<br>5' of Gbem_4078	large conductance mechanosensitive channel protein; lipoprotein, putative
**Conserved sequences** - consensus GCTCRCTTCAARGGGGCGGCATCTTGACGGCAC (Figure S5)		
Gbem_R5060<br>Gbem_R5061	3' of Gbem_2298<br>5' of Gbem_2297	tryptophanyl-tRNA synthetase; chromosome segregation and condensation protein ScpA
**Conserved sequences** - consensus GGAGGAGGTGGTGGTGAAATGGTGGAGGA (Figure S5)		
Gbem_R5062<br>Gbem_R5063	5' of Gbem_0501<br>5' of Gbem_0502	LexA repressor; hypothetical protein
**Conserved sequences** - consensus GACAAGGCCCGGTGTCTGTACGAATGGGT (Figure S5)		
Gbem_R5064	3' of Gbem_0319<br>5' of Gbem_0320	alkylmercury lyase; protein of unknown function DUF107
Gbem_R5065	3' of Gbem_0322<br>5' of Gbem_0323	alkylmercury lyase; protein of unknown function DUF107
**Conserved sequences** - consensus ACTGGCTCCGCAGGTGCCTGTCCCCCTT (Figure S5)		
Gbem_R5066<br>Gbem_R5067	5' of Gbem_3079<br>5' of Gbem_3080	glutamine synthetase, type III; zinc metalloendopeptidase, M23 family
**Conserved sequences** - consensus AGATAAAGRTTAAGTAAAGGCAGTAGG (Figure S5)		
Gbem_R5068<br>Gbem_R5069	3' of Gbem_2075<br>5' of Gbem_2076	nitrogenase molybdenum-iron protein, alpha chain; nitrogenase molybdenum-iron protein, beta chain
**Conserved sequences** - consensus TTTTTTYCAAATGTGTCATTTYCG (Figure S5)		
Gbem_R5070	3' of Gbem_0424	conserved hypothetical protein; conserved

Gbem_R5071	3' of Gbem_0425	hypothetical protein
**Conserved sequences** - consensus GGGGACGTTGAGCAACTGCTCTTT (Figure S5)		
Gbem_R5072<br>Gbem_R5073	3' of Gbem_0832<br>5' of Gbem_0833	biotin biosynthesis carboxylesterase BioH; biotin biosynthesis methyltransferase BioC
**Long repeat**- consensus [TTAGCCACGGAGAAAATCTGAGAGAGTCAAAAACCAAAACCGCTCACAAGGAGAACTTCTGAGGASTTCTGAGAAAGGCAAAACCGGAGAGAGTCAAAAACTTTYTCTGGGAAAGGCAAARCCAAAACC]$_{2\text{-}3}$ (Figure S5)		
Gbem_R5074	3' of Gbem_2714<br>3' of Gbem_2715	peptidase, U32 family; response receiver-modulated diguanylate cyclase (REC, GGDEF)
**Hexanucleotide repeats** - consensus [TCTYWA]$_n$TCT (Figure S6)		
Gbem_R6001	3' of Gbem_0002<br>5' of Gbem_0003	DNA polymerase III, beta subunit; DNA replication and repair protein RecF
Gbem_R6002	3' of Gbem_0008<br>5' of Gbem_0009	glycerol-3-phosphate dehydrogenase (NAD(P)+); sensor histidine kinase (HAMP, PAS, HisKA, HATPase_c)
Gbem_R6003<br>Gbem_R6004	3' of Gbem_0271<br>3' of Gbem_0272	nitrite/sulfite reductase domain protein; sensor histidine kinase response regulator (Cache, HAMP, PAS, PAC, PAS, PAC, HisKA, HATPase_c, REC)
Gbem_R6005<br>Gbem_R6006	3' of Gbem_0284<br>3' of Gbem_0285	conserved hypothetical protein; iron-sulfur cluster-binding oxidoreductase
Gbem_R6007	3' of Gbem_0491<br>5' of Gbem_0492	UDP-*N*-acetylglucosamine--*N*-acetylmuramyl-(pentapeptide) pyrophosphoryl-undecaprenol *N*-acetylglucosamine transferase; UDP-*N*-acetylmuramate--alanine ligase
Gbem_R6008	3' of Gbem_0597<br>5' of Gbem_0596	type II secretion system protein C, putative serine protease; ribosomal protein S23, putative
Gbem_R6009<br>Gbem_R6010	3' of Gbem_0620<br>5' of Gbem_0619	outer membrane lipoprotein carrier/sorting protein LolA; nucleotide-binding protein of unknown function DUF520
Gbem_R6011	3' of Gbem_0843<br>5' of Gbem_0844	acyl-(acyl carrier protein)--UDP-*N*-acetylglucosamine *O*-acyltransferase; lipid A disaccharide synthase
Gbem_R6012	3' of Gbem_1316<br>5' of Gbem_1317	conserved hypothetical protein; 6-carboxy-5,6,7,8-tetrahydropterin synthase
Gbem_R6014	3' of Gbem_1526<br>5' of Gbem_1527	preprotein translocase, SecA subunit; ornithine:glutamate *N*-acetyltransferase
Gbem_R6015	3' of Gbem_1561<br>5' of Gbem_1562	acetyl-CoA carboxylase, biotin carboxyl carrier protein; acetyl-CoA carboxylase, biotin carboxylase
Gbem_R6016<br>Gbem_R6017	3' of Gbem_2077<br>5' of Gbem_2078	nitrogenase molybdenum-iron cofactor biosynthesis protein NifEN; nitrogenase molybdenum-iron cofactor biosynthesis protein NifX
Gbem_R6018	3' of Gbem_2186<br>5' of Gbem_2185	branched-chain amino acid ABC transporter, ATP-binding protein; branched-chain amino acid ABC transporter, ATP-binding protein

Gbem_R6019<br>Gbem_R6020	3' of Gbem_2237<br>5' of Gbem_2236	cation transport ATPase, E1-E2 family; Kef-type potassium transporter, NAD-binding protein
Gbem_R6021	3' of Gbem_2334<br>5' of Gbem_2333	metal-dependent hydrolase, beta-lactamase superfamily; adenylosuccinate lyase
Gbem_R6022<br>Gbem_R6023	3' of Gbem_2337<br>5' of Gbem_2338	phosphoglycerate kinase; triosephosphate isomerase
Gbem_R6024	3' of Gbem_2977<br>5' of Gbem_2976	protein of unknown function DUF86; lipoprotein, putative
Gbem_R6025	3' of Gbem_3022<br>5' of Gbem_3021	3,4-dihydroxy-2-butanone-4-phosphate synthase/GTP cyclohydrolase II; 6,7-dimethyl-8-ribityllumazine synthase
Gbem_R6026	3' of Gbem_3132<br>5' of Gbem_3131	hydrogenase maturation protein HypF; hydrogenase assembly chaperone HypC/HupF
Gbem_R6028	3' of Gbem_3551<br>5' of Gbem_3552	conserved hypothetical protein; peptidase, U32 family
Gbem_R6029	3' of Gbem_3629<br>5' of Gbem_3630	sensor histidine kinase (PAS, GAF, HisKA, HATPase_c), putative heme-binding site; recombinase A
Gbem_R6030	3' of Gbem_3701<br>5' of Gbem_3700	phosphoribosylformimino-5-aminoimidazole carboxamide ribotide isomerase; imidazoleglycerol-phosphate synthase, cyclase subunit
Gbem_R6031<br>Gbem_R6032	3' of Gbem_3839<br>5' of Gbem_3838	flagellar hook-basal body complex protein FliE; flagellar M-ring mounting plate protein FliF
Gbem_R6033<br>Gbem_R6034	3' of Gbem_3994<br>3' of Gbem_3995	CheR-related putative SAM-binding domain protein; 3-oxoacyl-(acyl carrier protein) synthase III
**Hexanucleotide repeats** - consensus $[GYGKYT]_{14}$ (Figure S6)		
Gbem_R6013	within Gbem_1374	conserved hypothetical protein
**Hexanucleotide repeats** - consensus $[GGGTCA]_6$ (Figure S6)		
Gbem_R6027	3' of Gbem_3521<br>5' of Gbem_3522	endonuclease III-related protein; conserved hypothetical protein
**Heptanucleotide repeats** - consensus $[CAATTGA]_nCAA$ (Figure S7)		
Gbem_R7001	3' of Gbem_0011<br>5' of Gbem_0012	sensor histidine kinase (HisKA, HATPase_c); response receiver histidine kinase (REC, PAS_4, GAF, HisKA, HATPase_c)
Gbem_R7002	3' of Gbem_0023<br>5' of Gbem_0022	membrane protein of unknown function DUF6; NADPH ferredoxin oxidoreductase (FNOR) beta subunit
Gbem_R7003	3' of Gbem_0028<br>5' of Gbem_0027	heterodisulfide reductase, iron-sulfur cluster-binding subunit, putative; heterodisulfide reductase, iron-sulfur cluster-binding subunit, putative
Gbem_R7004<br>Gbem_R7005<br>Gbem_R7006<br>Gbem_R7007<br>Gbem_R7008	5' of Gbem_0082<br>5' of Gbem_0083	thiamin biosynthesis protein ThiI-related adenine nucleotide alpha hydrolase superfamily protein; PilB/PulE/GspE family ATPase

Gbem_R7009		
Gbem_R7010	3’ of Gbem_0150<br>3’ of Gbem_0151	excinuclease ABC, B subunit; nucleic acid-independent polyadenylating polymerase
Gbem_R7011	3’ of Gbem_0235<br>5’ of Gbem_0236	radical SAM domain iron-sulfur cluster-binding oxidoreductase; histidinol-phosphate aminotransferase
Gbem_R7012<br>Gbem_R7013	3’ of Gbem_0256<br>3’ of Gbem_0257	conserved hypothetical protein; methyl-accepting chemotaxis sensory transducer
Gbem_R7014	3’ of Gbem_0342<br>5’ of Gbem_0341	conserved hypothetical protein; hydrolase or acyltransferase, alpha/beta fold family
Gbem_R7015<br>Gbem_R7016	3’ of Gbem_0343<br>5’ of Gbem_0342	exodeoxyribonuclease V, alpha subunit; conserved hypothetical protein
Gbem_R7017<br>Gbem_R7018	3’ of Gbem_0564<br>5’ of Gbem_0563	D-lactate/glycolate dehydrogenase, FAD-binding protein, putative; D-lactate/glycolate dehydrogenase, iron-sulfur cluster-binding protein, putative
Gbem_R7019	3’ of Gbem_0881<br>3’ of Gbem_0882	conserved hypothetical protein; asparaginyl-tRNA synthetase
Gbem_R7020<br>Gbem_R7021	3’ of Gbem_0887<br>5’ of Gbem_0888	metal-dependent phosphohydrolase (HDc); conserved hypothetical protein
Gbem_R7022	3’ of Gbem_0909<br>5’ of Gbem_0910	aspartate-semialdehyde dehydrogenase; aspartate-semialdehyde dehydrogenase, USG-1 related
Gbem_R7023<br>Gbem_R7024	3’ of Gbem_1180<br>5’ of Gbem_1181	conserved hypothetical protein; 3-methyladenine DNA glycosylase I
Gbem_R7025	3’ of Gbem_1189<br>5’ of Gbem_1188	conserved hypothetical protein; ferritin-like domain protein
Gbem_R7026	3’ of Gbem_1454<br>5’ of Gbem_1455	oxaloacetate decarboxylase; malate oxidoreductase, NADP-dependent (phosphate acetyltransferase-like domain fusion)
Gbem_R7027	3’ of Gbem_1564<br>3’ of Gbem_1565	periplasmic solute-binding protein DUF178; NUDIX hydrolase
Gbem_R7028	3’ of Gbem_1588<br>5’ of Gbem_1589	ferrous iron transport protein B; conserved hypothetical protein
Gbem_R7029	3’ of Gbem_1925<br>3’ of Gbem_1926	protein of unknown function, 3-demethylubiquinone-9 3-methyltransferase family; toxin Txe
Gbem_R7030	3’ of Gbem_2052<br>5’ of Gbem_2051	dethiobiotin synthetase; lysine--8-amino-7-oxononanoate aminotransferase
Gbem_R7031<br>Gbem_R7032	3’ of Gbem_2185<br>5’ of Gbem_2184	branched-chain amino acid ABC transporter, ATP-binding protein; phenylacetate-coenzyme A ligase
Gbem_R7033<br>Gbem_R7034	3’ of Gbem_2412<br>5’ of Gbem_2411	sigma-54-dependent sensor transcriptional regulator (PAS, sigma54 interaction, HTH8); proline dehydrogenase/Δ1-pyrroline-5-carboxylate dehydrogenase
Gbem_R7035	3’ of Gbem_2459<br>5’ of Gbem_2458	rod shape-determining protein RodA; glycosyl transferase, putative
Gbem_R7036	3’ of Gbem_2631	multidrug resistance efflux pump, RND family, inner

	3' of Gbem_2632	membrane protein EmrB; conserved hypothetical protein
Gbem_R7037	3' of Gbem_2644<br>5' of Gbem_2643	peptide ABC transporter, periplasmic peptide-binding lipoprotein; peptide ABC transporter, membrane protein
Gbem_R7038	3' of Gbem_2737<br>5' of Gbem_2736	isopropylmalate/citramalate isomerase, large subunit; isopropylmalate/citramalate isomerase, small subunit
Gbem_R7039<br>Gbem_R7040	3' of Gbem_2746<br>5' of Gbem_2745	acetolactate synthase, large subunit, biosynthetic type; acetolactate synthase, small subunit
Gbem_R7041	3' of Gbem_3080<br>3' of Gbem_3081	zinc metalloendopeptidase, M23 family; ATP-dependent helicase HrpB
Gbem_R7042	3' of Gbem_3314<br>5' of Gbem_3315	crossover junction endodeoxyribonuclease RuvC; Holliday junction DNA helicase RuvA
Gbem_R7043<br>Gbem_R7044	3' of Gbem_3316<br>5' of Gbem_3317	Holliday junction DNA helicase RuvB; DnaA regulatory inactivator Hda
Gbem_R7045<br>Gbem_R7046	3' of Gbem_3317<br>3' of Gbem_3318	DnaA regulatory inactivator Hda; ABC transporter, ATP-binding protein
Gbem_R7047	3' of Gbem_3385<br>3' of Gbem_3386	protein disulfide bond isomerase, DsbC/DsbG-like; radical SAM domain iron-sulfur cluster-binding oxidoreductase
Gbem_R7048<br>Gbem_R7049<br>Gbem_R7050	3' of Gbem_3458<br>5' of Gbem_3457	phosphoribosylaminoimidazolecarboxamide formyltransferase/IMP cyclohydrolase; phosphoribosylamine--glycine ligase
Gbem_R7051	3' of Gbem_3554<br>5' of Gbem_3555	chaperonin Hsp33; putative cyclase/hydrolase
Gbem_R7052	3' of Gbem_3639<br>5' of Gbem_3640	NUDIX hydrolase; conserved hypothetical protein
Gbem_R7053<br>Gbem_R7054<br>Gbem_R7055	3' of Gbem_3795<br>5' of Gbem_3796	transcriptional regulator, GntR family; L-seryl-tRNA(Sec) selenium transferase
Gbem_R7056	3' of Gbem_3878<br>3' of Gbem_3879	sensor histidine kinase response regulator (PAS, PAS, HisKA, HATPase_c, REC); SAM-dependent methyltransferase, type 12
**Heptadecanucleotide repeats** - consensus $[CAATTGACGGCGTAACC]_3$ (Figure S7)		
Gbem_R7057	3' of Gbem_2746<br>5' of Gbem_2745	acetolactate synthase, large subunit, biosynthetic type; acetolactate synthase, small subunit
**Heptanucleotide repeats** - consensus $[AACGTTG]_nAAC$ (Figure S7)		
Gbem_R7058<br>Gbem_R7059	3' of Gbem_0030<br>5' of Gbem_0029	heterodisulfide reductase, subunit A; methyl viologen-reducing hydrogenase, iron-sulfur cluster-containing subunit, MvhD
Gbem_R7060	3' of Gbem_0158<br>5' of Gbem_0159	radical SAM domain iron-sulfur cluster-binding oxidoreductase; ribonuclease G
Gbem_R7061<br>Gbem_R7062	3' of Gbem_0276<br>5' of Gbem_0277	conserved hypothetical protein; 5-methyltetrahydrofolate--homocysteine methyltransferase

Gbem_R7063<br>Gbem_R7064<br>Gbem_R7065	3' of Gbem_0479<br>5' of Gbem_0480	protein of unknown function DUF164; conserved hypothetical protein
Gbem_R7066<br>Gbem_R7067<br>Gbem_R7068<br>Gbem_R7069	3' of Gbem_0739<br>5' of Gbem_0740	chemotaxis protein CheY; chemotaxis sensor histidine kinase CheA
Gbem_R7070	3' of Gbem_0840<br>5' of Gbem_0841	OmpH-like outer membrane protein, putative; UDP-3-*O*-(3-hydroxymyristoyl)-glucosamine *N*-acyltransferase
Gbem_R7071	3' of Gbem_0862<br>5' of Gbem_0863	conserved hypothetical protein; conserved hypothetical protein
Gbem_R7072	3' of Gbem_0863<br>5' of Gbem_0864	conserved hypothetical protein; D-glycero-D-mannoheptose-7-phosphate kinase and D-glycero-D-mannoheptose-1-phosphate adenylyltransferase
Gbem_R7073	3' of Gbem_1264<br>5' of Gbem_1265	aspartate kinase; citramalate synthase
Gbem_R7074<br>Gbem_R7075	3' of Gbem_1275<br>5' of Gbem_1276	histone-like protein; glycosyl transferase, group 2
Gbem_R7076<br>Gbem_R7077	3' of Gbem_1599<br>5' of Gbem_1600	3-deoxy-D-manno-octulosonate cytidylyltransferase; CTP synthase
Gbem_R7078<br>Gbem_R7079	3' of Gbem_1896<br>5' of Gbem_1897	aspartate carbamoyltransferase; dihydroorotase, multifunctional complex type
Gbem_R7080<br>Gbem_R7081<br>Gbem_R7082<br>Gbem_R7083	3' of Gbem_1899<br>5' of Gbem_1900	PflX-related radical SAM domain iron-sulfur cluster-binding oxidoreductase; carbamoyl-phosphate synthase, large subunit lipoprotein, glutamine-dependent
Gbem_R7084<br>Gbem_R7085	3' of Gbem_2287<br>5' of Gbem_2286	UDP-glucose 6-dehydrogenase; NAD-dependent nucleoside diphosphate-sugar epimerase/dehydratase
Gbem_R7086<br>Gbem_R7087	3' of Gbem_2298<br>5' of Gbem_2297	tryptophanyl-tRNA synthetase; chromosome segregation and condensation protein ScpA
Gbem_R7088<br>Gbem_R7089	3' of Gbem_2642<br>5' of Gbem_2641	peptide ABC transporter, membrane protein; conserved hypothetical protein
Gbem_R7090<br>Gbem_R7091<br>Gbem_R7092	3' of Gbem_2806<br>5' of Gbem_2805	sensor histidine kinase (PAS, HisKA, HATPase_c); sigma-54-dependent transcriptional response regulator (REC, sigma54 interaction, HTH8)
Gbem_R7093<br>Gbem_R7094<br>Gbem_R7095<br>Gbem_R7096	3' of Gbem_3342<br>5' of Gbem_3341	mannose-1-phosphate guanylyltransferase (truncated mannose-6-phosphate isomerase domain); aldehyde ferredoxin oxidoreductase, tungsten-containing
Gbem_R7097	3' of Gbem_3421<br>5' of Gbem_3420	NAD+ synthetase; SAM-dependent methyltransferase
Gbem_R7098	3' of Gbem_3451<br>5' of Gbem_3450	conserved hypothetical protein; undecaprenyl phosphate 4-deoxy-4-formamido-L-arabinose transferase, putative
Gbem_R7099	3' of Gbem_3921	NADH dehydrogenase I, F subunit; NADH

	5’ of Gbem_3920	dehydrogenase I, G subunit, putative
Gbem_R7100<br>Gbem_R7101<br>Gbem_R7102	3’ of Gbem_3971<br>5’ of Gbem_3970	metal-dependent hydrolase, beta-lactamase superfamily; FtsK/SpoIIIE domain protein
**Heptanucleotide repeats** - consensus [GGGGCTR]$_n$ (Figure S7)		
Gbem_R7103	3’ of Gbem_0407<br>5’ of Gbem_0408	glutamyl-tRNA reductase; hydroxymethylbilane synthase
Gbem_R7104	3’ of Gbem_0607<br>5’ of Gbem_0606	TPR domain protein; protein of unknown function UPF0047
Gbem_R7105	3’ of Gbem_1354<br>5’ of Gbem_1355	pyranopterin triphosphate synthase; molybdopterin sulfurtransferase C-terminal domain protein
Gbem_R7106<br>Gbem_R7107	3’ of Gbem_1531<br>5’ of Gbem_1532	valyl-tRNA synthetase; type IV prepilin-like proteins leader peptide processing enzyme
Gbem_R7108<br>Gbem_R7109<br>Gbem_R7110	3’ of Gbem_1590<br>5’ of Gbem_1591	iron/manganese-dependent transcriptional regulator; methyl-accepting chemotaxis sensory transducer
Gbem_R7111	3’ of Gbem_3533<br>5’ of Gbem_3532	divalent manganese/zinc ABC transporter, ATP-binding protein; divalent manganese/zinc ABC transporter, membrane protein
**23-nucleotide repeats** - consensus [TCTGCYTCACCTCTTAATCTCAA]$_4$ (Figure S7)		
Gbem_R7301	3’ of Gbem_0254<br>5’ of Gbem_0255	conserved hypothetical protein; SpoVR-like family protein
**21-nucleotide repeats** - consensus [ANRAAAANNWGGTCTTACAAA]$_4$ (Figure S7)		
Gbem_R7302	3’ of Gbem_0505<br>5’ of Gbem_0506	sodium/hydrogen exchanger; zinc-dependent oxidoreductase
**21-nucleotide repeats** - consensus [GTGCTGGYTACATACCGCGAT]$_{20}$ (Figure S7)		
Gbem_R7303	5’ of Gbem_0729<br>5’ of Gbem_0731	conserved hypothetical protein; response receiver phosphatase (REC, PP2C_SIG)
**20-nucleotide repeats** - consensus [AAYRTCACTTKSNSSNMAAA]$_{12}$ (Figure S7)		
Gbem_R7304	3’ of Gbem_1393<br>5’ of Gbem_1394	conserved hypothetical protein; entericidin superfamily protein
**21-nucleotide repeats** - consensus [AAYTCRYACAYCGCRRTATGT]$_{20}$ (Figure S7)		
Gbem_R7305	3’ of Gbem_1473<br>5’ of Gbem_1474	conserved hypothetical protein; helix-turn-helix transcriptional activator, AraC family
**21-nucleotide repeats** - consensus [TGMCYAAGTTGTTCGAGGACT]$_7$ (Figure S7)		
Gbem_R7306	3’ of Gbem_1903<br>3’ of Gbem_1905	ATP-dependent DNA helicase RecG; conserved hypothetical protein
**21-nucleotide repeats** - consensus [AAATCAAACTGATTYGGGGTC]$_{10}$ (Figure S7)		
Gbem_R7307	3’ of Gbem_2009<br>5’ of Gbem_2010	conserved hypothetical protein; conserved hypothetical protein
**21-nucleotide repeats** - consensus [MTGRSCGCGTKNYAATTTGTC]$_{19}$ (Figure S7)		
Gbem_R7308	3’ of Gbem_2054<br>5’ of Gbem_2053	lysyl-tRNA synthetase-related protein; biotin synthase
**21-nucleotide repeats** - consensus [SCGAAGCTCGTACCYNCGACT]$_{17}$ (Figure S7)		
Gbem_R7309	3’ of Gbem_2538	conserved hypothetical protein; conserved

	3’ of Gbem_2539	hypothetical protein
**21-nucleotide repeats** - consensus [ACGAAGCTCGYACCCRCGACT]$_{20}$ (Figure S7)		
Gbem_R7310	3’ of Gbem_2783<br>3’ of Gbem_2784	hypothetical protein; conserved hypothetical protein
**42-nucleotide repeats** - consensus [TGAAASGCGATGACGAATTGTCGGGAGATGGGGACGTTTAGY]$_{5}$ (Figure S7)		
Gbem_R7311	3’ of Gbem_3582<br>5’ of Gbem_3583	conserved hypothetical protein; conserved hypothetical protein
**42-nucleotide repeats** - consensus [CAYGCGCMCGTTYCAATTTGTCACGACCTCACGACAAATTAG]$_{5}$ (Figure S7)		
Gbem_R7312	3’ of Gbem_3673<br>5’ of Gbem_3672	conserved hypothetical protein; conserved hypothetical protein
**21-nucleotide repeats** - consensus [AAWTWRNCTAWTYSCRWWWSG]$_{17}$ (Figure S7)		
Gbem_R7313	3’ of Gbem_3913<br>5’ of Gbem_3912	NADH dehydrogenase I, N subunit; conserved hypothetical protein
**23-nucleotide repeats** - consensus [GGCTACGGCCGGCARGCCCCYAA]$_{7}$GGCTACG (Figure S7)		
Gbem_R7314	3’ of Gbem_3958<br>5’ of Gbem_3959	cytochrome *c*, 3 heme-binding sites; sensor protein (HAMP, PAS), putative heme-binding site
**21-nucleotide repeats** - consensus [NNTTCAAAWAYGWCATATTYG]$_{8}$ (Figure S7)		
Gbem_R7315	5’ of Gbem_3987<br>5’ of Gbem_3988	conserved hypothetical protein; peptidoglycan-binding ErfK/YbiS/YcfS/YnhG family lipoprotein
**Octanucleotide repeats** - consensus YTT[TCTTTGCG]$_{3}$TGAGG (Figure S8)		
Gbem_R8001<br>Gbem_R8002	3’ of Gbem_0412<br>5’ of Gbem_0413	ribonuclease Z; translation initation factor homolog YciH
Gbem_R8003<br>Gbem_R8004	3’ of Gbem_0427<br>5’ of Gbem_0426	conserved hypothetical protein; protein of unknown function DUF2495
Gbem_R8005<br>Gbem_R8006	3’ of Gbem_0764<br>5’ of Gbem_0765	inosine-5'-monophosphate dehydrogenase; guanosine monophosphate synthase
Gbem_R8007	3’ of Gbem_1600<br>5’ of Gbem_1601	CTP synthase; 3-deoxy-D-manno-octulosonic acid (KDO) 8-phosphate synthase
Gbem_R8008<br>Gbem_R8009<br>Gbem_R8010	3’ of Gbem_1601<br>5’ of Gbem_1602	3-deoxy-D-manno-octulosonic acid (KDO) 8-phosphate synthase; arabinose-5-phosphate isomerase
Gbem_R8011<br>Gbem_R8012	3’ of Gbem_1602<br>5’ of Gbem_1603	arabinose-5-phosphate isomerase; 3-deoxy-D-manno-octulosonate (KDO) 8-phosphate phosphatase
Gbem_R8013<br>Gbem_R8014	3’ of Gbem_1603<br>5’ of Gbem_1604	3-deoxy-D-manno-octulosonate (KDO) 8-phosphate phosphatase; periplasmic polysaccharide biosynthesis/export protein
Gbem_R8015<br>Gbem_R8016<br>Gbem_R8017<br>Gbem_R8018	5’ of Gbem_1632<br>5’ of Gbem_1633	conserved hypothetical protein; NAD-dependent nucleoside diphosphate-sugar epimerase/dehydratase
Gbem_R8019	3’ of Gbem_1633	NAD-dependent nucleoside diphosphate-sugar

	5' of Gbem_1634	epimerase/dehydratase; conserved hypothetical protein
Gbem_R8020	3' of Gbem_1634<br>5' of Gbem_1635	conserved hypothetical protein; conserved hypothetical protein
Gbem_R8021<br>Gbem_R8022	3' of Gbem_1636<br>5' of Gbem_1637	conserved hypothetical protein; conserved hypothetical protein
Gbem_R8023<br>Gbem_R8024	3' of Gbem_1637<br>5' of Gbem_1638	conserved hypothetical protein; ATPase, putative
Gbem_R8025<br>Gbem_R8026	3' of Gbem_1640<br>5' of Gbem_1641	conserved hypothetical protein; conserved hypothetical protein
Gbem_R8027<br>Gbem_R8028	3' of Gbem_1644<br>5' of Gbem_1643	serine *O*-acetyltransferase; MiaB-like tRNA modifying enzyme
Gbem_R8029<br>Gbem_R8030	3' of Gbem_1645<br>5' of Gbem_1644	tRNA (*N*6-dimethylallyl-A37)-dimethylallyltransferase; serine *O*-acetyltransferase
Gbem_R8031<br>Gbem_R8032	3' of Gbem_2320<br>5' of Gbem_2319	biotin operon repressor and biotin--acetyl-CoA carboxylase ligase; pantothenate kinase, type III
Gbem_R8033<br>Gbem_R8034	3' of Gbem_3207<br>3' of Gbem_3208	methionyl-tRNA synthetase; YdcF-like protein of unknown function DUF218
**Octanucleotide repeats** - consensus $[CCCGSCTT]_4$ (Figure S8)		
Gbem_R8035	5' of Gbem_2144<br>5' of Gbem_2145	YHS/TRASH domain protein; conserved hypothetical protein
**Nonanucleotide repeats** - consensus $TTT[WCCTCTGTGR_{0-1}Y_{0-1}]_nGTGAG$ (Figure S9)		
Gbem_R9001<br>Gbem_R9002	3' of Gbem_0075<br>5' of Gbem_0076	response regulator (REC, PilZ); ATP-dependent DNA helicase UvrD/REP
Gbem_R9003	3' of Gbem_0167<br>5' of Gbem_0166	conserved hypothetical protein; NADH dehydrogenase I, N subunit lipoprotein
Gbem_R9004	3' of Gbem_0168<br>5' of Gbem_0167	NADH dehydrogenase I, M subunit; conserved hypothetical protein
Gbem_R9005<br>Gbem_R9006	3' of Gbem_0170<br>5' of Gbem_0169	NADH dehydrogenase I, K subunit lipoprotein; NADH dehydrogenase I, L subunit lipoprotein
Gbem_R9007	3' of Gbem_0408<br>5' of Gbem_0409	hydroxymethylbilane synthase; uroporphyrinogen III *C*2,*C*7-methyltransferase and uroporphyrinogen III synthase
Gbem_R9008	3' of Gbem_0410<br>3' of Gbem_0411	porphobilinogen synthase; cupin superfamily barrel domain protein
Gbem_R9009<br>Gbem_R9010	3' of Gbem_0460<br>5' of Gbem_0461	pyruvate dehydrogenase complex E1 protein, beta subunit; pyruvate dehydrogenase complex, E2 protein, dihydrolipoamide acetyltransferase
Gbem_R9011<br>Gbem_R9012	3' of Gbem_0500<br>5' of Gbem_0499	DNA polymerase IV kappa; DNA polymerase III, alpha subunit
Gbem_R9013	3' of Gbem_0613<br>5' of Gbem_0614	5-methyltetrahydrofolate--homocysteine *S*-methyltransferase and 5,10-methylenetetrahydrofolate reductase; sulfite reductase, assimilatory-type
Gbem_R9014	3' of Gbem_0616	iron-sulfur cluster-binding oxidoreductase; zinc

	3' of Gbem_0617	finger transcriptional regulator, TraR/DksA family
Gbem_R9015	3' of Gbem_0831<br>5' of Gbem_0832	8-amino-7-oxononanoate synthase; biotin biosynthesis carboxylesterase BioH
Gbem_R9016	3' of Gbem_1032<br>3' of Gbem_1033	conserved hypothetical protein; iron-sulfur-oxygen hybrid cluster protein (prismane)
Gbem_R9017	3' of Gbem_1554<br>5' of Gbem_1555	chorismate synthase; shikimate kinase
Gbem_R9018<br>Gbem_R9019	3' of Gbem_1695<br>5' of Gbem_1696	tungstate ABC transporter, ATP-binding protein; RNA polymerase sigma factor
Gbem_R9020<br>Gbem_R9021<br>Gbem_R9022	3' of Gbem_1697<br>5' of Gbem_1698	conserved hypothetical protein; phosphonoacetate hydrolase
Gbem_R9023<br>Gbem_R9024	3' of Gbem_1893<br>3' of Gbem_1894	deoxyguanosine triphosphate triphosphohydrolase, putative; conserved hypothetical protein TIGR00253 (RNA-binding translation factor?)
Gbem_R9025	3' of Gbem_1906<br>5' of Gbem_1905	antitoxin (VapI); conserved hypothetical protein
Gbem_R9026	3' of Gbem_1928<br>5' of Gbem_1927	L-threonine aldolase, low-specificity; antitoxin YefM, prevent-host-death family
Gbem_R9027<br>Gbem_R9028	3' of Gbem_2021<br>5' of Gbem_2022	3'-to-5' exonuclease, putative; malate:quinone oxidoreductase superfamily protein of unknown function PRK11728
Gbem_R9029	5' of Gbem_4103<br>5' of Gbem_2037	4-oxalocrotonate tautomerase superfamily protein; putative hydrolase
Gbem_R9030<br>Gbem_R9031<br>Gbem_R9032	3' of Gbem_2043<br>3' of Gbem_2044	protein of unknown function DUF815; outer membrane transport protein, OMPP1/FadL/TodX family
Gbem_R9033	3' of Gbem_2104<br>5' of Gbem_2102	polyketide synthase; 3-oxoacyl-(acyl carrier protein) synthase III
Gbem_R9034	3' of Gbem_2193<br>5' of Gbem_2192	indolepyruvate ferredoxin oxidoreductase, beta subunit; phenylacetate-coenzyme A ligase
Gbem_R9035<br>Gbem_R9036	3' of Gbem_2219<br>3' of Gbem_2220	cystathionine beta-lyase; conserved hypothetical protein
Gbem_R9037	3' of Gbem_2328<br>5' of Gbem_2327	orotate phosphoribosyltransferase; conserved hypothetical protein
Gbem_R9038	3' of Gbem_2472<br>5' of Gbem_2473	*N*-carbamoylputrescine amidohydrolase; helicase, putative
Gbem_R9039<br>Gbem_R9040	3' of Gbem_2501<br>5' of Gbem_2500	oxidoreductase, aldo/keto reductase family; glycerol dehydratase-activating enzyme, putative
Gbem_R9041<br>Gbem_R9042	3' of Gbem_2503<br>3' of Gbem_2504	conserved hypothetical protein; inner membrane protein PRK10621
Gbem_R9043	3' of Gbem_2542<br>5' of Gbem_2543	predicted thioesterase PaaI; conserved hypothetical protein
Gbem_R9044	3' of Gbem_2641<br>5' of Gbem_2640	conserved hypothetical protein; tRNA(Ile) lysidine-34 synthase, putative

Gbem_R9045<br>Gbem_R9046	3' of Gbem_2670<br>5' of Gbem_4150	conserved hypothetical protein; hypothetical protein
Gbem_R9047<br>Gbem_R9048	3' of Gbem_2892<br>5' of Gbem_2891	4-hydroxy-3-methylbut-2-en-1-yl diphosphate synthase; prolyl-tRNA synthetase
Gbem_R9049<br>Gbem_R9050<br>Gbem_R9051<br>Gbem_R9052	3' of Gbem_2956<br>5' of Gbem_2958	peroxiredoxin, 1-Cys subfamily, selenocysteine-containing; FAD-dependent oxidoreductase, phytoene dehydrogenase family
Gbem_R9053<br>Gbem_R9054	3' of Gbem_3018<br>3' of Gbem_3019	conserved hypothetical protein; homoserine dehydrogenase
Gbem_R9055	5' of Gbem_3216<br>5' of Gbem_3217	conserved hypothetical protein; conserved hypothetical protein
Gbem_R9056<br>Gbem_R9057	3' of Gbem_3311<br>5' of Gbem_3310	zinc-dependent oxidoreductase; membrane protein, MarC family
Gbem_R9058	3' of Gbem_3376<br>5' of Gbem_3375	glycerol kinase; FAD-dependent glycerol-3-phosphate dehydrogenase
Gbem_R9059<br>Gbem_R9060<br>Gbem_R9061	3' of Gbem_3561<br>5' of Gbem_3562	NAD-dependent nucleoside diphosphate-sugar epimerase/dehydratase; metal-binding domain transcriptional regulator
Gbem_R9062	3' of Gbem_3648<br>3' of Gbem_3649	thioesterase family protein; lipoprotein signal peptidase
Gbem_R9063<br>Gbem_R9064<br>Gbem_R9065	3' of Gbem_3770<br>3' of Gbem_3771	ATP-dependent DNA helicase DinG; putative amidophosphoribosyltransferase
Gbem_R9066	5' of Gbem_3974<br>5' of Gbem_3975	[acyl-]glycerolphosphate acyltransferase; single-strand binding protein
Gbem_R9067<br>Gbem_R9068	3' of Gbem_4051<br>3' of Gbem_4052	intracellular protease, PfpI family; L,L-diaminopimelate aminotransferase
**Nonanucleotide repeats** - consensus AAARRCNTTTA$[MMRGGGATR]_3$AMWNCRAAAAM (Figure S9)		
Gbem_R9069	3' of Gbem_0410<br>3' of Gbem_0411	porphobilinogen synthase; cupin superfamily barrel domain protein
Gbem_R9070	3' of Gbem_0961<br>5' of Gbem_0962	ribosomal protein L17; chromosome segregation ATPase SMC
Gbem_R9071<br>Gbem_R9072<br>Gbem_R9073	3' of Gbem_1600<br>5' of Gbem_1601	CTP synthase; 3-deoxy-D-manno-octulosonic acid (KDO) 8-phosphate synthase
Gbem_R9074<br>Gbem_R9075	5' of Gbem_1632<br>5' of Gbem_1633	conserved hypothetical protein; NAD-dependent nucleoside diphosphate-sugar epimerase/dehydratase
Gbem_R9076	3' of Gbem_1637<br>5' of Gbem_1638	conserved hypothetical protein; ATPase, putative
Gbem_R9077	3' of Gbem_1644<br>5' of Gbem_1643	serine *O*-acetyltransferase; MiaB-like tRNA modifying enzyme
Gbem_R9078	3' of Gbem_1645<br>5' of Gbem_1644	tRNA (*N*6-dimethylallyl-A37)-dimethylallyltransferase; serine *O*-acetyltransferase

Gbem_R9079	3’ of Gbem_1961<br>3’ of Gbem_1962	D-3-phosphoglycerate dehydrogenase; 3-deoxy-D-arabino-heptulosonate 7-phosphate (DAHP) synthase
Gbem_R9080	3’ of Gbem_2193<br>5’ of Gbem_2192	indolepyruvate ferredoxin oxidoreductase, beta subunit; phenylacetate-coenzyme A ligase
Gbem_R9081	3’ of Gbem_2503<br>3’ of Gbem_2504	conserved hypothetical protein; inner membrane protein PRK10621
Gbem_R9082	3’ of Gbem_2542<br>5’ of Gbem_2543	predicted thioesterase PaaI; conserved hypothetical protein
Gbem_R9083	5’ of Gbem_2860<br>5’ of Gbem_2861	conserved hypothetical protein; CsbD family protein
Gbem_R9084	3’ of Gbem_2950<br>3’ of Gbem_2951	sensor histidine kinase response regulator (PAS, HisKA, HATPase_c, REC); sensor histidine kinase (HAMP, PAS, HisKA, HATPase_c)
Gbem_R9085	3’ of Gbem_2958<br>3’ of Gbem_2959	FAD-dependent oxidoreductase, phytoene dehydrogenase family; oxidoreductase, short-chain dehydrogenase/reductase family
Gbem_R9086<br>Gbem_R9087	3’ of Gbem_3095<br>3’ of Gbem_3096	pirin family protein; histidyl-tRNA synthetase
Gbem_R9088	3’ of Gbem_3376<br>5’ of Gbem_3375	glycerol kinase; FAD-dependent glycerol-3-phosphate dehydrogenase
Gbem_R9089<br>Gbem_R9090	3’ of Gbem_3459<br>5’ of Gbem_3458	conserved hypothetical protein; phosphoribosylaminoimidazolecarboxamide formyltransferase/IMP cyclohydrolase
**Nonanucleotide repeats** - consensus [WYGGACCTG]$_{4\text{-}19}$ (Figure S9)		
Gbem_R9091	3’ of Gbem_1012<br>3’ of Gbem_1013	cell division ATP-dependent zinc protease FtsH; conserved hypothetical protein
Gbem_R9092	3’ of Gbem_1586<br>5’ of Gbem_1587	conserved hypothetical protein; ferric uptake regulation protein Fur
Gbem_R9093	3’ of Gbem_1590<br>5’ of Gbem_1591	iron/manganese-dependent transcriptional regulator; methyl-accepting chemotaxis sensory transducer
Gbem_R9094	3’ of Gbem_1950<br>5’ of Gbem_1952	RarD protein, DMT superfamily transporter; response receiver histidine kinase response regulator (REC, HisKA, HATPase_c, REC)
Gbem_R9095	3’ of Gbem_2656<br>5’ of Gbem_2655	UbiD family decarboxylase; putative rRNA methyltransferase, YqxC-related
Gbem_R9096	3’ of Gbem_3751<br>5’ of Gbem_3749	chemotaxis MotB protein; conserved hypothetical protein
Gbem_R9097	3’ of Gbem_1294<br>3’ of Gbem_1295	aconitate hydratase 2; oligoendopeptidase, pepF/M3 family
