# Supplementary material for: The genome of Geobacter bemidjiensis, exemplar for the subsurface clade of Geobacter species that predominate in Fe(III)-reducing subsurface environments
Source: BMC Genomics. 2010 Sep 9;11:490. doi: 10.1186/1471-2164-11-490 (PMC2996986; doi:10.1186/1471-2164-11-490)
Supplement: Additional file 9 — Figure S3. Multicopy nucleotide sequences of the G. bemidjiensis genome: base coordinates and alignments. (See also Table S6.). [file 1471-2164-11-490-S9.PDF]

Gbem_R3001	+	382536	382659	GATATACGACAA-TACTAAACCTTCCGCGAGGATGGGACGGAAAGCCTATAGGGTCTCACTGAGACAGCCGGGTCGCCGAAAATATCTTTTTC----GATATT-------------AAAGGTGGCCCGGCCTTTTTTTGTTC
Gbem_R3002	-	649617	649733	GATACACGATAA-TACTCAACCATCCGCGAGGATGGGGCGGAAAGCCTATAGGGTCTCCCTGAGACAGCCGGGTTGCCGAA-ATATCCTTA------GATAT---------------TCTGCG-CCCGGCTTT-TTTGCGTT
Gbem_R3003	-	1255931	1256050	GATACACGACAA-TACTAAAACTTTCGCGAGAAAGTGACGGAAAGCCTATTGGGTCTCACGAAGACAGCCGGGTCGCCGAA-ATATCGAAACCAAC-GATAT----------------CG--GACCCGGCTTT-TTTGCGTC
Gbem_R3004	+	1375448	1375570	GATAAACGATAA-TACTAAACCATCCGCGAGGATGGGGCGGAAAGCCTACAGGGTCTCACAGAGACAGCCGGGTTGCCGAA-ATATCTCTGACCA--GATA---------------GCCGGCAACCCGTTTTTTTTAGCCCA
Gbem_R3005	+	2076754	2076883	CAACAACGACAA-TACTAAACCACCTGCGAAGGTGGGACGGAAAGCCCAC-GGGTCTCCCTGAGACAGCCGGGTCGCCGA--TTCT--ACGC-----GATGGAACCCCCATCGTAGACCGGCGACCCGGCTTT-TTTTTGTA
Gbem_R3006	+	2079947	2080049	CAACAACGACAA-TACTAAACCATCCGTGAGGATGGGACGGAAAGCCCAC-GGGTCTCCCTGAGACAGCCGGGTCGCCGA--ATCT-AACA------GAT---------------------------GGTTTT-TTTACATC
Gbem_R3007	-	2178881	2179004	GGTAGACGACAAATGCTAAACCATCCGCGAGGATGGGGCGGAAAGCCCA-AGGGTCTCACCGAGACAGCCGGGTTGCCGAA-ATATCACGTGAGTTAGATAT--------------GACG-CAGCGCGGTTTT-TTTGTCCC
Gbem_R3008	-	2179073	2179191	GGTAGACGACAA-TACTAAACCATCCGCGAGGGTGGGACGGAAAGCCTACAGGGTCTCCACGAGACAGCCGGGTCGCCGAA-ATATCAACTAC----GATATT--------------CCTGC--CCCGGCTTT-TTTGCGTC
Gbem_R3009	-	2499028	2499144	GATACACGATAA-TACTCAACCACTCGCGAGGGTGGGGCGGAAAGCCTACAGGGTCTCCTTGAGACAGCCGGGTTGCCGAA-ATATCAACGC-----GATAT---------------TCAGC--CCCGGCTTT-TTTGCGTC
Gbem_R3010	-	2624770	2624881	ACAAAGTTATAA-TACTAACCCATCCGTGAGGATGTGATGGAAGGCCTAGTGGGTCTCACAGAGGCAGATGGGGCGC-GGA--TGTT----------CATATT-------------TTGGGCT-CCCG-CTTG-TTTGTCTG
Gbem_R3011	+	3197826	3197944	GATAGACGACAA-TACTAAACCATCCGCGAGGATGGGACGGAAAGCCTACAGGGTCTCCAAGAGACAGCCGGGTCGCCGAA-ATATCGATC------GATATT-------------TTTGGC--CCCGGCTTTTTTGTTGCC
Gbem_R3012	-	3434265	3434382	AGATAACGACAA-TACTAAACCATCCGCGAGGGTGGGACGGAAAGCCTACTGGGTCTCATAGAGACAGCCGGGTCGCCGAA-ATATCTCAA------GATATT--------------CTGGC--CCCGGCTTTTTTTGTGCC
Gbem_R3013	+	4525105	4525220	GATAGACGATAA-TACTCAACCATCCGCGAGGATGGGGCGGAAAGCCTATAGGGTCTCACCGAGACAGCCGGGTTGCCGAA-ATATC-ACA------GATAT---------------TCGGT--CCCGGCTTTTTTTGTGTC
Gbem_R3014	+	4525291	4525407	GGTACACGATAA-TACTCAACCATCCGCGAGGATGGGGCGGAAAGCCTATTGGGTCTCTCTGAGACAGCCGGGTTGCCGAA-ATATC-ACAC-----GATAT----------------CGGC-TCCCGGCTTTTTTTGTGCC
Consensus				GRWANACGAYAA TACTMAACCATCCGCGAGGRTGGGRCGGAAAGCCTAYWGGGTCTCMCWGAGACAGCCGGGTYGCCGAA ATATC AYN GATAT CGGC CCCGGCTTT TTTGYGYC
