# Supplementary material for: The genome of Geobacter bemidjiensis, exemplar for the subsurface clade of Geobacter species that predominate in Fe(III)-reducing subsurface environments
Source: BMC Genomics. 2010 Sep 9;11:490. doi: 10.1186/1471-2164-11-490 (PMC2996986; doi:10.1186/1471-2164-11-490)
Supplement: Additional file 10 — Figure S4. Multicopy nucleotide sequences of the G. bemidjiensis genome: base coordinates and alignments. (See also Table S6.). [file 1471-2164-11-490-S10.PDF]

Gbem_R4001	-	22802	22836	CCCTCA-CCCT----------GTCCCTCTCCCAGAGGGACAGGGGA
Gbem_R4002	-	34095	34130	CCCTCACCCCA----------GCCCCTCTCCCAGAGGGCGAGGGGT
Gbem_R4003	+	79368	79403	CCCTCACCCCC----------GCCCCTCTCCCAGAGGGCGAGGGGA
Gbem_R4004	+	93508	93542	CCCTCA-CCCC----------AACCCTCTCCCAGAGGCAGAGGGAG
Gbem_R4005	-	140843	140886	CGTTCACCCGGCCTT--TGGCCACCCTCGCCCGTGCGGCGAGTTGA
Gbem_R4006	+	212153	212188	CCCTCACCCCC----------GCCCCTCTCCCAAAGGGCGAGGGGA
Gbem_R4007	-	379320	379363	GGCAAGCCCGCCCTT--CGGGCACCCTCTCCCAAAGGACGAGGGGA
Gbem_R4008	+	405670	405707	CCCTCGCCCTGT--------GGGCCTTCGCCCACCGTAGGCTTCGG
Gbem_R4009	-	405703	405738	CCCTCACCCCC----------GCCCCTCTCCCACCTTCGGACCGAA
Gbem_R4010	+	413228	413262	CCCTCACCCCG-----------ACCCTCTCCCAGAGGGAGAGGGGG
Gbem_R4011	-	420394	420429	CCCTCACCCCC----------TCCCCTCTCCCAGAGGGCGAGGGGC
Gbem_R4012	-	710420	710454	CCCTCACCCCG-----------ACCCTCTCCCAAGGGGAGAGGGAG
Gbem_R4013	-	798944	798987	CCCTCACCCGGCCTT--CGGCCACCCTCTCCCAGAGGGAGAGGGGA
Gbem_R4014	-	890017	890061	CCCTCACCCGGCCTT-GCGGCCACCCTCTCCCAGAGGGTGAGGGGA
Gbem_R4015	+	890086	890120	CCCTCACCCCG-----------ACCCTCTCCCAGAGGGAGAGGGAG
Gbem_R4016	-	1121925	1121959	CCCTCACCCTG-----------TCCCTCTCCCGCCGGGCGACGGGA
Gbem_R4017	-	1213485	1213519	CCCTCACCCCG-----------CCCCTCTCCCAGAGGGAGAGGGGG
Gbem_R4018	+	1443382	1443417	CCCTCACCCCC----------GCCCCTCTCCCACCTGTGGACCGTA
Gbem_R4019	-	1443413	1443451	CCCTCGCCCCTCT-------GGGCCTTCGCCCACCGTAGGCTACGG
Gbem_R4020	+	1565599	1565633	CCCTCACCCTA-----------ACCCTCTCCCAGAGGGAGAGGGGA
Gbem_R4021	-	1565661	1565706	CCCTCATCCGCCCCTCCGGGGCACCTTCTCCCAGAGGGAGAAGGGA
Gbem_R4022	+	1565735	1565769	CCCTCACCCTG-----------ACCCTCTCCCGGAGGGAGAGGGGA
Gbem_R4023	+	1640424	1640458	CCCTCACCCCA-----------ACCCTCTCCCAGAGGGAGAGGGGG
Gbem_R4024	-	1653295	1653330	CCCTCACCCCC----------GCCCCTCTCCCAGAGGGCGAGGGGG
Gbem_R4025	-	1677505	1677539	CCCTCACCCCG-----------GCCCTCTCCCAGAGGGAGAGGGAG
Gbem_R4026	+	1679753	1679787	CCCTCACCCTG-----------ACCCTCTCCCAAAGGGAGAGGGGA
Gbem_R4027	-	1679833	1679876	CCCTCACCCGCCCTT--CGGGCACCCTCTCCCAGAGGGAGAGGGTT
Gbem_R4028	+	1692535	1692570	CCCTCACCCCC----------GCCCCTCTCCCAGAGGGCGAGGGGA
Gbem_R4029	-	1692598	1692641	CCCTCACCCGGCCTT--CGGCCACCCTCTCCCAGAGGGAGAGGGGA
Gbem_R4030	+	1705094	1705131	CCCTCTCCCTCT--------GGGCCTTCGCCCACCGTAGGCTACGG
Gbem_R4031	-	1705127	1705161	CCCTCACCCCG-----------ACCCTCTCCCACCTGTGGACCGTA
Gbem_R4032	+	1983667	1983701	CCCTCACCCCG-----------ACCCTCTCCCAGAGGGAGAGGGGG
Gbem_R4033	+	1996364	1996397	CCCTCACCCCC-----------GCCC-CTCCCACCTGTGGACCGTA
Gbem_R4034	-	1996393	1996430	CCCTCGCCCTCT--------GGGCCTTCGCCCACCGTAGGCTACGG
Gbem_R4035	-	2349219	2349255	CCCTCACCCACT---------CTCCCTCTCCCAGGGGGGAGAGGGA
Gbem_R4036	-	2349328	2349362	CCCTCACCCCG-----------ACCCTCTCCCAAAGGGAGAGGGAG
Gbem_R4037	-	2511317	2511351	CCCTCACCCAG-----------ACCCTCTCCCGCCAACGGCATCGC
Gbem_R4038	+	2527010	2527044	CCCTCACCCCG-----------ACCCTCTCCCATAGGGCGAGGGAG
Gbem_R4039	-	2527060	2527097	CCCGCCCTGCGG--------GCACCCTCTCCCAGTGGGAGAGGGGA
Gbem_R4040	-	2565526	2565560	CCCTCACCCCG-----------ACCCTCTCCCATAGGGAGAGGGAG
Gbem_R4041	+	2633578	2633613	CCCTCACCCCT----------GCCCCCCTCCCAGAGGGAGAGGGGC
Gbem_R4042	-	2753916	2753951	CCCTCACCCCC----------GCCCCTCTCCCAAAGGGCGAGGGGA
Gbem_R4043	-	2897069	2897103	CCCTCACCCCA-----------ACCCTCTCCCAGAGGGAGTGGGGG
Gbem_R4044	+	2964784	2964827	CCCTCACCCGCCCTT--CGGGCACCCTCTCCCAGAGGGAGAGGGGA
Gbem_R4045	-	3035679	3035713	CCCTCACCCCA-----------ACCCTCTCCCAGAGGGAGAGGGGG
Gbem_R4046	+	3035737	3035780	CCCTCACCCGACCTT--CGGCCACCCTCCCCCAGGGGAGGAGGGTA
Gbem_R4047	-	3087198	3087241	CCCTCACCCGCCCTT--CGGGCACCCTCTCCCATCTCAAGCTCCAG
Gbem_R4048	+	3167646	3167680	CCCTCACCCCA-----------ACCCTCTCCCAAAGGGAGAGGGAG
Gbem_R4049	-	3187369	3187403	CCCTCACCCCG-----------GCCCTCTCCCGGAGGGCGAGGGGG
Gbem_R4050	-	3201454	3201489	CCCTCACCCCC----------GCCCTTCTCCCAGAGGGCGAGGGGA
Gbem_R4051	-	3249401	3249436	CCCTCACCCCC----------GCCCCTCTCCCAAAGGGCGAGGGGA
Gbem_R4052	+	3332517	3332552	CCCTCACCCCT----------GCCGCTCTCCCAGAGGGCGAGGGGA
Gbem_R4053	+	3484550	3484593	CCCTCACCCGGCCTG--CGGCCACCCTCTCCCACAGGGAGAGGGGT
Gbem_R4054	-	3713734	3713768	CCCTCACTCCG-----------GCCCTCTCCCAGAGGGAGCCTGAC
Gbem_R4055	-	3713829	3713863	CCCTCACCCCG-----------ACCCTCTCCCAGAGGGAGAGGGAG
Gbem_R4056	+	3799029	3799063	CCCTCACCCCG-----------CCCCTCTCCCAGAGGGCGAGGGGA
Gbem_R4057	-	3799084	3799127	CCCTCACCCGGCCTT--CGGCCACCCTCTCCCAGAGGGAGAGGGTA
Gbem_R4058	-	3804741	3804775	CCCTCACCCCG-----------ACCCTCTCCTAGGGGGAGAGGGAG
Gbem_R4059	+	3890761	3890795	CCCTCACCCTA-----------ACCCTCTCCCAGAGGGAGAGGGGA
Gbem_R4060	+	4051126	4051165	ATTCCACCTCTCC------AGTCCCCCCTCCTCGCGGGAGGGAGAG
Gbem_R4061	-	4051212	4051246	CCCTCACCCCA-----------ACCCTCTCCCAGAGGGAGAGGGGG
Gbem_R4062	+	4051270	4051313	CCCTCACCCGGCCTT--CGGCCACCCTCCCCCCCTGGGGGAGGGTA
Gbem_R4063	+	4054671	4054706	CCCTCACCCCC----------GCCCCTCTCCCACCTGTGGACCGAA
Gbem_R4064	-	4054702	4054739	CCCTCGCCCTAT--------GGGCCTTCGCCCACCGTAGGCTTCGG
Gbem_R4065	-	4054801	4054835	CCCTCACCCCG-----------GCCCTCTCCCAGAGGGAGAGGGAG
Gbem_R4066	+	4154457	4154491	CCCTCACCCCA-----------ACCCTCTCCCAGGGGGAGAGGGAG
Gbem_R4067	-	4227835	4227869	CCCTCACCCCG-----------GCCCTTTCCCAGAGGGCGAGGGAG
Gbem_R4068	+	4228557	4228591	CCCTCACCCCG-----------GCCCTCTCCCAAGGGGAGAGGGAG
Gbem_R4069	+	4339245	4339279	CCCTCACCCCG-----------GCCCTCTCCCACCTCCGGACCGTA
Gbem_R4070	-	4339275	4339312	CCCTCTCCCTCT--------GGGCCTTCGCCCACCGTAGGCTACGG
Gbem_R4071	-	4339338	4339381	CCCTCACCCGGCCTG--CGGCCACCCTCTCCCAGAGGGCGAGGGTA
Gbem_R4072	+	4348790	4348825	CCCTCACCCCC----------GCCCCTCTCCCACCTTCGGACCGGA
Gbem_R4073	-	4348822	4348859	CCCTCGCCCTTC--------GGGCCTTCGCCCACCGTAGGCTTCCG
Gbem_R4074	+	4533139	4533174	CCCTCACCCCC----------GCCCCTCTCCCAAAGGGCGAGGGGA
Gbem_R4075	+	4549511	4549545	CCCTCACCCCG-----------GCCCTCTCCCAGAGGGAGAGGGAG
Gbem_R4076	+	4569299	4569333	CCCTCACCCCA-----------ACCCTCTCCCAGAGGGAGAGGGGG
Consensus				CCCTCACCCCS SMCCCTCTCCCASMGGGNGAGGGRR
