# Supplementary material for: The genome of Geobacter bemidjiensis, exemplar for the subsurface clade of Geobacter species that predominate in Fe(III)-reducing subsurface environments
Source: BMC Genomics. 2010 Sep 9;11:490. doi: 10.1186/1471-2164-11-490 (PMC2996986; doi:10.1186/1471-2164-11-490)
Supplement: Additional file 12 — Figure S6. Multicopy nucleotide sequences of the G. bemidjiensis genome: base coordinates and alignments. (See also Table S6.). [file 1471-2164-11-490-S12.PDF]

Gbem_R6001	+	2964	2996	T C T T A A T C T C A A T C T C A A T C T C A A T C T T A A T C T
Gbem_R6002	+	12226	12252	T C T C A A T C T T A A T C T T A A T C T T T A T C T
Gbem_R6003	-	323309	323335	T C T T T A T C T C T A T C T C T A T C T T T A T C T
Gbem_R6004	-	323366	323392	C C T C A A T C T C T A T C T C T A T C T T T A T C T
Gbem_R6005	-	344606	344620	T C T T A A T C T T A A T C T
Gbem_R6006	+	344646	344672	T C T T G A T C T C A A T C T T A A T C T T A A T C T
Gbem_R6007	-	594004	594030	T C T T T A T C T T T A T C T T T A T C T T T A T C T
Gbem_R6008	+	716195	716221	C C T C A A T C T T T A T C T T T A T C T T T A T C T
Gbem_R6009	-	740837	740863	C C T T T A T C T T T A T C T T T A T C T C T A T C T
Gbem_R6010	+	740991	741017	C C T C A A T C T T T A T C T C T A T C T C T A T C T
Gbem_R6011	+	997839	997871	T C T C A A T C T T A A T C T T A A T C T T A A T C T C A A T C T
Gbem_R6012	+	1519044	1519070	C C T C A A C C T T A A C C T T A A C C T T A G C C T
Gbem_R6014	+	1769522	1769554	G C T T T A C C T T T A T C T T T A T C T C T A T C T T T A T C T
Gbem_R6015	+	1810747	1810785	T C T T A G T C T A A A A C T T A A T C T T A G T C T T A A T C T T A A T C T
Gbem_R6016	-	2404389	2404415	C C T T T A T C T A A A T C T T A A T C T C A A T C T
Gbem_R6017	+	2404439	2404465	T C T T T A T C T C T A T C T T T A T C T T T A T C T
Gbem_R6018	+	2545932	2545958	T C T C A A T C T T T A T C T T T A T C T T T A T C T
Gbem_R6019	+	2604740	2604772	T C T C T G T C T C A G T C T T A A T C T C A G T C T C A A T C T
Gbem_R6020	+	2604793	2604825	C C T T A A T C T C A A T C T T A A T C T C A A T C A T T A T C T
Gbem_R6021	-	2708652	2708708	T C T T T A T C T T T A T C T T T A T C T T T A T C T C G A T C T T A A T C T C A A T C T T A A T C T T A A T C T
Gbem_R6022	-	2714365	2714415	T C T T A A T C T T A A T C T T A G T C T T A A T C T C C A T C T T T A T C T T T A T C T T T A T C T
Gbem_R6023	+	2714501	2714533	C C T T A A T C T T T A T C T T T A T C T T T A T C T C A A T G T
Gbem_R6024	-	3445173	3445205	T C T C A A T C T C A A T C T T A A T C T T A A T C T C A A T T T
Gbem_R6025	-	3489536	3489568	T C T T A A C C T C A G T C T T A A T C T T A A T C T T A A T C T
Gbem_R6026	-	3615819	3615845	T C T T A A T C T T A A T C T T A A T C T T T A T C T
Gbem_R6028	+	4066004	4066030	A C T T T A T C T T T A T C T T T A T C T C A A T C T
Gbem_R6029	-	4151530	4151568	A C T T A A T C T C A A T C T C A A T C T C A A T C T C A A T C T C G A T C T
Gbem_R6030	-	4226918	4226962	T C T C A A A C T T A G T C T T A A T C T C A A T C T T A A T C T C A A T C T T A A T C T
Gbem_R6031	-	4378266	4378292	T C T T A A T C T C A A T C T C A A T C T T G A T C T
Gbem_R6032	+	4378328	4378360	C C T C A A T C T T A A T C T T A A T C T C A A T C T C A A T C T
Gbem_R6033	-	4533211	4533237	G C T T A A T C T C A A T C T C T A T C T T A A T C T
Gbem_R6034	+	4533310	4533396	T C C T T A T C T T A G A C T C A A T C T C A A T C T T A A T C T T A G T C T C A A T C T T T A T C T T T A T C T C A A T C T T T A T C T T T A T C T C A A T C T T T A T C T
Consensus				T C T Y W A T C T Y W A T C T Y W A T C T Y W A T C T Y W A T C T Y W A T C T Y W A T C T Y W A T C T Y W A T C T Y W A T C T Y W A T C T Y W A T C T Y W A T C T Y W A T C T
Gbem_R6013	-	1589927	1590010	G T G G C T G T G T C T G T G C C T G T G T T T G C G T C T G T G T T T G C G T C T G C G G T T G T G T T T G C G G C T G C G T C T G C G G T T G T G C C T G G G T C T
Gbem_R6027	-	4031034	4031069	G G G T A A G G G T C A G G G T C A G G G T C A G G G T C A G G G T C A
