# Supplementary material for: The genome of Geobacter bemidjiensis, exemplar for the subsurface clade of Geobacter species that predominate in Fe(III)-reducing subsurface environments
Source: BMC Genomics. 2010 Sep 9;11:490. doi: 10.1186/1471-2164-11-490 (PMC2996986; doi:10.1186/1471-2164-11-490)
Supplement: Additional file 14 — Figure S8. Multicopy nucleotide sequences of the G. bemidjiensis genome: base coordinates and alignments. (See also Table S6.). [file 1471-2164-11-490-S14.PDF]

Gbem_R8001	-	489624	489655	T T T T C T T T G C G - C C T T T G C G G C T T T G C G T G A A T
Gbem_R8002	+	489664	489695	T T T G C T T T G C G - C C T T T G C G G C T T T G C G T G A G A
Gbem_R8003	-	502740	502771	A T T T C T T T G C G - T C T T T G C G G C T T T G C G T G A G A
Gbem_R8004	+	502788	502818	C G T T C T T C G C G - C C T T G G C G T C T - T G A G C G A G C
Gbem_R8005	-	900918	900949	T T T T C T C T G C G - T C T T T G C G G C T T T G C G T G A G C
Gbem_R8006	+	900961	900992	A G T A C T T T G C G - C C T T G G C G T C T T T G C G T G A C A
Gbem_R8007	-	1855979	1856010	C T T T C T T T G C G - T C T T T G C G G C T T T G C G T G A C A
Gbem_R8008	-	1857154	1857185	C T T T C T T T G C G - C C T T T G C G T C T T T G C G T G A G A
Gbem_R8009	-	1857257	1857288	C T T T C T T T G C G - T C T T T G C G G C T T G G C G T G A C A
Gbem_R8010	+	1857322	1857354	T T T T C T T T G C G G T C T T A G C G T C T T T G C G T G A G G
Gbem_R8011	-	1858398	1858429	C T T T C T T T G C G - T C T T T G C G G C T T T G C G T G A C C
Gbem_R8012	+	1858461	1858493	T T T T C T T T G C G G T C T T G G C G T C T T T G C G T G A G A
Gbem_R8013	-	1859057	1859088	C T T T C T T T G C G - T C T T T G C G G C T T T G C G T G A C A
Gbem_R8014	+	1859205	1859237	T G T T C T T T G C G T T C T C T G C G T C T T T G C G T G A G G
Gbem_R8015	-	1895875	1895907	C T T T C T T T G C G G T C T T C G C G G C T T T G C G T G A G A
Gbem_R8016	-	1895926	1895957	T T T G C T T T G C G - C C T C T G C G G C T T T G C G T G A G G
Gbem_R8017	-	1896021	1896052	C T T T C T T C G C G - T C T T T G C G C C T T T G C G T G A G A
Gbem_R8018	+	1896118	1896148	C G T T C T T C G C G - C C T T T G C G G C T - T G A G T G A G C
Gbem_R8019	-	1898312	1898343	C T T T C T T T G C G - T C T T T G C G G C T T T G C G T G A G T
Gbem_R8020	+	1898770	1898801	T A C C C T T T G C G - C C T T T G C G G C T T T G C G T G A G A
Gbem_R8021	-	1902188	1902219	C T T T C T C C G C G - T C T T T G C G G C T T T G C G T G A G A
Gbem_R8022	+	1902230	1902261	T C T C C T T T G C G - C C T T T G C G T C T T T G C G T G A C A
Gbem_R8023	-	1902603	1902634	T T T T C T T G G C G - T C T T T G C G G C T T T G C G T G A G A
Gbem_R8024	+	1902763	1902795	T T T C C T T T G C G G T C T T T G C G T C T T T G C G T G A G G
Gbem_R8025	-	1904879	1904910	T T T T C T T G G C G - T C T T T G C G G C T T T G C G T G A G G
Gbem_R8026	+	1904931	1904963	T T T A C T T T G C G T G C T T T G C G G C T T T G C G T G A C A
Gbem_R8027	-	1909030	1909061	T C G T C T T T G C G - T C T T T G C G G C T T T G C G T G A G A
Gbem_R8028	-	1909069	1909100	T T T T C T T T G C G - T C T T T G C G T C T T T G C G T G A G A
Gbem_R8029	-	1909995	1910025	T C T A C T T T G C G - T T T T T G C G G C T - T G A G T G A G C
Gbem_R8030	-	1910071	1910101	T C T A C T T T G C G - T C T T T G C G G C T - T G A G T G A G C
Gbem_R8031	-	2693405	2693436	T T T G C T T T G C G - C C T T T G C G G C T T T G C G T G A G G
Gbem_R8032	+	2693465	2693496	C T T T C T T T G C G - C C T T C G C G G C T T T G C G T G A G G
Gbem_R8033	-	3698749	3698780	C T T T C T T T G C G - T C T T C G C G T C T T T G C G T G A A C
Gbem_R8034	+	3698833	3698864	A G T T C T T T G C G - C C T T T G C G T C T T T G C G T G A G A
Consensus				Y T T T C T T T G C G T C T T T G C G T C T T T G C G T G A G G
Gbem_R8035	+	2497105	2497136	C C C G C C T T C C C G G C T T C C C G G C T T C C C G G C T T
