# Supplementary material for: The genome of Geobacter bemidjiensis, exemplar for the subsurface clade of Geobacter species that predominate in Fe(III)-reducing subsurface environments
Source: BMC Genomics. 2010 Sep 9;11:490. doi: 10.1186/1471-2164-11-490 (PMC2996986; doi:10.1186/1471-2164-11-490)
Supplement: Additional file 7 — Figure S1. Multicopy nucleotide sequences of the G. bemidjiensis genome: base coordinates and alignments. (See also Table S6.). [file 1471-2164-11-490-S7.PDF]

Gbem_R1001	+	26098	26150	AAAAAAGGGGACAGGCTACTTTTTGATTTGAAA-AAGTAGCCTGTCCCCCTTTC
Gbem_R1002	-	49601	49653	TAAAAGGGGGACAGGCTACTTTATTTTTTTAAA-AAGTAGCCTGTCCCCTTTTC
Gbem_R1003	-	49727	49779	AAAAAGGGGGACAGGCTACTTTATTTTTCGAAA-AAGTAGCCTGTCCCCTTTTT
Gbem_R1004	+	60248	60300	GATAAGGGGGACAGGCTACTTTTTTATTGGAAA-AAGTAGCCTGTCCCCTTTTC
Gbem_R1005	+	104983	105035	AGAAAAGGGGACAGGCTACTTTTTGATTCGAAA-AAGTAGCCTGTCCCCCTTTT
Gbem_R1006	-	105162	105214	AAAAAAGGGGACAGGCTACTTTTTCAGATGGAA-AAGTAGCCTGTCCCCTTTTA
Gbem_R1007	-	169543	169594	TAAAAAGGGGACAGGCTGCTTTTTGATTGAAA--AAGTAGCCTGTCCCCTTTTG
Gbem_R1008	-	308076	308128	AAAAAGGGGGACAGGCTACTTTTTCAAATCAAA-AAGTAGCCTGTCCCCTTTTT
Gbem_R1009	+	352750	352802	AAAAAGGGGGACAGGCTACTTTTCCAAAAGAAA-AAGTAGCCTGTCCCCTTTTC
Gbem_R1010	-	352898	352950	AAAAAAGGGGACAGGCTACTTTATTGTTTTAAA-AAGTAGCCTGTCCCCTTTTC
Gbem_R1011	+	372066	372117	ACCAAAGGGGACAGGCTACTTTTTTGT-AAAAA-AAGTAGCCTGTCCCCCTTAA
Gbem_R1012	+	475902	475954	AAAAAGGGGGACAGGCTACTTTTTGAAATGAAA-AAGTAGCCTGTCCCCCTTTT
Gbem_R1013	-	504268	504320	TTAAAGGGGGACAGGCTACTTTTTGAAATCAAA-AAGTAGCCTGTCCCCTTTTT
Gbem_R1014	+	557018	557070	AGAAAAGGGGACAGGCTACTTTTAAAAAACAAA-AAGTAGCCTGTCCCCCTTTT
Gbem_R1015	+	731066	731118	GAAAAAGGGGACAGGCTACTTTTTGAAAACAAA-AAGTAGCCTGTCCCCCTTTT
Gbem_R1016	+	847536	847589	GAAAAAGGGGACAGGCTACTTTTTTCAAAAGAAAAAGTAGCCTGTCCCCCTTCG
Gbem_R1017	-	1313004	1313056	ATAAAAGGGGACAGGCTGGTTTTTCAGATCAAA-AAGTAGCCTGTCCCCCTTAA
Gbem_R1018	-	1313098	1313150	ATAAAAGGGGACAGGCTACTTTTTCAAATCGAA-AACTAGCCTGTCCCCTTTTC
Gbem_R1019	+	1569152	1569204	TGAAAGGGGGACAGGCTACTTTTTCAAATCAAA-AAGTAGCCTGTCCCCTTTAG
Gbem_R1020	-	1569333	1569385	AAAAAGGGGGACAGGCTACTTTTTGATTTGAAA-AAGTAGCCTGTCCCCCTTTT
Gbem_R1021	+	1656256	1656308	GAAAAAGGGGACAGGCTACTTTTTGAAATCAAA-AAGTAGCCTGTCCCCCTTAA
Gbem_R1022	+	1656412	1656464	GCAAAAGGGGACAGGCTACTTTTCAAAATAAAA-AAGTAGCCTGTCCCCCTTTC
Gbem_R1023	+	1962051	1962103	TTTAAAGGGGACAGGCTACTTTTTGATTTGAAA-AAGTAGCCTGTCCCCTTTTT
Gbem_R1024	+	2246735	2246787	GGCAAGGGGGACAGGCTACTTTTTGAGATCAAA-AAGTAGCCTGTCCCCTTTTT
Gbem_R1025	+	2395282	2395334	AAAAAGGGGGACAGGCTACTTTTTGAAATCAAA-AAGTAGCCTGTCCCCTTTTC
Gbem_R1026	+	2395503	2395555	AAAAAGGGGGACAGGCTACTTTTTGAAATCAAA-AAGTAGCCTGTCCCCTTTTC
Gbem_R1027	+	2410940	2410993	ACAAAAGGGGACAGGCTACTTTTTCGAAAAATAAAAGTAGCCTGTCCCCTTTAC
Gbem_R1028	-	2676656	2676708	AAAAAGGGGGACAGGCTACTTTTTCAAATCAAA-AAGTAGCCTGTCCCCTTTTC
Gbem_R1029	-	2796473	2796525	TGAAAAGGGGACAGGCTACTTTTTGAAAAAATA-AAGTAGCCTGTCCCCTTTTC
Gbem_R1030	-	2796650	2796702	AAAAAGGGGGACAGGCTACTTTTTCGAAAGATA-AAGTAGCCTGTCCCCCTTTG
Gbem_R1031	-	3121004	3121056	TAAAAGGGGGACAGGCTACTTTTTTCCCGAAAA-AAGTAGCCTGTCCCCCTTAG
Gbem_R1032	-	3521628	3521680	AAAAAAGGGGACAGGCTACTTTTCCAAAACAAA-AAGTAGCCTGTCCCCGTAGC
Gbem_R1033	-	3576088	3576140	GAAAAAGGGGACAGGCTACTTTTTGAAATGAAA-AAGTAGCCTGTCCCCCTTTA
Gbem_R1034	+	3650478	3650530	GAAAAAGGGGACAGGCTACTTTTCGATTCGAAA-AAGTAGCCTGTCCCCTTTTA
Gbem_R1035	-	3650625	3650677	ACAAAAGGGGACAGGCTACTTTTTATAATCAAA-AAGTAGCCTGTCCCCCTTTT
Gbem_R1036	-	3689530	3689581	ATGGAAGGGGACAGGCTACTTTTC-TAATAAAA-AAGTAGCCTGTCCCCTTTTT
Gbem_R1037	-	3692491	3692543	AAAAAGGGGGACAGGCTACTTTTTGATTTGAAA-AAGTAGCCTGTCCCCTTTTT
Gbem_R1038	-	3717151	3717203	CGAAAAGGGGACAGGCTACTTTTTCATTTAAAA-AAGTAGCCTGTCCCCCTTTT
Gbem_R1039	+	3785494	3785546	AGAAAAGGGGACAGGCTACTTTTTTATTTGTAA-AAGTAGCCTGTCCCCCTTTC
Gbem_R1040	-	3785632	3785684	GAAAAAGGGGACAGGCTACTTTTTCAAAACGAA-AAGTAGCCTGTCCCCTTTTA
Gbem_R1041	+	3821772	3821824	AAACTAGGGGACAGGCTACTTTTTCGTTCGAAA-AAGTAGCCTGTCCCCTTTTG
Gbem_R1042	+	3832565	3832617	AAAAAGGGGGACAGGCTACTTTTTCAAACGAAA-AAGTAGCCTGTCCCCTTTAC
Gbem_R1043	-	3856540	3856592	GGAAAAGGGGACAGGCTACTTTTTGAAATCAAA-AAGTAGCCTGTCCCCCTTTT
Gbem_R1044	+	4175431	4175483	TAAAAAGGGGACAGGCTACTTTTTGAGATCAAA-AAGTAGCCTGTCCCCCTTTT
Gbem_R1045	-	4182799	4182851	GAAAAAGGGGACAGGCTACTTTTTGAAATCAAA-AAGTAGCCTGTCCCCCTTTT
Consensus				RRAAARGGGGACAGGCTACTTTTTSAWWTSAAA AAGTAGCCTGTCCCCYTTTY

Gbem_R1101	-	83298	83330	AGGGGACTGGCTCCG-CAGGTGCCTGTCCCCCTT
Gbem_R1102	-	310679	310711	AGGGGACTGGCTCCG-AAGGTGCCTGTCCCCCTG
Gbem_R1103	+	333798	333831	AGGGGACTGGCTCCGCCAGGTGCCTGTCCCCCTT
Gbem_R1104	-	371260	371293	AGGGGACTGGCTCCGCAAGGTGCCTGTCCCCCTT
Gbem_R1105	-	476866	476899	GGGGGACTGGCTCCGCAAGGTGCCTGTCCCCCTT
Gbem_R1106	-	752387	752420	AGGGGACTGGCTCCGTCAGGTGCCTGTCCCCCTT
Gbem_R1107	+	852995	853028	AGGGGACTGGCTCCGCCAGGTGCCTGTCCCCCTT
Gbem_R1108	+	902680	902713	TGGGGACTGGCTCCGCCAGGTGCCTGTCCCCCTT
Gbem_R1109	+	928060	928093	AGGGGACTGGCTCCGCAAGGTGCCTGTCCCCCTT
Gbem_R1110	+	928106	928139	AGGGGACTGGCTCCGTAAGGTGCCTGTCCCCCTT
Gbem_R1111	-	1070972	1071005	AGGGGACTGGCTCCGCCAGGTGCCTGTCCCCCTG
Gbem_R1112	+	1168595	1168628	AGGGGACTGGCTCCGCAAGGTGCCGGTCCCCCTT
Gbem_R1113	-	1185259	1185291	CGGGGACTGGCTCCG-CAGGTGCCTGTCCCCCTT
Gbem_R1114	+	1387272	1387305	GGGGGACTGGCTCCGCAAGGTGCCTGTCCCCCCG
Gbem_R1115	-	1419689	1419722	TGGGGACTGGCTCCGCCAGGTGCCTGTCCCCCTT
Gbem_R1116	+	1461988	1462021	AGGGGACTGGCTCCGCAAGGTGCCCGTCCCCCTG
Gbem_R1117	-	1490455	1490488	AGGGGACTGGCTCCGCCAGGTGCCTGTCCCCCTT
Gbem_R1118	-	1638716	1638749	GGTGGACTGGCTCCGCCAGGTGCCTGTCCCCCTC
Gbem_R1119	+	1646492	1646524	GGGGGACTGGCTCCG-CAGGTGCCTGTCCCCCTG
Gbem_R1120	-	1660470	1660502	GGGGGACTGGCTCCG-CAAGTGCCTGTCCCCCTT
Gbem_R1121	+	1674985	1675017	AGGGGACTGGCTCCG-AAGGTGCCTGTCCCCCTC
Gbem_R1122	+	1687568	1687600	GGGGGACTGGCTCCG-CAGGTGCCTGTCCCCCTT
Gbem_R1123	+	1697182	1697214	GGGGGACTGGCTCCG-AAGGTGCCTGTCCCCCTT
Gbem_R1124	-	1818793	1818826	AGGGGACTGGCTCCGCCAGGTGCCTGTCCCCTTA
Gbem_R1125	+	1906259	1906292	GGGGGACTGGCTCCGATAGGTGCCTGTCCCCCTT
Gbem_R1126	+	1917565	1917598	AGGGGACTGGCTCCGCAAGGTGCCTGTCCCCCTT
Gbem_R1127	+	1977828	1977861	AGGGGACTGGCTCCGCAAGGTGCCTGTCCCCCTT
Gbem_R1128	+	1978627	1978659	GGGGGACTGGCTCCG-CAGGTGCCTGTCCCCTTG
Gbem_R1129	-	2001407	2001440	AGGGGACTGGCTCCGCAAGGTGCCTGTCCCCCTG
Gbem_R1130	+	2263566	2263599	AGGGGACTGGCTCCGCAAGGTGCCTGTCCCCGTT
Gbem_R1131	+	2344762	2344795	AGGGGACTGGCTCCGCAAGGTGCCTGTCCCCCTT
Gbem_R1132	+	2420110	2420143	GGGGGACTGGCTCCGCAAGGTGCCTGTCCCCCCT
Gbem_R1133	+	2427416	2427449	AGGGGACTGGCTCCGCAAGGTGCCTGTCCCCCTG
Gbem_R1134	+	2438684	2438717	AGGGGACTGGCTCCGCCAGGTGCCTGTCCCCCTT
Gbem_R1135	+	2438996	2439029	AGGGGACTGGCTCCGTCAGGTGCCTGTCCCCCTT
Gbem_R1136	+	2439320	2439353	AGGGGACTGGCTCCGTCAGGTGCCTGTCCCCCTT
Gbem_R1137	+	2439659	2439692	AGGGGACTGGCTCCGTCAGGTGCCTGTCCCCCTG
Gbem_R1138	+	2484351	2484383	GGGGGACTGGCTCCG-CAGGTGCCTGTCCCCCTT
Gbem_R1139	+	2530499	2530531	GGGGGACTGGCTCCG-CAGGTGCCTGTCCCCCGG
Gbem_R1140	-	2530584	2530617	GGGGGACTGGCTCCGCAAGGTGCCTGTCCCCCTT
Gbem_R1141	+	2530629	2530661	AGGGGACTGGCTCCG-CAGGTGCCTGTCCCCTTT
Gbem_R1142	+	2589037	2589070	CGGGGACTGGCTCCGCAAGGTGCCTGTCCCCCTT
Gbem_R1143	-	2589518	2589551	AGGGGGCTGGCTCCGCAAGGTGCCTGTCCCCCTT
Gbem_R1144	-	2599315	2599348	AGGGGACTGGCTCCGTCAGGTGCCTGTCCCCTTT
Gbem_R1145	-	2681888	2681921	AGGGGACTGGCTCCGCCAGATGCCTGTCCCCTTG
Gbem_R1146	-	2702310	2702343	AGGGGACTGGCTCCGCCAGGTGCCTGTCTCCCTT
Gbem_R1147	+	2748750	2748782	AGGGGACTGGCTCCG-CAGGTGCCTGTCCCCCTT
Gbem_R1148	-	3124195	3124228	AGGGGACTGGCTCCGCCAGGTGCCTGTCCCCCTT
Gbem_R1149	-	3124233	3124266	AGGGGACTGGCTCCGCCAGGTGCCCTCCCCCTGC
Gbem_R1150	+	3262856	3262889	AGGGGACTGGCTCCGCCAGGTGCCTGTCCCCTTT
Gbem_R1151	+	3268847	3268880	AGGGGACTGGCTCCGCCAGGTGCCTGTCCCCCTT
Gbem_R1152	-	3324831	3324864	AGGGGACTGGCTCCGCAAGGTGCCTGTCCCCTTA
Gbem_R1153	+	3376613	3376646	GGGGGACTGGCTCCGCAAGGTGCCTGTCCCCTTT
Gbem_R1154	+	3376665	3376697	AGGGGACTGGCTCCG-CAGGTGCCTGTCCCCTTT
Gbem_R1155	+	3376716	3376748	AGGGGACTGGCTCCG-CAGGTGCCTGTCCCCTTT
Gbem_R1156	+	3407412	3407444	GGGGGACTGGCTCCG-CAGGTGCCTGTCCCCCTG
Gbem_R1157	-	3421513	3421546	AGGGGACTGGCTCCGCCAGGTGCCTGTCCCCCTT
Gbem_R1158	+	3424111	3424144	GGGGGACTGGCTCCGCAAGGTGCCTGTCCCCCTT
Gbem_R1159	+	3424154	3424187	GGGGGACTGGCTCCGCAAGGTGCCTGTCCCCCTT
Gbem_R1160	+	3717273	3717306	AGGGGACTGGCTCCGCCAGGTGCCTATCCCCCTT
Gbem_R1161	+	3717318	3717351	AGGGGACTGGCTCCGCCAGGTGCCTGTCCCCCTT
Gbem_R1162	+	3760069	3760102	AGGGGACTGGCTCCGCAAGGTGCCTGTCCCCCTT
Gbem_R1163	+	3842765	3842798	AGGGGACTGGCTCCGCCAGGTGCCTGTCCCCTTC
Gbem_R1164	-	4060287	4060320	AGGGGACTGGCTCCGTCAGGTGCCTGTCCCCCTG
Gbem_R1165	-	4127064	4127097	AGGGGACTGGCTCCGCCAGGTGCCTGTCCCCCTT
Gbem_R1166	-	4129600	4129633	GGGGGACTGGCTCCGCAAGGTGCCTGTCCCCCTG
Gbem_R1167	-	4201156	4201188	AGGGGACTGGCTCCG-CAGGTGCCTGTCCCCCTT
Gbem_R1168	+	4336042	4336075	AGGGGACTGGCTCCGTCAGGTGCCTGTCCCCCTT
Gbem_R1169	-	4419617	4419650	AGGGGACTGGCTCCGCAAGGTGCCTGTCCCCCTT
Gbem_R1170	-	4530233	4530265	GGGGGACTGGCTCCG-CAGGTGCCTGTCCCCCTT
Gbem_R1171	-	4549683	4549716	GGGGGACTGGCTCCGCCAGGTGCCTGTCCCCCTT
Gbem_R1172	-	4605454	4605487	GGGGGACTGGCTCTGCAAGGTGCCTGTCCCCCTG
Consensus				RGGGGACTGGCTCCGCMAGGTGCCTGTCCCCCTT
