# Supplementary material for: The genome of Geobacter bemidjiensis, exemplar for the subsurface clade of Geobacter species that predominate in Fe(III)-reducing subsurface environments
Source: BMC Genomics. 2010 Sep 9;11:490. doi: 10.1186/1471-2164-11-490 (PMC2996986; doi:10.1186/1471-2164-11-490)
Supplement: Additional file 11 — Figure S5. Multicopy nucleotide sequences of the G. bemidjiensis genome: base coordinates and alignments. (See also Table S6.). [file 1471-2164-11-490-S11.PDF]

```
Gbem_R5001  +  1681969  1682006  AAGTCC--CCCTTTGCGAAGGGGGATTTAGGGGGATTTGC
Gbem_R5002  -  1697232  1697269  AAGTCC--CCCTTTGCGAAGGGGGATTTAGGGGGAGTTGC
Gbem_R5003  +  1697304  1697343  ACGTCCCCCCCTTTGCGAAGGGGGGACAGGGGGGATTTGG
Gbem_R5004  +  2394352  2394389  AAGTCC--CCCTTTGCGAAGGGGGATTTAGGGGGATTTTC
Gbem_R5005  +  4064641  4064678  GAGTCC--CCCTTTGCGAAGGGGGATTTATGGGGATTTAC
Gbem_R5006  +  4605374  4605410  AAATCC-CCCTTTTACAAAGGGGGACTTAG--GGATTTTC

Consensus                        AAGTCC  CCCTTTGCGAAGGGGGATTTAGGGGGATTTGC

Gbem_R5007  +  186796  186837  GCTTGGGAACCCAATGCGTCGCAAAGCTCCAGCTTTGCATTC
Gbem_R5009  +  1914298  1914338  GCTTGGGAACCCA-TGCGGCGCAAACCTCCAGCTTTGCCTCC
Gbem_R5011  +  2118206  2118246  TCTTGGCTTTAGTCTGGTTCCCAA-GCTCCAGCTTGGGAACC
Gbem_R5013  -  2583697  2583738  GCTTGGGAACCCAATGCGGAGAAAAGCTCCAGCTTTGATCTA
Gbem_R5015  +  3421355  3421397  GCTTGGGAACCCAATGCGGCGCAAAGCTCCAGCTTTGCATCC

Consensus                        GCTTGGGAACCCAMTGCGKCGCAAAGCTCCAGCTTTGCATYC

Gbem_R5008  +  186842  186897  GAAGCTGGAGCTTCGGGATGGCCTTGCGTTCCCAAGGTGGACCTTGGGAACGAGAG
Gbem_R5010  +  1914343  1914398  GAAGCTGGAGCTTCGGCAGG-CCTTGCGTTCCCAAGGTGGACCTCGGGAACGAGAT
Gbem_R5012  +  2118281  2118335  GAAGCTGGAGCTTCGCAGGGTCCTTGCGTTCCCAAGGTGGACCTTGGGAACGAGAT
Gbem_R5014  -  2583638  2583693  GAACCTTGAGCTTCGGCAGG-CCTTGCGTTCCCAAGGTGGACCTTGGGAACGAGAT
Gbem_R5016  +  3421402  3421457  GAAGCTGGAGCTTCAGGCAGGCCTTGCGTTCCCAAGGTGGACCTTGGGAACGAGAT
Gbem_R5017  -  333742  333796  GAAGCTGGAGCTTCGGCAGG-CCTTGCGTTCCCAAGGTGGACCTTGGGAACGAGAT
Gbem_R5018  -  376690  376744  GAAGCTGGAGCTTCGGTCTG-CATTGCGTTCCCAAGGTGGACCTTGGGAACGAGAT

Consensus                        GAAGCTGGAGCTTCGGNAGG CCTTGCGTTCCCAAGGTGGACCTTGGGAACGAGAT

Gbem_R5019  +  498572  498697  GGGTGACATAATACCGATAATTCTGTAACACATGAGGAGCAGAAGAAAGATACA------------CCCACAT--GGGTGGAGATGGACTTCGCGTGCCTATA---AAAGTGGGGGA-GAAGTCTGAGAAAAACAAAGCAAGTC
Gbem_R5020  +  2317968  2318103  AGGGGAGACAATACCGAGAATTCTA-AACACATGAGGTGCAGGAGAAAGATCCCACGCGGGTCGAGACAAACTTAAAAGTGGGCAGAAGTCTAAGTGATCAGCGTCT---TAGTCCCT-TTAT-GGAG-GCCTGGGGGCAGGTC
Gbem_R5021  +  3318819  3318954  CCGGGACACAATACCTATAATTTTGCAACACATGAGGGACAGAAGATTGGTCCCCCGACTTCCGTGCCACACCACTCATTTCTGCGGTTTCGCCGGCCTACC---AGGATGAC--CT-TTA-CTGAGGTCCCGTCCCGTAATC
Gbem_R5022  -  3434753  3434892  CGGGAACAAAATACCGATAATTCTGCAACACATGAGGCACAGAAGAA---GTCAGTTCTCTTCTTCACAGATTGTTTTCCAGGCAATGCAAAAGCCGCTACCGACTAAGTAGCGGCTTTTGTCATGTTGAGCGAAAGGGGGCA
Gbem_R5023  -  3435586  3435725  CGGGGACACAATACCAATAATTCTGCAACACATGAGGCGCAGAAGAA---TTCAGTTCTCTTTTTCACAGATTGTTTTCCAGGCAATGCTAAAGCCGCTACCGACTAAGTAGCGGCTTTTGTCATGGTGAGCGAAAGGGGGCA
Gbem_R5024  +  4512286  4512415  GCGGCGGTTACGAAGAGCCGGTATTCAACCC----GGACGACGAGA----TCCCGTTC-------TAGACAT--ATTAAGGAAAGAA-TAAAAGCCGCTACCGCCAAGGTCGCGGCTTTTGTCGTTTTGAGGCGGGTTGGGAT
Gbem_R5025  +  4514431  4514552  CGGGGACATAATACCTATAATTTTGCAACACATGAGACGCAGAAGAAGAATTCAG-TCTCTTCTTCACAGATTGTTATCCAGGCTATGCAAAAGCCGCTACC-------------------TATTAA---GAGCAAAGGGGGTC

Consensus                        CGGGGACAYAATACCNATAATTCTGCAACACATGAGGNGCAGAAGAA   TYCAGTTCTCTYCTTCMCASATTRTTWTYSAGRMRAWGTWAAAGCCGCTACCGNCWARGTRGCGGCTTTTRTCNKRGTGAGCGAAGGGGGGTC

Gbem_R5026  +  2214896  2215014  CGGGTGTGGCGGGGTCGGACCAAGGTAGGATGCTGTAATGACACGACAATACCCGAAAAACTCCCTGTATTTCACCCTTTGAAGCACCTTTTTACCGGCAAAATGGGGCCAATAAGGCA
Gbem_R5027  +  2215087  2215111  CGGGTGTGGCGGGGTCGGACCAAGG
Gbem_R5028  +  2215115  2215233  CGGGTGTGGCGGGGTCGGACCAAGGTAGGATGCTGTAATGACACGACAATACCGGAAAAACTCCCTGTATTTCACCCTTAGAAGCACCTTTTTACCGGCAAAATGGGGCCAATAAGGCA
Gbem_R5029  -  3436374  3436492  AGGTCCGGGCGGGGTCGGACCAAGGCAACATGTTGTAATGACACGATAAAGACAAACAAAGTCCATATATTTTACCCTTAAAAGCATGTTTTCACCGGCAAAAAAGGGCCAATAAAGCA
Gbem_R5030  -  3436547  3436665  CGCGTGTTGCGGGGTCGGACCAAGGCAACGTGTTGTAATGACACGATAAAGACAGACAAAGTCCATGTATTTTACCCTTAAAAGCATGTTTTCACCGGCAAAAAGGGGCCAATAAAGCA

Consensus                        CGGGTGTGGCGGGGTCGGACCAAGGYARSATGYTGTAATGACACGAYAAWRMCAGAMAAAGTCCATGTATTTTACCCTTAAAAGCATGTTTTCACCGGCAAAAAGGGGCCAATAAAGCA

Gbem_R5031  -  498155  498426  CCATTCAGCACATGACCTTTGACATAGATGAAGGATCTCTATATATTGCGATCATTTAAGCTGGGTCA-ATGAAGGTTGCACGTGAGTAGCGTCAGCATATTCCACTCGAG------AAGGGGGATGAACAGTTTTCTG
Gbem_R5032  +  1439611  1439888  ACAATCCGCACTTCACCTTTGACAGTGATGGTAGATCTCTATATATTGCGGTTGTTTTAGCTGGAACAAATGAAATTTGCATATGAGTAGCGTATGCATGTTCCATTCAGATGGGA-AGGGGGGATGGACAGTTTTCTG
Gbem_R5033  +  2208853  2209131  CCATTCATCACTTGACCTTTGACATGGATGATAGATGTCTATATATTGCGATTGTTTTAAGTTGCGTCAAATGAAATATACATATTGATATCGTTTGCAGAGTCGATTCAGGGGGAGGAGGGGGGGATGGACGGTTTTCTG

                                 TCCATCCCCCCAATTGGCACAAATTCCGACCATCACAGCGCGTCATATCAATGAGTTGGGCGAACGTAGCCTGATAGATATTTTAATGTTGCATGCCGACAAGGGGGGATGACGGCAATTTCCTCCTAATTACATAGTTA
                                 TCCATCCCCCCAATTGGCACAAAATCCGGTCGTTACAGTGTGTGATATCAGCGGGTTACGCCAATATGATCGACAAATCTTTTTAATGTTGTACGGCGACGAGGGGGGATGACGGCAATTTCCCTTTAATTACATAGTTA
                                 TCCATCCCCCCAATTGGCGCAAAATCCGGTCGCTACAGTCTGCCATATCAGTGGGTTACGTCAATTGGTTTGGTAAAAAAAATTTAATGTTGAGCGGCGACAAGGGGGGATGACGGCAACTTCCCTCTAATTACATAGTTG

Consensus                        CCATTCAGCACTTGACCTTTGACATNGATGATAGATCTCTATATATTGCGATTGTTTTAAGCTGGGTCAAATGAAATTTGCATATGAGTAGCGTNGCATATTCCATTCAGGGGGGG AGGGGGGATGGACAGTTTTCTG
                                 TCCATCCCCCCAATTGGCACAAAATCCGGTCGTTACAGTGTGTCATATCAGTGGGTTACGCCAATNTGNTCGGNAAAANATTTTAATGTTGNACGGCGACAAGGGGGGATGACGGCAATTTCCCTCTAATTACATAGTTA

Gbem_R5034  -  4008420  4008660  TTTCAACAGACTGTTTCTTTGGTGCGACCAGTGATAATGTATGACTCAGCTAGCTAGCTAAGAGAAATCTCGGAATAAGGAGGTACCCGACCATAGAGGAAACCCCACCGATTAGGATTCTCAAAACTTGAAAGTAGTAACGGTCCGGTCCGCATAGGTACCGGCATCCACCCTTGAAAAGGGTACCCAATGTAAGACACTACAACCAGAGGCCCGCCGGATTCGGCGGGCCTTTCAATTT
Gbem_R5035  -  4008968  4009192  TTACAACAGACTGTTTCTTTCGCGTCAGTGGTGTTAATTTGAAA-TCAGCTAGCTAGTCA-GAG-TATCTCGAGCCAAGGAGGTAAACCGTTTTAGAGGAAACTCCACCGATCAGGATTCTCAAAAAATTGACGGCTGTAATAGTCCGGAGCTCACAGGCTCCGGCATCCACCCTTGCAAAAGGGTTCTCGATG--AGA-----------GAGGCCCGCCGAAACCGGCGGGCCTTTCAATTT

Consensus                        TTWCAACAGACTGTTTCTTTSGYGYSASYRGTGWTAATKTRWRA[C]TCAGCTAGCTAGYYAAGAGAWATCTCGRRMYAAGGAGGTAMACSRYYWTAGAGGAAACYCCACCGATYAGGATTCTCAAAAMTTGAMRGYWGTAAYRGTCCGGWSCKCAYAGGYWCCGGCATCCACCCTTGMAAAGGGTWCYCRATG[N]0-2AGA [N]0-11   GAGGCCCGCCGRAWYCGGCGGGCCTTTCAATTT

Gbem_R5036  +  2236867  2237049  CCTTGACAAGAACAATGAGAAAAATTATATTTCATCAAGTTAAAGGGGAGTAGTTATCGGCCGGAGAAATGGCCGACCCGGTTTCCGTCAATACGGTCTTAGGA-CCCGGAGCCGGGGC-A--GATA--TCCTGT--CA-ACAAGACCTTTATCCAGGCATTAACGT--GTCGCGGGTAAAGGTTTTTTTTATT
Gbem_R5037  +  3392036  3392220  GGTTGACATAGCGTCAGACAGTTAATATTTATGGCCACCTGTAAAGGGGAGTAGCTATCGGCCGGAGAAATGGCCGACCCCTGGTTCGTCAAGACGGTGGCAACA-CCCGGACCAGGGGATACGGATTCATCTAA---CA-GCAAGACCTTTACCCAAGCGAAGACATTCGCCCGTGGGTAAAGGTCTTTTTTTTT
Gbem_R5038  +  3773253  3773441  GGTTGACACATCGCCAGTCATTACCTATATTTCACAAAGTATAAAGGGGAGTAGCAATCGGTTACCACGGCAACCGACCTGTGTTCGTCAAGACGGCTGACAAAGCCCGGACACGGGGCGA-TGACACCTGTCAGAGCATGCAAGACCTTTATCCA-GTGCCGACGC--GCC-TGGATAAAGGTCTTTTTATT

Consensus                        GGTTGACANANCGNCAGACANTAANTATATTTCACAAAGTTAAAGGGGAGTAGNTATCGGCCGGAGAAATGGCCGACCCNGTGTTCGTCAAGACGGTNGNAANA CCCGGACCCGGGGCKA KGATACCTCTNAK  CA GCAAGACCTTTATCCARGCGNNGACGT  GCCGTGGGTAAAGGTCTTTTTATT

Gbem_R5039  +  1716691  1716871  TCTCGCTATAGTGGCGAGAGGTTGAGGAGTATCGGCGCTCACTTCGGGTTTGAAGATTCTGCAGTGGTGAAGGCAAGCACGAGACTAGAAACAACAGCGAAACAACCGTCCACGAAAAGGTCAAAGAGATTCTGCAATCCCCTCGGTTGTCCAGGTCCAGACCAGACCCCTCAGCCCCCCT
Gbem_R5040  +  1716879  1717059  TCTCGCTATAGTGGCGAGAGGTTGAGGAGTATCGGCGCTCACTTCGGGTTTGGAGATTCTGCAGTGGTGAAGGCAAGCACGAGACTAGAAACAACAGCGAAACAACCGTCCACGAAAAGGTCAAAGAGGTTCTGCAATCCTCTCGGTTGTCCAGGTCCTGACCTGACCCCTCAGCCCCTCT

Consensus                        TCTCGCTATAGTGGCGAGAGGTTGAGGAGTATCGGCGCTCACTTCGGGTTTGRAGATTCTGCAGTGGTGAAGGCAAGCACGAGACTAGAAACAACAGCGAAACAACCGTCCACGAAAAGGTCAAAGAGRTTCTGCAATCCYCTCGGTTGTCCAGGTCCWGACCWGACCCCTCAGCCCCYCT

Gbem_R5041  -  511598  511760  GTTCCTCGTTACGATGAGGCGTTTGCTCAAACACAGGCTACTGCCCGGAAACGTCGAAAGACGCCAACGGGTAGACCAGGTCTTGCCGGCTTAAGGCTTGACCCAAGGTAGCTGGTCCCCCGGGACCTACGTTGCGCACTGCCGAAAACCTACGAGAGGGGAA
Gbem_R5042  -  3732445  3732607  GTTCCTCGTTAGCATGAGGCGTTTGCACAAACAGAGGCTACTGCCCAAAAACGTCGAAAGACGCCAATGGGTAGACCAGGGCTTGCCGGATTAAGGCTTGACCTAAGGTAGCTGGTTCTTCCTGACCTACGTTGTGCACTGCTGAAAACCTACGAGTGGGGAA

Consensus                        GTTCCTCGTTASSATGAGGCGTTTGCWCAAACASAGGCTACTGCCCRRAAACGTCGAAAGACGCCAAYGGGTAGACCAGGKCTTGCCGGMTTAAGGCTTGACCYAAGGTAGCTGGTYCYYCSKGACCTACGTTGYGCACTGCYGAAAACCTACGAGWGGGGAA

Gbem_R5045  -  493928  493996  AAAAAGCAAATGTACCAATAAGGCCCATGGACGCCGACCGCCCGATGAAACTGGTCGGGCGGGTCATGG
Gbem_R5046  +  2212711  2212779  AAAAAGCAAATGTTCCAACAAGCCCCATGGACGCCGACCGCCCGAAAAAACTAGGGCGGGCGGGTCATGG

Consensus                        AAAAAGCAAATGTWCCAAYAAGCCCCATGGACGCCGACCGCCCGAWRAAMYWGGKCGGGCGGGTCATGG

Gbem_R5047  +  2494723  2494781  AAAGCAGTTCCACTTTCCCGGAAACCGGTCCAGCTTTTTTTCTAATCTGGTACAGCTTTT
Gbem_R5048  +  2494807  2494865  AAAACGGTTCCACTTTTCCCAAAACCCGTTCAGCTTTTTTTTCTAATCTGGTACAGTTTTG
Gbem_R5049  +  2494869  2494927  AAAACCGGTCCACTTTTTCCAGAACCGGTTCAGCTTTTTTTTCTAATCTGGTACAGTTTTG

Consensus                        AAAACNGTTCCACTTTTCCCAAAACCGGTTCAGCTTTTTTTTCTAATCTGGTACAGTTTTG

Gbem_R5050  +  4035713  4035770  CCTCCACCTGTAAGTCTTCTGTAAGCCGCTCTCCATTACCATCCCCGCAATAAGGGGC
Gbem_R5051  +  4037372  4037429  CCTCCAGCTGTAAGCCTTCTGTAAGCCCCCCTCCATTATCATCCCCGCAATAAGGGGC

Consensus                        CCTCCASCTGTAAGYCTTCTGTAAGCCSCYCTCCATTAYCATCCCCGCAATAAGGGGC

Gbem_R5052  -  3434413  3434462  TTCAGTTCCCTTATCCTTTACTCTTGACGACGCCGACTGGTTTAGGCTAT
Gbem_R5053  -  3436086  3436135  TTCAGTTCCATTATCCTTTACTCTTGACGACGCCGACTGGTTTAGGCTAT

Consensus                        TTCAGTTCCMTTATCCTTTACTCTTGACGACGCCGACTGGTTTAGGCTAT

Gbem_R5054  -  3326609  3326646  AACAAAGGCACTTGGCTGATTAGCTAAGTGCCTTTGTT
Gbem_R5055  -  3331519  3331556  AACAAAGGCACTTGGCTGATTAGCTAAGTGCCTTTTTG

Consensus                        AACAAAGGCACTTGGCTGATTAGCTAAGTGCCTTTTTG

Gbem_R5056  -  1721232  1721269  AAAATTAGCTTGATCGCAAGATCTCTTTAATTTAAATT
Gbem_R5057  -  1726823  1726860  AAAATTAACTTGATCGGAAGATCTCTTTAATTTAAATT

Consensus                        AAAATTARCTTGATCGSAAGATCTCTTTAATTTAAATT

Gbem_R5058  +  1436500  1436536  GTAGCTCCTTTAGCGCCAGGTCCTACGACCTGGCGCC
Gbem_R5059  +  1439545  1439581  GTAGCTCCTTTTGCGCCAGGTCCCACGACCTGGCGCC

Consensus                        GTAGCTCCTTTWGCGCCAGGTCCYACGACCTGGCGCC

Gbem_R5060  -  2669414  2669446  GCTCGCTTCAAAGGGGCGGCATCTTGACGGCAC
Gbem_R5061  -  2669493  2669525  GCTCACTTCAAGGGGGCGGCATCTTGACGGCAC

Consensus                        GCTCRCTTCAARGGGGCGGCATCTTGACGGCAC

Gbem_R5062  +  608793  608821  GGAGGAGGTGGTGGTGAAATGGTGGAGGA
Gbem_R5063  +  608839  608867  GGAGGAGGTGGTGGTGAAATGGTGGAGGA

Consensus                        GGAGGAGGTGGTGGTGAAATGGTGGAGGA

Gbem_R5064  +  392950  392978  GACAAGGCCCGGTGTCTGTACGAATGGGT
Gbem_R5065  +  394872  394900  GACAAGGCCCGGTGTCTGTACGAATGGGT

Consensus                        GACAAGGCCCGGTGTCTGTACGAATGGGT

Gbem_R5066  +  3564313  3564340  ACTGGCTCCGCAGGTGCCTGTCCCCCTT
Gbem_R5067  +  3564359  3564386  ACTGGCTCCGCAGGTGCCTGTCCCCCTT

Consensus                        ACTGGCTCCGCAGGTGCCTGTCCCCCTT

Gbem_R5068  +  2399832  2399858  AGATAAAGGTTAAGTAAAGGCAGTAGG
Gbem_R5069  +  2399877  2399903  AGATAAAGATTAAGTAAAGGCAGTAGG

Consensus                        AGATAAAGRTTAAGTAAAGGCAGTAGG

Gbem_R5070  +  500291  500314  TTTTTTTCAAATGTGTCATTTCCG
Gbem_R5071  +  500332  500355  TTTTTTCCAAATGTGTCATTTTCG

Consensus                        TTTTTTYCAAATGTGTCATTTYCG

Gbem_R5072  +  984142  984165  GGGGACGTTGAGCAACTGCTCTTT
Gbem_R5073  +  984178  984201  GGGGACGTTGAGCAACTGCTCTTT

Consensus                        GGGGACGTTGAGCAACTGCTCTTT

Gbem_R5074  -  3133695  3133977  TTAGCCACGGAGAAAATCTGAGAGAGTCAAAAACCAAAACCGCTCACAAGGAGAACTTCTGAGGACTTCTGAGAAAGGCAAAACCGGAGAGAGTCAAAAACTTTTTCTGGGAAAGGCAAAACCAAAACC
                                 TTAGCCACGGAGAAAATCTGAGAGAGTCAAAAACCAAAACCGCTCACAAGGAGAACTTCTGAGGAGTTCTGAGAAAGGCAAAACCGGAGAGAGTCAAAAACTTTCTCTGGGAAAGGCAAAGCCAAAACC
                                 TTAGCCACGGAGAAAATCTGAGA

Consensus                        TTAGCCACGGAGAAAATCTGAGAGAGTCAAAAACCAAAACCGCTCACAAGGAGAACTTCTGAGGASTTCTGAGAAAGGCAAAACCGGAGAGAGTCAAAAACTTTYTCTGGGAAAGGCAAARCCAAAACC
```
