# Supplementary material for: The genome of Geobacter bemidjiensis, exemplar for the subsurface clade of Geobacter species that predominate in Fe(III)-reducing subsurface environments
Source: BMC Genomics. 2010 Sep 9;11:490. doi: 10.1186/1471-2164-11-490 (PMC2996986; doi:10.1186/1471-2164-11-490)
Supplement: Additional file 13 — Figure S7. Multicopy nucleotide sequences of the G. bemidjiensis genome: base coordinates and alignments. (See also Table S6.). [file 1471-2164-11-490-S13.PDF]

Gbem_R7001	-	17505	17535	CAATTGACAATTGACGATTGACAATTGACAA
Gbem_R7002	+	30086	30116	CAATTGACGATTGACAATTGACAATTGACAA
Gbem_R7003	+	34013	34043	CAATTGACAATTGACAATTGACAATGGACAA
Gbem_R7004	+	100418	100448	CAATTGACAATTGACGATTGACAATTGACAA
Gbem_R7005	+	100497	100527	CAATTGACAATTGACAATTGACGATTGACAA
Gbem_R7006	+	100574	100604	CAATTGACAATTGACGATTGACAATTGACAA
Gbem_R7007	-	100720	100750	CAATTGACGATTGACAATTGACAATTGACAA
Gbem_R7008	-	100898	100921	CAATTGACAATTGACAATTGACAA
Gbem_R7009	-	100938	100954	CAATTGACAATTGACAA
Gbem_R7010	+	182120	182150	CAATTGACAATTGACGATTGACGATTGACAA
Gbem_R7011	-	280129	280159	CAATTGACAATGGACAATTGACAATTGACAA
Gbem_R7012	+	305308	305338	CAATTGACAATTGACGATTGACAATTGACAA
Gbem_R7013	-	305471	305501	CAATTGACAATTGACAATGGATAATTGACAA
Gbem_R7014	+	418021	418051	CAATTGACGATTGACAATTGACAATTGACAA
Gbem_R7015	-	418426	418456	CAATTGATAATTGACAATTGATAATTGACGA
Gbem_R7016	-	418491	418514	CAATTGACAATTGACAATTGACAA
Gbem_R7017	+	681382	681412	CAATTGACAATTGACAATGGACAATTGACAA
Gbem_R7018	-	681529	681559	CAATTGACAATTGACGATTGACAATTGACAA
Gbem_R7019	-	1035386	1035416	CAATTAACAATTGACGATTGACAATTGACAA
Gbem_R7020	+	1040204	1040234	CAATTGACAATTGACAATGGACAATTGACAA
Gbem_R7021	-	1040346	1040376	CAATTGACAATTGACAATTGATAATTGACAA
Gbem_R7022	+	1072185	1072215	CAATTGACAATGGATAATTGACAATTGACGA
Gbem_R7023	+	1376323	1376346	CAATTGACAATGGACAATGGACGA
Gbem_R7024	-	1376450	1376480	CAATTGACAATTGATAATTGACAATTGACGA
Gbem_R7025	-	1382195	1382232	CAATTGACGATAGACAATGGACAATGGACAATTGACAA
Gbem_R7026	-	1684279	1684309	CAATTGACAATTGATAATTGACAATTGACGA
Gbem_R7027	+	1813452	1813482	CAATTGACAATTGACAATTGATAATTGACAA
Gbem_R7028	+	1843360	1843390	CAATTGACAATGGACAATTGACAATTGACAA
Gbem_R7029	+	2228184	2228242	CAATTGACAATTGACGATTGACAATTGACAATTGACAATTGACAATTGACAATTGACAA
Gbem_R7030	-	2366088	2366118	CAATTGACAATTGACGATTGATAATTGACAA
Gbem_R7031	+	2544943	2544973	CAATTGATAATTGACAATTGACAATTGACGA
Gbem_R7032	-	2545085	2545108	CAATTGACAATTGACAATTGACAA
Gbem_R7033	-	2794849	2794858	CAATTGACAA
Gbem_R7034	-	2794882	2794919	CAATTGACAATTGACGATTGACAATTGACAATTGACAA
Gbem_R7035	+	2848041	2848071	CAATTGACAATTGACGATTGACAATTGACGA
Gbem_R7036	+	3048974	3049004	CAATTGACAATTGACAATTGACAATTGACAA
Gbem_R7037	-	3060403	3060433	CAATTGACGATTGACAATTGACAATTGACAA
Gbem_R7038	-	3164584	3164614	CAATTGACAATTGATAATTGACAATTGACGA
Gbem_R7039	+	3174432	3174455	CAATTGACAATTGATAATTGACAA
Gbem_R7040	-	3174585	3174615	CAATTGACAATTGACGATTGACAATTGACAA
Gbem_R7041	-	3565525	3565555	CAATTGACAATTGACAATTGACAATTGACAA
Gbem_R7042	-	3811606	3811636	CAATTGGCAATGGACAATTGACAATTGACGG
Gbem_R7043	+	3813374	3813404	CAATTGACAATTGATAATTGACAATTGACAA
Gbem_R7044	-	3813541	3813571	CAATTGACAATTGACAATTGACAATTGACAA
Gbem_R7045	+	3814303	3814326	CAATTGACAATTGACAATTGACAA
Gbem_R7046	-	3814440	3814470	CAATTGACGATTGACAATTGACAATTGACAA
Gbem_R7047	-	3892585	3892615	CAATTGACAATTGACAATGGATAATTGACAA
Gbem_R7048	+	3969160	3969183	CAATTGACAATTGACAATTGACAA
Gbem_R7049	+	3969201	3969231	CAATTGACAATTGATAATTGACAATTGACGA
Gbem_R7050	-	3969397	3969427	CAATTGACGATTGACAATTGACAATTGACAA
Gbem_R7051	-	4070150	4070198	CAATTGACGATTGACAATGGACAATTGACAATGGACAATTGACAATGGA
Gbem_R7052	+	4163708	4163738	CAATTGACAATTGATAATTGACAATTGACAA
Gbem_R7053	+	4334146	4334176	CAATTGACAATGGACAATTGACGATTGACAA
Gbem_R7054	-	4334382	4334412	AAATTGACAATTGACAATGGACAATTGACAA
Gbem_R7055	-	4334443	4334473	GAATTGACGATTGACAATTGACGATTGACAA
Gbem_R7056	+	4418346	4418376	CAATTGACAATTGACGATTGACAATTGACAA
Consensus				CAATTGACAATTGACAATTGACAATTGACAATTGACAATTGACAATTGACAATTGACAA
Gbem_R7057	-	3174616	3174666	CAATTGACGGCGTAACCCAATTGACGGCGTAACCCAATTGACGGCGTAACC
Gbem_R7058	-	35641	35664	AACGTTGAACCTTGAACGTTGAAC
Gbem_R7059	+	35745	35768	AACATTGAACGTTGAACGTTGAAC
Gbem_R7060	+	190211	190234	AACCTTGAACGTTGAACCTTGAAC
Gbem_R7061	-	333601	333624	AACGTTGAACGTTGAACCTTGAAC
Gbem_R7062	+	333972	334002	AACGTTGAACTTTGAACCTTGAACCTTGAAC
Gbem_R7063	-	580480	580503	AACCTTGAACCTTGAACGTTGAAC
Gbem_R7064	-	581067	581090	AACGTAGAAGATAGAACGTAGAAC
Gbem_R7065	+	581170	581193	AACATTGAACGTTGAACGTTGAAC
Gbem_R7066	-	873384	873407	AACATTGAACGTTGAACGTTGAAC
Gbem_R7067	+	873568	873591	AACGTTGAACCTAGAACGTTGAAC
Gbem_R7068	+	873648	873671	AACGTTGAACGTTGAACGTTGAAC
Gbem_R7069	+	873695	873718	AACGTTGAACATTGAACGTTGAAT
Gbem_R7070	+	995293	995316	AACCTCGAACGGCGAACGTCGAAC
Gbem_R7071	-	1018974	1018997	AACGTTGAACGTTGAACCTTGAAC
Gbem_R7072	+	1019635	1019658	AACATTGAACGTTGAACCTTGAAC
Gbem_R7073	+	1462081	1462111	AACATCAAACGTTGAACGTAGAACGTTGAAC
Gbem_R7074	-	1474242	1474272	AACGTTGAACCTTGAACGTTGAACCTTGAAC
Gbem_R7075	+	1474302	1474325	AACGTTGAACTTCGAACGTTGAAC
Gbem_R7076	-	1854180	1854203	AACGTTGAACGTTGAACGTTGAAC
Gbem_R7077	+	1854282	1854312	TTCGTTGAACGTTGAACCTTGAACGTTGAAC
Gbem_R7078	-	2196208	2196231	AACGTCGAACGTTGAACGTTGAAC
Gbem_R7079	-	2196312	2196342	AAGGTTCAACGTTCAACGTTCAAGGTTCAAC
Gbem_R7080	-	2199930	2199953	AACGTTGAACGTTGAACGTTGAAC
Gbem_R7081	-	2200013	2200036	AACGTTCAACGTTCAACGTTAAAG
Gbem_R7082	+	2200053	2200076	AACGTAGAACATAGAACGTAGAAC
Gbem_R7083	+	2200097	2200120	AACGTTGAACGTTGAACGTTGAAC
Gbem_R7084	-	2656296	2656319	AACGTTGAACGTAGAACGTTGAAC
Gbem_R7085	+	2656375	2656398	AACGTAGAACATAGAACGTAGAAC
Gbem_R7086	-	2669303	2669326	AACGTAGAACGTAGAACGTTGAAC
Gbem_R7087	+	2669456	2669479	AACCTTGAACGTTGAACCTTGAAC
Gbem_R7088	+	3058312	3058335	AACGTCGAACGTTGAACATCGAAC
Gbem_R7089	+	3058379	3058409	AACGTCGAACGTTGAACGTCGAACGTTGAAC
Gbem_R7090	-	3235585	3235608	AACGTTGAACATTGAACGTTGAAC
Gbem_R7091	+	3235707	3235730	AACGTTGAACCTGGAACGTTGAAC
Gbem_R7092	+	3235792	3235815	AACGTTGAACCTGGAACGTTGAAC
Gbem_R7093	-	3838793	3838816	AACATTGAACGTAGAACGTTGAAC
Gbem_R7094	+	3838911	3838941	AATGTCGAACCTTGAACCTTGAACGTTGAAC
Gbem_R7095	-	3838956	3838979	AACGTAGAACGTTGAACGTTGAAC
Gbem_R7096	+	3839084	3839114	AATGTCGAACCTTGAACCTTGAACGTTGAAC
Gbem_R7097	+	3936869	3936892	AACGTTGAACGTTGAACGTTGAAC
Gbem_R7098	-	3962922	3962951	AACA-TGAACATTGAACGTTGAACGTTGAAC
Gbem_R7099	+	4460024	4460054	AACCTCGAACGTTGAACCCGGAACGTTGAAC
Gbem_R7100	-	4508169	4508199	AACGTTCAGCATCGAACGTTGAACGTTGAAC
Gbem_R7101	-	4508236	4508266	AACGTTGAACGTTGAACGTTGAACGTCGAAT
Gbem_R7102	+	4508348	4508378	AACGTTGAACATTGAACGTAGAACGTTGAAC
Consensus				AACGTTGAACGTTGAACGTTGAACGTTGAAC
Gbem_R7103	-	483882	483909	GGGATTAGGGACTAGGGGCTAGGGGCTG
Gbem_R7104	-	728938	728965	GGGGCTAGGGACTGGGGACTAGGGGCTG
Gbem_R7105	+	1562424	1562493	GGGGCTGGGGGCTGGGGGCTAGGGGCCAGGGGCTAGGGGCTAGGGGCTAGGGGCTAGGGGCTAGGGGTTG
Gbem_R7106	+	1776656	1776683	GGGGCTAGGGGCTGGGGACTGGGGGCTA
Gbem_R7107	-	1776704	1776731	GGGGCTAGGGGGCTAGGGGCCAGGGGCTG
Gbem_R7108	+	1844329	1844356	GGGGCTAGGGGGCTAGGGGCTGGGGACTG
Gbem_R7109	-	1844452	1844486	GGGGCTAGGGGTCGGGGGCTAGGGGCTGGGGGCTA
Gbem_R7110	-	1844508	1844542	GGGGCTAGGGGGCTAGGGGCTGGGGGCTGGGGGCTG
Gbem_R7111	-	4046541	4046568	GGGACTGGGGGCTAGGGGCTAGGGGCTG
Consensus				GGGGCTRGGGGCTRGGGGCTRGGGGCTRGGGGCTRGGGGCTRGGGGCTRGGGGCTRGGGGCTRGGGGCTR
Gbem_R7301	-	301149	301240	CCCGCGTCACCTCTTAATCTCAA
				TCTGCCTCACCTCTTAATCTCAA
				TCTGCCTCACCTCTTAATCTCAA
				TCTGCTTCACCTCTTAATCTGCC
Consensus				TCTGCYTCACCTCTTAATCTCAA
Gbem_R7302	-	614201	614284	CAAAAAACATGGCCTTACAAA
				ACAAAAATATGGTCTTACAAA
				AGGAAAAATAGGTCTTACAAA
				ACGAAAAACAGGTTCAACAAA
Consensus				ANRAAAANNWGGTCTTACAAA
Gbem_R7303	-	861774	862194	GTACGAGTTTCATATCGC-GAT
				GTGCTGGTTACATACTGC-GAT
				GTGCTGGCTACATATCGC-GAT
				GTGCTGGTTACATACTGC-GAT
				GTGCTGGTTACATACCGC-GAT
				GTGCTGGCTACATACCAC-GAT
				GTGCTGGTTACATACCAC-GAT
				GTGCTGGTTACATACCAC-GAT
				GTGCTGGTTACATACCGC-GAT
				GTGCTGGCTACATACCGC-GAT
				GTGCTGGCTACATACCGC-GAT
				GTGCTGGTTACATATCGC-GAT
				GTGCTGGTTACATATCGC-GAT
				GTGCTGGTTACATATCGC-GAT
				ATGCTGGTTACATACCGC-GAT
				GTGCTGGCTACATACCGC-GAT
				GTGCTGGTTACATACCGC-GAT
				GTGCTGGCTACATACCGC-GAT
				GTGCTGGCTACATACCA-TGAT
				GTATGAGTTACATACCGCTGAT
Consensus				GTGCTGGYTACATACCGC GAT
Gbem_R7304	+	1607736	1607977	AACGTCATTTTCCCC-GCT-CA
				GATGTCACTTTCCTC-TCGCAA
				AGTAACGCTTGGCAG-AAA-AA
				AACGTCGCTTTCTGC-GTCCAA
				AGCATCACTTGGAAC-GAA-AA
				AATATCACTTGAAGC-AAA-AA
				AACGTCACTTTTTGCGCCCAAA
				AGCATCACTTGGAGC-AAA-TA
				AACATCACTTGGGCG-TAAAAA
				ATGATCACTTGGACG-CAA-AA
				AATGTCGTTTCGTGG-GGG--A
				-ATGTCACTCGCCGG--AA-AA
Consensus				AAYRTCACTTKSNSS NMA AA
Gbem_R7305	-	1705909	1706323	AAGTTGTCCGCGATGATCTGT
				AACTTACACATCGCGGTATGT
				AAGTCATGCATCGCAACGAGT
				AACTCATGCACCGCGGTATGT
				AATTCACACATCGCGTTATGC
				TACTCGCACAGTGCAGTGTGA
				TACCCACATACCGCAGTCAGT
				AATTCATACACCGCAGCATGT
				AACATTTGCATCGCTCTCTGT
				AAACTACTTGGCGCGATGTAT
				AATTCGCACATCGCAATATGT
				ATTGCATACACCGCGGTATGT
				AACCAGCACATTGCAACAAGT
				AATTCATGCACCGCGGTATGT
				AACTCATGCACTGCGGTATGT
				ATCTCATGCATCGCGGTATGT
				GAATTACACATCGCGGTATGT
				AATTCGCACATCGTGGTATGT
				AATTCGTATATTGCGGTGGGT
				AACAGGCATACAGCAA
Consensus				AAYTCRYACAYCGCRRTATGT
Gbem_R7306	-	2206710	2206856	TGACCAAGTTGTTCGACGACT
				TGACCAAGTTGTTCGAGGACT
				TGCCTAAGTTGTTCGAGGACT
				TGCCTAAGTTGTTCGAGGACT
				TGCCTAAGTTGTTCGAGGACT
				TGATCAGGTTGTTCGAGGACT
				TGACCGAGTTGTTCGGAAAAT
Consensus				TGMCYAAGTTGTTCGAGGACT
Gbem_R7307	-	2314373	2314582	AAACCAAACTGAATTGGGGTC
				AAATCAAACTGATTTAGGGTC
				AAATCAAACTGATTCGGGGTC
				AAATCAAACTGATTCGGGGTC
				AAATCAAACTGATTCGGGGTC
				AAATCAAACTGATTCGGGGTC
				AAATCAAACTGATTCGGGGTC
				AAATCAAACTGATTCGGGGTC
				AAATCAAACTGATCCGGGGTC
				GAATCAAACTGATTTGGGTGA
Consensus				AAATCAAACTGATTYGGGGTC
Gbem_R7308	-	2368063	2368461	ATGGGCGCGTTTCAATTTGTC
				ATGACCGCGTGCCAATTTGTC
				ATGGACGCGTTGCAATTTGTC
				ATGGACGCGTTGCAATTTGTC
				ATGACCGCGTGCTAATTTGTC
				CTGACCGCGTGCTAATTTGTC
				CTGACCGCGTGCTAATTTGTC
				GTGACCGCGTGCAAGTTTGTC
				ATGACCGCGTTGCAATTTGTC
				ATGGGCGCGTTGCAATTTGTC
				ATGGGCGCGTGCTAATTCATC
				ATGAGCTCGTGCTAATTTGTC
				ATGAGCACATGACAAATTGAC
				CTGAGCGCGTGACAAGTTGTC
				ATGGGGGTGTGTCAATTCATC
				ATGAGCGCGTGCCAAGTTGTC
				ATGAGCTCGTGACAAAATTAC
				CTGAGCACATGACAAGTTAAC
				CTGGGCACCTTTCAGATTGTC
Consensus				MTGRSCGCGTKNYAATTTGTC
Gbem_R7309	-	2942185	2942541	ACGAAACTCGTACCCGCGACT
				CCGAAACTCGTACCCGCGACT
				CCGAAACTCGTACCCGCGACT
				CCGAAGCTCGTACCTTCGACT
				CCGAAGCTCGTACCTTCGACT
				CCGAAGCTTGTACCTTCGACT
				CCGAAGCTCGTACCCCCGACT
				GCGAAGCTCGTACCCGCGACT
				CCGAAGCTCGTACCTTCGACT
				CCGAAGCTCGTACCTTCGACT
				CCGAAGCTCGTACCTTCGACT
				GCGAAGCTCGTACCTTCGACT
				CCGAAGCTCGTACCCCCGACT
				GCGAAGCTCGTACCCTCGACT
				ACGAAGCTCGTACCCGCGACT
				GCGAAGCTTATACCCACGACT
				GCGAAGCTCGTACGCCCGCCT
Consensus				SCGAAGCTCGTACCYNCGACT
Gbem_R7310	-	3211707	3212126	ACGACGCTTACACCCCCGATT
				CCCACGCTTGTACTCACGACT
				ACGAAGCTCGTACCTTCGACT
				ACGAAGCTCGTACCTTCGACT
				ACGAAGCTCGCACCCACGACT
				ACGAAGCTCGTACCCATGACT
				ACGAAGCTCGCACCCACGACT
				ACGAAGCTCGCACCCACGACT
				ACGAAGCTCGCACCCACGACT
				ACGAAGCTCGCACCCGCGACT
				ACGAAGCTCGCACCCACGACT
				ACGAAGCTCGCACCCACGACT
				ACGAAGCTCGCACCCACGACT
				ATGAAGCTCGCACCCGCGACT
				ACGAAGCTCGCACCCGCGACT
				ACGAAGTTCGTACCCACGACT
				ACGAAGCTCGTACCCGGGACT
				ACGAAGTTCGTACCCGCGCCT
				ACGAAGCTCGTACCCGCGACT
				ACGAAGGGCGTGTGAAGGATT
Consensus				ACGAAGCTCGYACCCRCGACT
Gbem_R7311	-	4101404	4101612	TGAAACACGATGACGAATTGTCGGGAGATGGGGACGTTTAGT
				TGAAAGGCGATGACGAATTGTCGGGAGATGGGGACGTTTAGC
				TGAAACGCGATGACGAATTGTCGGGATATGGGGACGTTTAGC
				TGAAACGCGATGACGAATTGTCGGGAGATGGGGACGTTTAGT
				TGAAAGGCGATGACGAATTGTCGGGAGATG-GGATGTTTAAT
Consensus				TGAAASGCGATGACGAATTGTCGGGAGATGGGGACGTTTAGY
Gbem_R7312	-	4200080	4200305	CAATTTCACGTTCCAATTTGTCACGACCCCACGACAAATTAG
				CATGCGCCCGTTTCAATTTGTCACGACCTCACGACAAATTAG
				CACGCGCCCGTTTCAATTTGTCACGACCTCACGACAAATTAG
				CACGCGCCCGTTTCAATTTGTCACGACCTCACGACAAATTAG
				CACGCGCCCGTTCCAATTTGTCATGGCCTCACGACAAATTAC
				CATGCGCAAGTTGCAA
Consensus				CAYGCGCMCGTTYCAATTTGTCACGACCTCACGACAAATTAG
Gbem_R7313	-	4449311	4449664	TGATTATTCAAAGCCAATTTG
				AAATTGCACAATGCCGAAAGC
				GTTTGTGCAATTTCCATTTCG
				GTTTTGACTATTCATGAGTC-
				AATTTTGCTATTCCCGAATCG
				-ATTTAACTATTCCGGCGACA
				AAATAAGCTAATCCGAAATCG
				-ATTTAACTATTCCTGCGACA
				AAATAGGCTATTTCCAAATCG
				AAATAGGCTATTCCCAAACTG
				AAATAAGCTAATCCCAAACAG
				AAATAAGCTATTTCAACTGGT
				AAATAAGCTATATGCAGATGG
				AAATAGGCTATTTCCATCTGG
				GATTTGCTTAATTGCGTCAGG
				AATTAACAAATTTGCATTCTG
				ATCTGGTAACATTGCATATAG
Consensus				AAWTWRNCTAWTYSCRWWWSG
Gbem_R7314	-	4495455	4495626	GGCTACGGCCGGCAGGCCC--CCAA
				GGCTACGGCCGGCAAGCCC--CCAA
				GG-TACGGCCGGCAAGCCCCACCAA
				GGCTACGGCCGGCAAGCCC--CTAA
				GGCTACGGCCGGCAAGCCCCACCAA
				GGCTACGGCCGGCAGGCCC--CTAA
				GGCTATGGCCGGCAAGCCC-GCTAA
				GGCTACG------------------
Consensus				GGCTACGGCCGGCARGCCC CYAA
Gbem_R7315	-	4524706	4524872	GGAGCGAAAACGTCGTATTTT
				TTTTCAAATGTGTCATTTTTG
				AATTCAAATACGTCATATTCG
				GACTCAAAAATGACATATTCG
				GATTCAAAAACGTCATATTCG
				AGTTCAAAAACGACACATTT-
				TTTTCAAATGCGTCACTTTTG
				GATTCAAAAATGACATATTCA
Consensus				NNTTCAAAWAYGWCATATTYG
