# Supplementary material for: The genome of Geobacter bemidjiensis, exemplar for the subsurface clade of Geobacter species that predominate in Fe(III)-reducing subsurface environments
Source: BMC Genomics. 2010 Sep 9;11:490. doi: 10.1186/1471-2164-11-490 (PMC2996986; doi:10.1186/1471-2164-11-490)
Supplement: Additional file 15 — Figure S9. Multicopy nucleotide sequences of the G. bemidjiensis genome: base coordinates and alignments. (See also Table S6.). [file 1471-2164-11-490-S15.PDF]

Gbem_R9001	+	90240	90266	TTTTCCTCTGTGG-CCCTCTGTG	GTGAG
Gbem_R9002	+	90297	90341	TTAACCTCCGTG--ACCTCCGAGA-ACCTCTGAG-TCCTCTGTG	TTCCG
Gbem_R9003	-	197073	197099	TTTTCCTCTGTG-TACCTCTGTG	GTGAG
Gbem_R9004	+	197477	197514	TTTTCCTCTGTGGTTCCTCTGTG-TTCCTCTGTG	GTGAG
Gbem_R9005	+	200950	200985	AAAACCTCCGTGG-ACCTCCGAG--ACCTCTGTG	TTCCG
Gbem_R9006	+	201009	201046	TTTTCCCCTGTGGTTCCTCTGTG-TACCTCTGTG	GTGAG
Gbem_R9007	-	484908	484943	TTTTCCTCTGTG-TACCTCTGTG--TCCTCTGTG	GTGAG
Gbem_R9008	+	488065	488102	TTTCCCTCTGTGGTTCCTCTGTG-TTCCGCTGTG	GTGAG
Gbem_R9009	-	554402	554446	AAAACCTCTGTG--GCCTTCGTGA-ACCCCCGTG-CCCTCTGTG	TTCCG
Gbem_R9010	-	554466	554492	CCTTCCTCTGTG-TTCCTCTGTG	GTGAG
Gbem_R9011	-	606542	606577	TTTTCCTCTGTG-TACCTCTGTG--TCCTCTGTG	GTTAA
Gbem_R9012	+	606632	606676	GTTTCCTCTGTG--ACCTCCGTGA-TCCTCAGCG-ACCTCTGTG	TTCCG
Gbem_R9013	+	735457	735492	TTTTCCTCTGTG-TACCTCTGTG--TCCTCTGTG	GTGAG
Gbem_R9014	+	738416	738451	TTCTCCTCTGTGA-ACCTCTGTG--TCCTCTGTG	GTGAA
Gbem_R9015	-	983156	983182	TTTTCCTCTGTGA-ACCTCTGTG	GTGAG
Gbem_R9016	+	1203540	1203576	TTTTCCTCTGTGA-ACCTCTGTG-TCCCTCTGTG	GTGAG
Gbem_R9017	+	1805400	1805444	TTTTCCTTTGTG--ACCTCCGTGG-ACCTCAGTG-ACCTCTGTG	TTCCG
Gbem_R9018	-	1961324	1961359	TTTTCCTCTGTG-TACCTCTGTG--TCCTCTGTG	GTAAG
Gbem_R9019	+	1961418	1961453	GCGACCTCCGTGA-TCCTCTGTG--ACCTCTGTG	GTAAA
Gbem_R9020	-	1962689	1962726	TTTTCCTCTGTGGTTCCTCAGTGG-ACCTCTGTG	GTGAG
Gbem_R9021	+	1962785	1962811	TTTTCCTCCGTGA-ACCTCTGTG	GTAAA
Gbem_R9022	+	1962827	1962863	TTTAGCTCTGTGGTTCCTCTGTG--TCCTCTGTG	GTGAA
Gbem_R9023	-	2193466	2193501	TTTTCCTCTGTG-TTCCTCTGTG--TCCTCTGTG	GTGAA
Gbem_R9024	+	2193561	2193598	CTTTCCTCTGTGGTTCCTCTGTG-TACCTCTGTG	GTGAG
Gbem_R9025	+	2207972	2208016	AAAACCTCGGTG--ACCTCCGTGG-ACCTCAGTG-CCCTCTGTG	TTCCG
Gbem_R9026	-	2228912	2228956	AATACCTCAGTG--ACCTCCGTGG-ACCTCCGCG-ACCTCTGTG	TTCCG
Gbem_R9027	-	2327293	2327337	AAAACCTCAGTG--CCCCCCGTGG-ACCTCCGAG-ACCTCTGTG	TTCCG
Gbem_R9028	-	2327358	2327395	CTTTCCTCTGTGCTTCCTCTGTG-TACCTCTGTG	GTGAG
Gbem_R9029	-	2344648	2344692	CTTCTCTCTGTG--ACCTCCGTGA-ACCTCCGCG-ACCTCTGTG	TTCCG
Gbem_R9030	-	2351346	2351390	AAAACCTCGGTG--ACCTCCGTG-TACCTCCGAG-ACCTCTGTG	TTCCG
Gbem_R9031	-	2351411	2351448	TTTTCCTCTGTGGTTCCTCTGTG-TACCTCTGTG	GTGAG
Gbem_R9032	+	2351513	2351549	TTTTCCTCTGTGA-CCCTCTGTG-TCCCTCTGTG	GTGAA
Gbem_R9033	-	2428713	2428757	TTTTCCTCCGTG--ACCCCCGTGA-ACCTCCGAG-TCCTCTGTG	GTCAT
Gbem_R9034	+	2553018	2553054	TTTTCCTCTGCG-TACCTCTGTG-TTCCTCTGTG	GTGAG
Gbem_R9035	-	2583549	2583613	AAAACCTCGGTG--ACCTCCGTGG-ACCTCCGTGGACCTCCGTGGACCTCCGTGACCTCTGCGTTCCG	
Gbem_R9036	+	2583812	2583848	CTTTCCTCTGTG-TACCTCTGTG-TCCCTCTGTG	GTGAG
Gbem_R9037	+	2700104	2700148	TTTTCCTCTGTG--CCCTCAGTGA-ACCTCCGGG-ACCTCTGTG	TTCCG
Gbem_R9038	+	2863183	2863208	ATTTCCTCTGTG--TCCTCTGTG	GTGAA
Gbem_R9039	+	2900866	2900903	CTTTCCTCTGTGGTTCCTCTGTG-CACCTCTGTG	GTGAG
Gbem_R9040	+	2900928	2900972	AAAACCTCTGTG--ACCTCCGTGA-ACCTCCGCG-ACCTCTGTG	TTCCG
Gbem_R9041	-	2903121	2903156	TTTTCCTCTGTG-TCCCTCTGTG--TCCTCTGTG	GTGAA
Gbem_R9042	+	2903279	2903316	TTTTCCTCTGTGGTTCCTCTGTG-TTCCTCTGTG	GTGAG
Gbem_R9043	+	2946609	2946645	TTCTCCTCTGTG-TCCCTCTGTGA-ACCTCTGTG	GTGAA
Gbem_R9044	-	3057437	3057481	AAAATCTCTGTG--ACCTCCGTGG-ACCTCAGCG-ACCTCTGTG	TTCCG
Gbem_R9045	-	3093391	3093448	CTTTCCTCTGTGGTTCCTCTGTG-TACCTCTGTGTACCTCTGTGTACCTCTGTG	GTGAG
Gbem_R9046	+	3093506	3093532	TTTTCCTCTGTGA-TCCTCTGTG	TTCGC
Gbem_R9047	+	3335484	3335510	TCTTCCTCTGTG-TCCCTCTGTG	GTGAG
Gbem_R9048	+	3335530	3335574	AAAACCTCTGTG--GCCTCCGTGA-ACCTCTGCG-ACCTCTGTG	TTCCG
Gbem_R9049	-	3424718	3424762	AAAACCTCTGTG--CCCTCCGTGG-ACCTCCGCG-ACCTCTGTG	TTCCG
Gbem_R9050	-	3424783	3424820	GTTGCCTCTGTGTTTCCTCTGTGA-ACCTCTGTG	GTGAG
Gbem_R9051	+	3424869	3424903	TTTTCCTCTGTG--TCCTCTGTG--TCCTCTGTG	GTGAA
Gbem_R9052	+	3424940	3424974	TTTTCCTCTGTG--CCCTCTGTG--TCCTCTGTG	GTGAA
Gbem_R9053	-	3486981	3487006	CGAACCTCCGTG--ACCTCTGTG	TTCCG
Gbem_R9054	-	3487047	3487084	TTTTCCTCTGTGGTTCCTCTGTG-TACCTCTGTG	GTGAG
Gbem_R9055	-	3706107	3706133	TTCTCCTCTGTG-TACCTCTGTG	GTGAG
Gbem_R9056	-	3808536	3808572	CCTTCCTCTGTG-TACCTCTGTG-TACCTCTGTG	GTGAA
Gbem_R9057	+	3808620	3808656	TTTTACCCTGTG-CTCCTCTGTG-TACCTCTGTG	GTGAT
Gbem_R9058	-	3880498	3880533	TTTTCCTCTGTG-AGCCTCTGTG--TCCTCTGTG	GTGCA
Gbem_R9059	-	4075378	4075413	TTTTCCTCTGTG-ATCCTCTGTG--TCCTCTGTG	GTGAA
Gbem_R9060	+	4075471	4075508	CTTTCCTCTGTGGTTCCTCTGTG-TACCTCTGTG	GTGAT
Gbem_R9061	+	4075530	4075574	AAAACCTCAGTG--TCCTCAGTG-TACCTCCGTG-ACCTCTGTG	TTCCG
Gbem_R9062	+	4175995	4176033	TTTTCCTCTGTGTATCCTCTGTGATCCCTCTGTG	GTGAG
Gbem_R9063	-	4309132	4309176	AAAACCTCTGTG--ACCTCCGTGG-ACCTTCGAG-ACCTCTGTG	TTCCG
Gbem_R9064	-	4309196	4309233	CCTTCCTCTGTGATTCCTCTGTG-TACCTCTGTG	GTGAG
Gbem_R9065	+	4309292	4309318	TTTTCCTCTGTGA-TCCTCTGTG	GTGAA
Gbem_R9066	-	4511546	4511590	AAAACCTCAGTG--GCCTCCGTGA-ACCTCTGCG-ACCTCTGTG	TTCCG
Gbem_R9067	-	4603073	4603108	TTTTCCTCTGTG-TACCTCCGTG--TCCTCTGTG	GTGCA
Gbem_R9068	+	4603152	4603189	TTTTCCTCTGTGATTCCTCTGTGA-CCCTCTGTG	GTGAG
Consensus				TTTWCCTCTGTG WCCTCTGTG WCCTCTGTG WCCTCTGTG WCCTCTGTGWCCTCTGTG	GTGAG

Gbem_R9069	-	488008	488056	AAAAGCTCTAAACAGGGATGAAAGGGATACAGGGGATAACTGCATAAAA
Gbem_R9070	-	1112803	1112851	AAAGGCATTTAACAGGGATGAAGGGGATGGAAGGGATAGAGCAGGGACA
Gbem_R9071	+	1856019	1856067	AAAAACGTTTAACAGGGATAAATGGGATACAAGGGATAACTGCAGAACC
Gbem_R9072	-	1856096	1856144	TTAATCCATTTACAGGGATGAAGGGGATGAATGGGATAAGACCAAAAAC
Gbem_R9073	+	1856183	1856231	AAAAGCATTTAACAGGGATACAAGGGATACAGGGGATAACGGCAAAGGC
Gbem_R9074	+	1895955	1896003	AAAACCTATTCACAGGGATGAAGGGGATACAGGGGATAACGGCAAAACC
Gbem_R9075	-	1896059	1896107	AAAGGCTCTCTACAGGGATGAAGGGGATGAAAGGGATAAAATCCCTGTT
Gbem_R9076	-	1902706	1902754	CGAATCATTTAACAGGGATGAAGGGGATAAAAGGGATAAAACCTTAAAC
Gbem_R9077	+	1909106	1909154	AAACATATTTAACAGGGATGAAGGGGATAAACGGGATAAATCACAACCA
Gbem_R9078	+	1910112	1910160	CAACCCCTTTTACAGGGATGAAGGGGATAAAAGGGATAAAACCAAGGAA
Gbem_R9079	-	2266204	2266252	CAAGGCATTGAACAGGGATGAAGGGGATACAGGGCCGTTGCAGTTTTAG
Gbem_R9080	-	2552965	2553013	GCAGGCATTTCACAGGGATGAAGGGGATACAGGGGATAACTGCAAAAAC
Gbem_R9081	+	2903167	2903215	AAAAACGTGGAACAGGGATGAAGGGGATAAAGGGGATAACTGCAAAAAA
Gbem_R9082	+	2946483	2946531	AAAAACACCTAACAGGGATGAAGGGGATACAGGGGATAACTACAAACCA
Gbem_R9083	-	3292301	3292349	AAACCATTTGAACAGGGATGAAGGGGATAAACGGGATAACCGCGAACTC
Gbem_R9084	+	3418500	3418548	CAAGGCTTTAAACAGGGATGAAGGGGATACAGGGGATAACTGCAAAAGC
Gbem_R9085	-	3426508	3426556	GAAAAGCTTTTACAGGGATGAAGGGGATAAAGGGGATAACTGCAAAGGC
Gbem_R9086	+	3582428	3582476	AAAGACGTTTAACAGGGATGAAGGGGATAAATGGGATATTCAAAACCTT
Gbem_R9087	-	3582484	3582532	AAAGGCGATCTACAGGGATGAAGGGGATGGAAGGGATAAAGACAAAAAC
Gbem_R9088	-	3880614	3880662	GCACAGGGATGAAGGGGATACAGGGGATAACTGCAAAAACAAAGAACAT
Gbem_R9089	+	3971064	3971112	AAAAACATTTAACAGGGATAAAGGGGATAAAAGGGATAAAACCGGAAAC
Gbem_R9090	-	3971142	3971190	AAAACCGACTCACAGGGATGAAGGGGATGCAGGGGATAACTGCGAAAAC
Consensus				AAARRCNTTTAACAGGGATGAAGGGGATAMARGGGATAAMWNCRAAAAM

Gbem_R9091	-	1171566	1171610	GTGGACCTGGTGGACCTGGTGAACCTGGTGGACCTCATGTACTGC
Gbem_R9092	-	1840647	1840682	GCGGACAGAATGGACAGAATGGACAGAATGGGGATG
Gbem_R9093	-	1844536	1844580	ATGGACGTAATGGACGTAATGGACGTAATGGACTAAATGGGGCTA
Gbem_R9094	+	2254414	2254512	ATGGACTTGATGGACTTGATGGACCTGATGGACCTGATGGACCTGATGGACCTGATGGACCTGATGGACCTGATGGACCTGATGGACCTGATGGACCAC
Gbem_R9095	-	3072625	3072759	GAGGACCCAATGGACCCAATGGACCCAATGGACCCAATGGACCCAATGGACCCAATGGACCCAATGGACCCAATGGACCCAATGGACCCAATGGACCCAATGGACCCAATGGACCCAATGGACCCAATGGACCAG
Gbem_R9096	-	4289045	4289215	TCCGACCTGTCTGACCTGTCCGACCTGTCTGACCTGTCTGACCTGTCTGACCTGTCTGACCTGTCTGACCAGTCTGACCTGTCTGACCTGTCTGACCTGTCTGACCTGTCTGACCTGTCCGACCAGTCCGACCAGTCCGACCAGTCCGACCAGTCCGACCAGTCCGACCAG
Gbem_R9097	+	1495921	1495992	TCTGACCAGTCTGACCAGTCTGACCAGTCTGACCTGTCGGACGGGTCTGACCTGTCTGACCTGTCGGACGAG
Consensus				WYGGACCTGWYGGACCTGWYGGACCTGWYGGACCTGWYGGACCTGWYGGACCTGWYGGACCTGWYGGACCTGWYGGACCTGWYGGACCTGWYGGACCTGWYGGACCTGWYGGACCTGWYGGACCTGWYGGACCTGWYGGACCTGWYGGACCTGWYGGACCTGWYGGACCTG

Gbem_R9098	+	1703572	1703589	AATCCGCGTCATCCGCGT
Gbem_R9099	-	3324223	3324254	TGCCCCCGATGCCCCCGATGCCCCCGATGCCC
